# Higher‐Level Structural Classification of Pseudomonas Cyclic Lipopeptides through Their Bioactive Conformation

**DOI:** 10.1002/advs.202520365

**Published:** 2025-12-14

**Authors:** Benjámin Kovács, Durga Prasad, Vic De Roo, Matthias Vanheede, Penthip Muangkaew, Annemieke Madder, Monica Höfte, René De Mot, Niels Geudens, José C. Martins

**Affiliations:** ^1^ NMR and Structure Analysis Unit Department of Organic and Macromolecular Chemistry Faculty of Sciences Ghent University Ghent 9000 Belgium; ^2^ Organic Biomimetic Chemistry Research Group Department of Organic and Macromolecular Chemistry Ghent University Ghent 9000 Belgium; ^3^ Laboratory of Phytopathology Department of Plants and Crops Faculty of Bioscience Engineering Ghent University Ghent 9000 Belgium; ^4^ Centre for Microbial and Plant Genetics Faculty of Bioscience Engineering KU Leuven Heverlee‐Leuven 3001 Belgium

**Keywords:** conformational analysis, left‐handed α‐helix, non‐ribosomal biosynthesis, pseudomonas lipopeptides, stapled peptides

## Abstract

Cyclic lipodepsipeptides (CLiPs) from *Pseudomonas* are membrane‐targeting specialized metabolites with diverse ecological roles and antimicrobial activities. Over the past decades, significant efforts have been made to reveal their chemical constitution and configuration, thus providing the starting point to establishing structure–function correlations, deriving molecular‐level understanding of their mode of action, and ultimately harnessing their potential in plant biocontrol and clinical applications. The sheer diversity in chemical structures, combined with a few scattered reports of 3D structures, has limited advances in these areas. The solution conformations of eight antimicrobial, non‐phytotoxic *Pseudomonas* CLiPs, each representing a distinct family, are presented, obtained using a consistent NMR and molecular dynamics protocol in dodecylphosphocholine micelles. All CLiP conformations share a left‐handed α‐helix forming a stapled or catch‐pole helix motif depending on the number of residues in the macrocycle. This structural dichotomy is validated through a synthetic analogue of the naturally occurring orfamide A featuring an alternative, more constricted macrocycle. The two motifs define distinct superfamilies encompassing most known *Pseudomonas* CLiPs, offering a new, coherent framework for their structural classification that is also reflected in the organization of their biosynthetic gene cluster. The findings support future homology modelling and molecular design efforts for these metabolites.

## Graphical abstract

Solution structures of a series of cyclic lipodepsipeptides from *Pseudomonas* reveal that these specialized metabolites adopt left‐handed alpha helical conformations of only two types, being either ‘stapled’ or ‘catch‐pole’ helix. The particular modular distribution of the biosynthetic gene clusters is found to determine the extent of their conserved secondary structural elements.

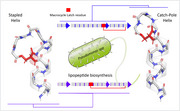

## 1 Introduction

Cyclic lipodepsipeptides (CLiPs) feature prominently amongst the steadily growing collection of specialized metabolites retrieved from *Pseudomonas*, a bacterial genus ubiquitously found in soil, water and plants. CLiPs are produced by non‐ribosomal peptide synthetases (NRPS) encoded in biosynthetic gene clusters (BGCs) that are found throughout three major pseudomonad clades (Fluorescens, Putida, and Syringae) but absent from the Aeruginosa lineage, including the opportunistic human pathogen *P. aeruginosa*.^[^ ^1^ ^]^ CLiPs are important agents taking part in the chemically mediated microbial ecology of their producers.^[^ ^2^, ^3^, ^4^ ^]^ Specifically, CLiPs have been shown to control swarming motility, contribute to biofilm formation, while also mediating interactions with other microorganisms, nematodes, algae and plants, amongst other effects.^[^ ^5^, ^6^, ^7^ ^]^ Many CLiP‐producing *Pseudomonas* strains from the aforementioned groups are well adapted to interact with plants, either in the rhizosphere, or above ground in the phyllosphere, or as endophytes. Depending on the particular species and the CLiP(s) it produces, either harmful or beneficial effects can be observed for the colonized plant. The beneficial effects of CLiPs, which may include induced systemic resistance, often appear to be connected to their antimicrobial activities and probably evolved to ward off microbial competitors competing for the same niche as the CLiP producer. As a result, these CLiPs and their producers have been put forward as potential biocontrol agents.^[^ ^8^, ^9^, ^10^, ^11^ ^]^ Despite a large chemical diversity, most plant‐beneficial CLiPs share the presence of at least one and sometimes two acidic residues and the absence of basic residues, making these negatively charged at physiological pH. Concurrently, some of the *Pseudomonas* CLiPs classified as members of the Mycin and Peptin families have a dual role in microbial antagonism and phytotoxicity. Strains producing these CLiPs operate at the borderline between pathogenic and beneficial interactions with plants, the outcome being determined by the specific combination of CLiP producer and eukaryotic host in a given ecological setting.^[^ ^5^ ^]^ Interestingly, Peptins feature one or more basic residues and no acidic ones, making these positively charged, while Mycins have an ambivalent character as they feature both acidic and basic residues. Thus, based on ionizable residue contents, three distinct clusters of CLiPs families can be considered. Here, we will focus on the cluster of negatively charged, acidic CLiPs representing diverse families generally not contributing to virulence in plants. For convenience, we introduce the term ‘Acidilins’ to refer to this major cluster.

Given their antimicrobial effects, these plant‐beneficial producers and their Acidilin metabolites are of considerable interest in two application areas. First, being non‐phytotoxic, they can offer new biopesticide solutions for plant biocontrol that satisfy the regulatory demand for alternatives to chemical pesticides and meet rising concerns regarding food security.^[^ ^12^ ^]^ Second, the urgent need for novel antibiotics and antifungals due to rising antimicrobial resistance provides additional impetus to develop *Pseudomonas* Acidilins as valuable lead compounds. Antimicrobial functions reported for these CLiPs include anti‐oomycete, antiviral, antifungal and antibacterial activity, while insecticidal, antiprotozoal and anticancer properties have also been described in specific cases. Acidilins thus hold significant promise for advancing fundamental research into microbial ecology and crop protection, while providing new leads for the development of bioactive agents that could benefit human health directly or indirectly.

Still, much ground remains to be covered to better understand the molecular basis of their mode of action and ultimately harness their potential. Numerous studies of individual CLiPs have indicated the cell membrane as the primary target, whereby permeabilization of the cell membrane leads to transient or permanent leakage events, ultimately resulting in cell death, typically at high nm to low µm concentrations depending on the assay and CLiP used.^[^ ^2^, ^13^, ^14^, ^15^ ^]^ The membrane permeabilizing properties emerge from the amphipathic character associated with their common chemical blueprint. *Pseudomonas* CLiPs feature an oligopeptide sequence that is N‐capped by an acyl chain and C‐capped by depsi (lactone) bond formation through condensation with the hydroxyl group of the side‐chain of a preceding amino acid residue, which therefore creates a latch closing the macrocycle. Although assembled by the NRPS from a limited number of mostly proteinogenic amino acid substrates, the lipopeptidome of *Pseudomonas* based on this blueprint features a large chemical structure diversity. A majority of residues display d‐configuration by virtue of dual condensation/epimerization domains active in the NRPS assembly line.^[^ ^16^ ^]^ Chemical variations on the common theme include the oligopeptide chain length, the site of cyclization, the configuration (d/l) of incorporated amino acids, the amphipathic sequence pattern and the nature of the acyl chain, all of which are encoded at the level of the respective BGC. Additional diversity within a specific *Pseudomonas* species results from flexibility upon amino acid and/or acyl chain substrate recruitment from the metabolite pool,^[^ ^17^ ^]^ and even one notable case of d/l configurational flexibility resulting in the generation of diastereomeric CLiPs from the same NRPS complex.^[^ ^18^ ^]^

Previous studies have used genomic and taxonomic investigations as a basis for CLiP classification. The progressive accumulation of planar chemical structures in literature prompted us to propose a classification based on tags reporting the oligopeptide length (*l* = total number of residues) and macrocycle size (*m* = number of residues in the cycle). CLiPs with identical (*l*:*m*) tag sharing the same oligopeptide sequence amphipathicity profile are then grouped in a single CLiP family named for a representative member.^[^ ^19^ ^]^ Diversity within a family arises from differences in fatty acid chain structure and/or conservative residue substitutions. This has, so far, allowed a more manageable classification of the more than 100 CLiPs described to date into 17 distinct families (Figure S1, Supporting Information), which assisted the chemotaxonomy of CLiPs, and contributed to identifying evolutionary drivers of NRPS diversification within and between lipopeptide families from comparative BGC analysis and scrutiny of NRPS domain architecture. The Acidilins are represented by 10 out of the 17 (*l*:*m*) CLiP families and display a large variety in both *l* (8–14) and *m* (4–10) values.

However, with few exceptions, the challenges in accurately determining the stereochemistry of CLiPs have hindered progress toward structure‐activity relationship studies and the elucidation of their 3D structure. With our recently introduced and broadly applicable workflow integrating chemical synthesis, bioinformatic analysis and NMR‐based fingerprinting, these difficulties have been cleared for most *Pseudomonas* CLiPs.^[^ ^19^ ^]^ Their stereochemistry clarified, we here present the conformations for eight Acidilins, each representing a distinct CLiP (*l*:*m*) family with antimicrobial and non‐phytotoxic effects, namely the Bananamides^[^ ^20^, ^21^ ^]^ (8:6), Viscosins^[^ ^22^, ^23^, ^24^, ^25^, ^26^, ^27^, ^28^, ^29^, ^30^ ^]^ (9:7), Orfamides^[^ ^31^, ^32^, ^33^, ^34^, ^35^, ^36^, ^37^ ^]^ (10:8), Amphisins^[^ ^38^, ^39^, ^40^, ^41^, ^42^, ^43^, ^44^, ^45^ ^]^ (11:9), Tanniamides^[^ ^46^ ^]^ (12:10), Putisolvins^[^ ^47^, ^48^ ^]^ (12:4), Entolysins^[^ ^49^ ^]^ (14:5) and Xantholysins^[^ ^50^ ^]^ (14:8), determined in micellar solutions of membrane‐mimicking dodecylphosphocholine (DPC). All CLiPs feature a left‐handed α‐helix (α_L_‐helix) and adopt either a ‘stapled‐helix’ or ‘catch‐pole helix’ conformational motif, depending on the number of residues in the macrocycle (*m*). When *m*>5, the molecule consistently adopts the stapled α‐helix motif. This structural dichotomy was validated by pentorfamide A (10:5), a synthetic analogue of orfamide A (10:8) with a contracted macrocycle, demonstrating the influence of macrocycle size on the overall conformation. These two conformational motif types define distinct superfamilies encompassing most known *Pseudomonas* CLiPs, providing higher‐order classification and confrontation with biosynthetic gene cluster (BGC) organization. Our proposal provides new footholds for structure–activity relationship studies, homology modelling, and biophysical investigations of this class of *Pseudomonas* specialized metabolites.

## 2 Results and Discussion

### 2.1 Selection of Representative CLiPs for Structure Determination

**Figure 1:**
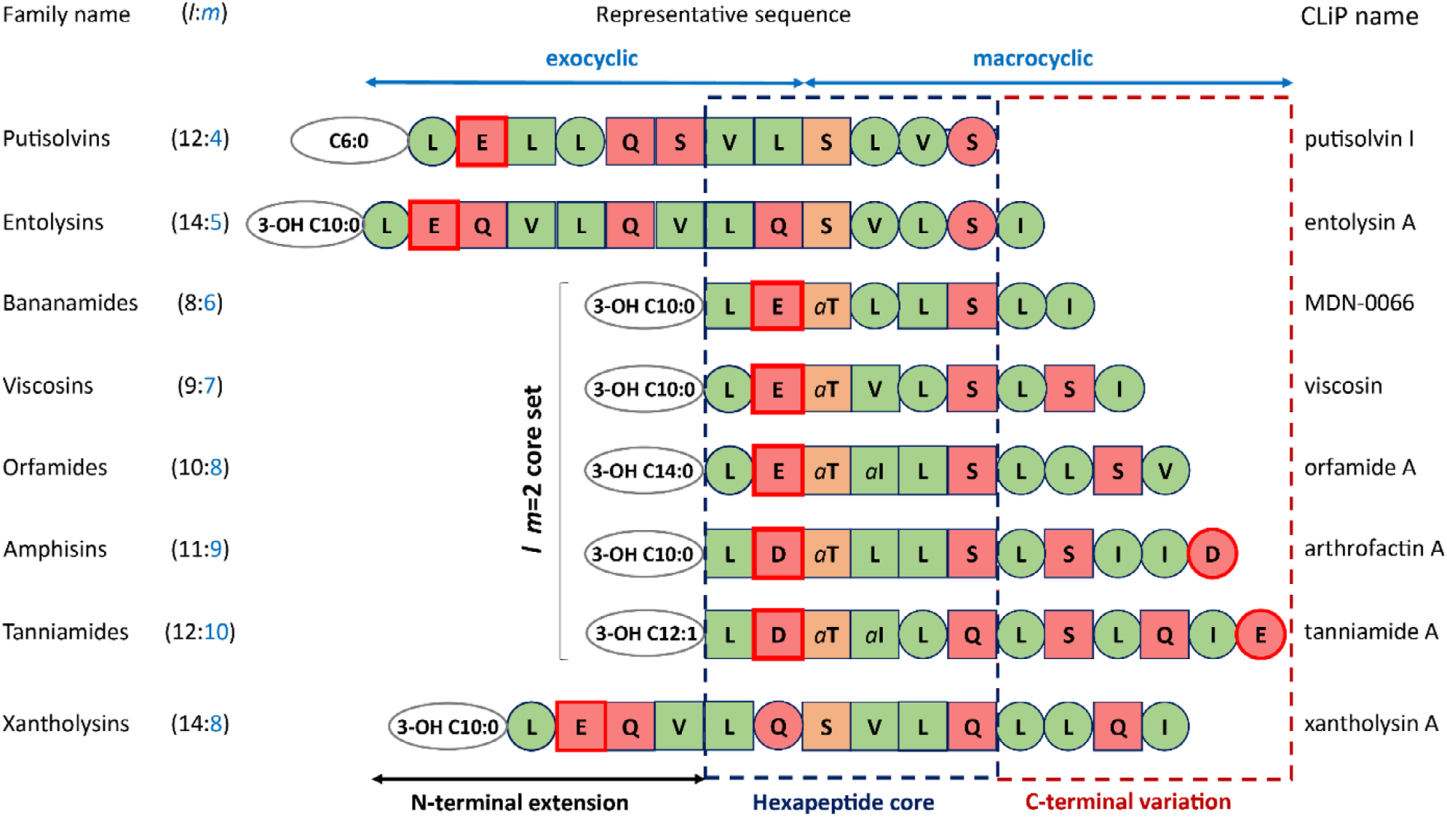
Alignment of the Acidilin CLiP sequences investigated in this work using the internal residue involved in the depsi bond with its side‐chain (d‐Ser or d‐ *allo*‐Thr) as a point of reference. Residues are represented with one‐letter codes as follows: circle: l‐amino acid; square: d‐amino acid; green: hydrophobic side‐chain; red: hydrophilic side‐chain; orange: depsi bonded side‐chain; ‘*a*’: *allo*‐side‐chain (for Thr or Ile); thick red contour: negatively charged side‐chain at physiological pH. The length and saturation level of the N‐terminal acyl chains together with the presence of a hydroxyl group attached to the (*R*‐configured) β‐carbon atom are also indicated (ovals). ‘C6:0’ = hexanoic acid; ‘3‐OH C10:0’ = (*R*)‐3‐hydroxydecanoic acid; ‘3‐OH C14:0’ = (*R*)‐3‐hydroxytetradecanoic acid; ‘3‐OH C12:1’ = (*R*)‐3‐hydroxydodec‐(*Z*)‐5‐enoic acid. CLiP families (left) are shown with first letter capitalized along with the corresponding *l*:*m* tags, while individual CLiP names (right) are indicated without capitalization, except for MDN‐0066 where the original code is maintained as published. The stereochemistry was derived as described elsewhere; see also Table 1.

In total, CLiPs from 8 different (*l*:*m*) families representing a broad sampling of Acidilins produced by pseudomonads from the Fluorescens and Putida groups, listed in **Table** **1**, were selected for conformation determination in DPC micelle solutions. The corresponding sequences collected in **Figure** **1** are aligned according to the position of the Ser/Thr residue, acting as the latch closing the macrocycle by ester bond formation with the C‐terminus, and clearly showcase the similarities and differences in constitution and amphipathic profile. The rationale in selecting these 8 CLiPs results from a number of initial considerations and subsequent developments in the course of this work as motivated in more detail in Section S1 (Supporting Information). By investigating CLiPs with macrocycle size *m* from 4 to 10, we cover all known macrocycle sizes amongst Acidilins as well as *Pseudomonas* CLiPs in general. A core set (MDN‐0066 (8:6), viscosin (9:7), orfamide A (10:8), arthrofactin A (11:9) and tanniamide A (12:10)) collects 5 CLiPs that have a fatty‐acid N‐capped exocyclic dipeptide segment (*l*–*m* = 2) in common, while two additional CLiPs (putisolvin I (12:4) and entolysin A (14:5)) allow to focus as well on the impact of longer exocyclic segments. Xantholysin A (14:8) completes the set in order to investigate whether macrocycle conformation is maintained across family boundaries as it shares identical macrocycle size with orfamide A (both have *m* = 8) but has a longer exocyclic oligopeptide. From here on we will omit congener specifications (e.g. A, B, I, etc.) unless required.

**Table 1:** The CLiP‐producing *Pseudomonas* strains with their taxonomic affiliations, RCSB Protein Data Bank (PDB) and Biological Magnetic Resonance Bank (BMRB) accession codes of the CLiP conformations determined in this work.

CLiP (*l:m*)^a)^	Producer	Phylogenetic group	Reference^b)^	PDB entry	BMRB entry
MDN‐0066 (8:6)	*P. azadiae* SWRI103	Fluorescens	[19, 21]	9EPS	34911
viscosin (9:7)	*P. fluorescens* SBW25	Fluorescens	[22]	8S2C	34903
orfamide A (10:8)	*P. protegens* Pf‐5	Fluorescens	[19, 31, 32]	8S4L	34905
arthrofactin A (11:9)	*Pseudomonas* sp. MIS38	Fluorescens	[38]	8Q1L	34841
tanniamide A (12:10)	*P. ekonensis* COR58	Fluorescens	[46]	8S8N	34907
putisolvin I (12:4)	*P. capeferrum* PCL1445	Putida	[46, 47]	8S2B	34902
entolysin A (14:5)	*P. entomophila* L48	Putida	[18, 49]	8S2D	34904
xantholysin A (14:8)	*P. mosselii* BW11M1	Putida	[19, 50]	8S2A	34901

^a)^ The (*l*:*m*) tag indicates *l* amino acid residues are present in the oligopeptide with *m* residues involved in the C‐terminal macrocycle ring and (*l*–*m*) exocyclic residues;

^b)^ Reference(s) to original literature reporting the isolation, structure elucidation and stereochemistry determination of the CLiP

### 2.2 Solution Structure Elucidation of CLiPs in DPC Solution

The membrane targeting mode of action of CLiPs prompted us to use solutions of DPC micelles, commonly used as membrane mimicking environment,^[^ ^51^, ^52^ ^]^ to explore their conformational properties and structure‐activity relationships. Zwitterionic DPC was chosen over anionic sodium dodecyl sulfate (SDS) as it is a milder detergent, but also to cover eukaryotic membranes as the activity range of these CLiPs includes antifungal and cytotoxic ones in addition to antibacterial effects. Solubilization of each CLiP in a phosphate buffer solution of deuterated DPC‐*d38* micelles (Table S1, Supporting Information) results in a single set of ^1^H resonances that appear uniformly broadened when compared to polar organic solvents. This supports the formation of CLiP:micelle assemblies, as further confirmed from the translational diffusion coefficients of the CLiP and the DPC micelles, independently determined using ^1^H, respectively ^31^P PFG‐NMR measurements.^[^ ^53^, ^54^, ^55^, ^56^ ^]^ (Section S2.5, Supporting Information) The values obtained support full partitioning into the micellar phase, as observed before in the case of viscosinamide (9:7).^[^ ^57^ ^]^ The determination of the micelle‐bound conformation of each CLiP proceeded in two stages, as described in more detail in Section S3 (Supporting Information). In the first stage, interproton distance restraints were obtained from the nOe cross‐peak intensities in the 2D NOESY spectra and used as input to generate a conformational ensemble using the CNS 1.21 software.^[^ ^58^, ^59^ ^]^ The availability, on average, of at least 11 distance restraints per residue with an average of 20% medium‐ and long‐range contacts overall, allowed to produce a single, well‐defined conformational ensemble for the peptide in all cases. (**Table** **2**) The total backbone root mean square deviation (RMSD) amongst the 10 lowest energy structures ranges from 0.10±0.02 Å (MDN‐0066 (8:6)) to 0.39 ± 0.06 Å (tanniamide (12:10)) without displaying any violations above 0.1 Å. Since nOe's to constrain the acyl chain beyond the C3 carbon were absent, the acyl chain conformation remained ill‐defined and not included in the RMSD calculation.

**Table 2:** nOe restraint and structure calculation statistics for M: MDN‐0066, V: viscosin, O: orfamide, A: arthrofactin, T: tanniamide, P: putisolvin, E: entolysin, X: xantholysin and PO: pentorfamide.

Ps‐CLiP (*l*:*m*)		M (8:6)	V (9:7)	O (10:8)	A (11:9)	T (12:10)	P (12:4)	E (14:5)	X (14:8)	PO (10:5)
nOe^a)^	intraresidual	48	45	40	53	73	42	72	94	55
	sequential (*i,i*+1)	33	32	33	53	63	34	57	74	23
	mid‐range (*i,i*+2/4)	14	22	16	32	57	20	32	70	16
	long‐range (*i*+5)	5	4	2	4	3	2	0	3	0
nOe‐total (nOe‐ambiguous^b)^)		111 (11)	109 (6)	115 (24)	156 (14)	203 (7)	132 (34)	201 (40)	310 (69)	103 (9)
CNS RMSD [Å]^c)^	Backbone [s.d.]	0.10 [0.02]	0.13 [0.05]	0.39 [0.04]	0.25 [0.12]	0.39 [0.06]	0.30 [0.08]	0.34 [0.08]	0.25 [0.13]	0.54 [0.27]
	heavy‐atom [s.d.]	0.39 [0.07]	0.64 [0.13]	0.57 [0.09]	0.51 [0.09]	0.58 [0.07]	0.78 [0.12]	0.75 [0.10]	0.57 [0.11]	0.78 [0.27]
Representative MD vs CNS structure RMSD [Å] Violations >0.5 Å^d)^		0.45 9%	0.75 3%	0.81 4%	0.81 5%	0.79 9%	0.39 3%	0.96 5%	1.11 5%	1.12 11%

^a)^ Distance restraints that included the acyl chain atoms are limited and were not considered for these statistics;

^b)^ In the absence of stereospecific assignments of methylene and methyl groups, nOe's involving these were treated as ambiguous restraints during calculations;

^c)^ RMSD values and standard deviations (s.d.) reflect the average of all pairwise RMSD values of individual CNS ensemble conformations with respect to the average conformation obtained after alignment to the lowest overall energy structure. The acyl chain atoms were omitted from alignment and RMSD calculation as they are not constrained by experimental data;

^d)^ The percentage of high nOe violations (>0.5 Å) of the refined MD structure. These were obtained by loading the MD refined (representative) structure into CNS and listing nOe violations against the restraint file with the violation threshold set to 0.5 Å. To avoid any changes in the MD refined structure by the CNS algorithm, all the default simulated annealing and structure optimization processes were omitted.

**Figure 2:**
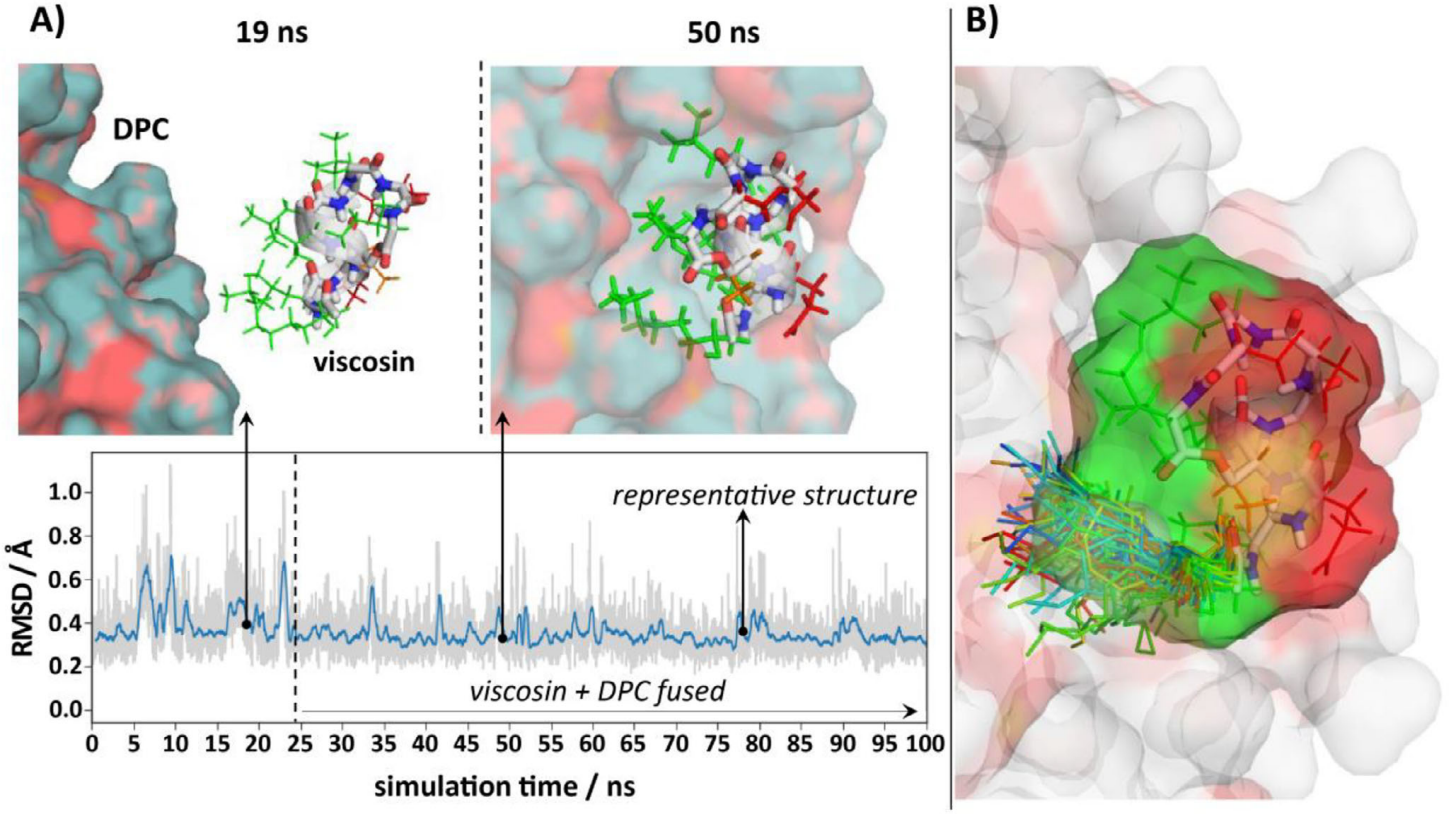
A) The MD refinement process of the CNS structure in explicit water:DPC environment illustrated with that of viscosin (9:7). The DPC micelle is shown in surface representation with water molecules omitted for clarity. Backbone is shown in ball and stick, with side‐chain in green sticks, and the α_L_‐helix highlighted by the grey ribbon. The evolution of peptide backbone RMSD with respect to the last frame structure is shown at the bottom (blue line: running average). After ≈25 ns simulation time (dashed line) the initially solvated viscosin molecule inserts itself on the surface of the DPC micelle with its hydrophilic (red) and hydrophobic (green) side‐chains oriented toward the aqueous phase and the core of the micelle, respectively. The frame of the representative structure (at ≈78 ns) is also indicated in the RMSD plot. B) While flexible, the CLiP acyl chain is restricted to the lipophilic site of the peptide always in an orientation suitable to interact with the micelle. To illustrate this, 100 conformations sampled by the acyl chain at uniform time intervals over the last 50 ns of the trajectory after N‐terminal alignment of the peptide backbones are shown. For clarity, a single viscosin structure is displayed only and hydrogen atoms are omitted from both the DPC molecules and the peptide acyl chains. See Figure S14 (Supporting Information) for similar representation of the other CLiPs.

In the second stage, as illustrated in **Figure** **2**, the quality of the lowest energy CNS structure from each ensemble was improved by subjecting it to a 100 ns unrestrained molecular dynamics simulation in an explicit water:DPC micelle environment using the AMBER ff14SB force field.^[^ ^60^ ^]^ To avoid placement bias of the CLiP at the water:DPC micelle interface and building on previous simulation experience with CLiPs in the matter,^[^ ^61^ ^]^ the initially fully solvated CLiP molecule was allowed to insert itself in the first 5–50 ns of the simulation. In all cases, insertion was maintained, and the CLiP oriented itself in line with its amphipathic nature, with hydrophobic amino acid side‐chains and acyl chain moiety facing the aliphatic micelle core and hydrophilic side‐chains oriented toward the water phase. This agrees well with results using paramagnetic relaxation enhancements for viscosinamide (9:7) in DPC micelles.^[^ ^57^ ^]^ On the micellar surface each CLiP displays a stable backbone conformation with limited flexibility and only transient distortions. (Figure S3, Supporting Information) A single representative conformation closest to the time‐averaged position of the atomic coordinates in the post‐insertion MD trajectory was selected using a cluster algorithm in order to illustrate and visually compare the respective CLiP conformations. As extensively documented in Section S4.2 (Supporting Information), such representative conformation populates energetically more favored regions of the Ramachandran plot than the CNS structures, while maintaining similar backbone conformation and good agreement with the experimental restraints in spite of these not being imposed, with limited violations above 0.5 Å (Table 2). In what follows, we will use these MD‐refined representative conformations to represent the solution structure used for individual and comparative analysis, together with data collected from the refinement trajectories. The CNS generated ensemble as well as the representative MD conformation, together with restraints files as well as chemical shift assignments of all 8 CLiPs are available from the PDB (RCSB Protein Data Bank, https://www.rcsb.org/) and BMRB (Biological Magnetic Resonance Bank, https://bmrb.io/) databanks, respectively, with accession codes as indicated in Table 1. The MD trajectories are available from the Zenodo data repository. (https://doi.org/10.5281/zenodo.15024231).

### 2.3 *Pseudomonas* CLiPs adopt Two Conformational Motifs Depending on Macrocycle Size

**Figure 3:**
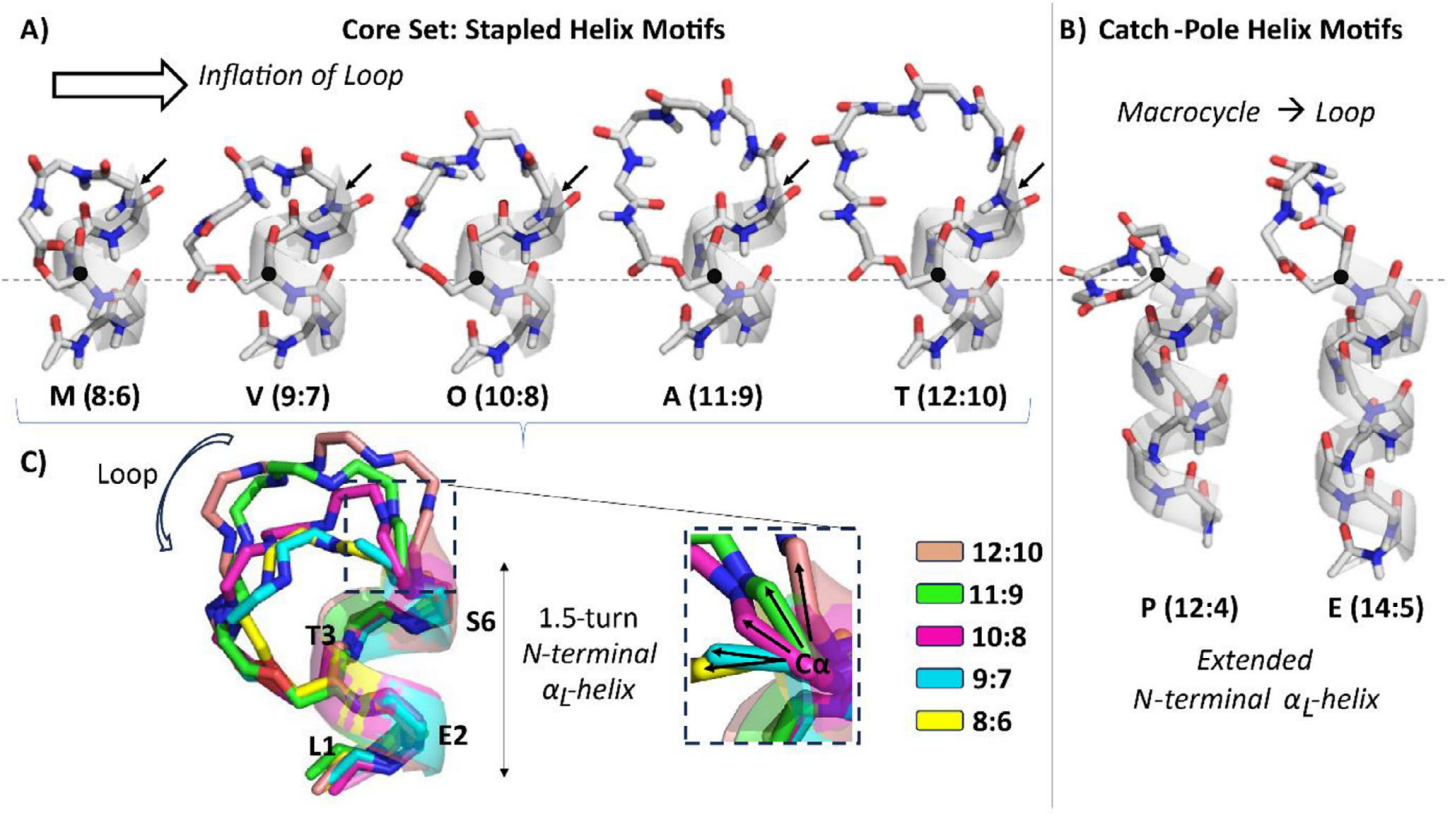
The obtained CLiP conformations (M: MDN‐0066, V: viscosin, O: orfamide, A: arthrofactin, T: tanniamide, P: putisolvin, E: entolysin) distinguished also by their respective *l*:*m* tag. The N‐terminal acyl chain is omitted as its conformation is ill‐defined. A) stapled‐helix motifs (*l*–*m* = 2 core set) and B) catch‐pole helix motifs. All structures are shown with the N‐terminus at the bottom, featuring the peptide bond connecting the fatty acid chain to the first residue. H^N^, C, N and O atoms are colored respectively white, grey, blue and red. Except for the depsi bond, the amino acid side chains and the acyl chain moieties are omitted for clarity. The structures are aligned horizontally at the respective Cα atom (black dot) of the residue involved in depsi bond formation via its side‐chain. For the stapled helices, the helix‐terminating amide plane (highlighted by arrows) might deviate from α‐helicity if the macrocycle is too short (*m*≤8). C) For the *l*–*m* = 2 stapled‐helix cores set the evolving local backbone conformation of the 6th residue is also illustrated through the reorientation of the Cα–C’ bond. The structures are aligned at the common N‐terminal α_L_‐helix and shown in multiple colors.

The backbone solution conformation of each of the 8 CLiPs is collected in **Figure** **3**, with oligopeptide length increasing from left to right. All CLiPs feature an α‐helix, beyond which a loop twists and turns to allow the macrocycle to close through depsi bond formation. Being composed mostly of d‐amino acids, the helix is left‐handed (α_L_) in all cases, a feature rarely observed in natural compounds. While the α_L_‐helix of arthrofactin consists entirely of d‐amino acids, all other CLiPs feature at least one l‐residue in their respective α_L_‐helices. The adoption of the α_L_‐conformation by an l‐configured residue either at the N‐terminus (orfamide, entolysin), in the body of the α_L_‐helix (MDN‐0066, tanniamide) or both (viscosin, putisolvin), while energetically less favorable,^[^ ^62^ ^]^ does represent an energy minimum in phi (*φ*), psi (*ψ*) Ramachandran space for non‐β‐branched side‐chains such as the leucines involved here in all cases. (Figures S5–S12, Supporting Information) Notably, in these CLiP structures their presence does not significantly distort the backbone of the α_L_‐helix.

Focusing on the core set of 5 CLiPs with *l*–*m* = 2 (Figure 3A), their N‐terminal ≈1.5 turn α_L_‐helix extends into the macrocycle as highlighted by the backbone ribbon in their respective solution structure. It is stabilized by *i*,*i*+4 hydrogen bonds involving the NH at position 4 and 5, with the CO of the acyl chain and Leu1 respectively. These hydrogen bonds were previously established as long‐lived hydrogen bonds using long‐range HNCO experiments applied to ^13^C,^15^N labeled viscosinamide (9:7).^[^ ^57^ ^]^ The helix effectively coincides with the common hexapeptide core sequence in Figure 1, thus representing a conserved structural element within the core set. Deviations from α_L_ in excess of 10° for phi of d‐ *allo*‐Thr3 and the psi angle of the preceding acidic d‐residue likely arise from the involvement in macrocyclization of the d‐ *allo*‐Thr side‐chain via the depsi bond formation. (See phi/psi angles of all CLiP conformations in Tables S12–S16, Supporting Information) The departure from left‐handed helicity is complete when reaching Leu7, as its negative phi, psi values support a right‐handed α‐helical (α_R_) conformation. From here onward, the macrocycle residues form a loop that attaches midway onto the α_L_‐helix. The C‐terminus is so to speak ‘stapled’ onto the body of the helix, leading us to designate this common conformational motif within the core set of CLiPs as the ‘stapled helix’ motif. The term ‘stapling’ was introduced with an approach developed for the metathesis‐mediated connection of two unnatural hydrocarbon side chains in a short peptide, entailing the stabilization of the helical fold.^[^ ^63^, ^64^ ^]^ Since its introduction, the concept of side chain‐to‐side chain stapling was frequently applied in medicinal chemistry approaches toward not only conformational but also metabolic stabilization using a plethora of chemistries to connect the correctly positioned (*i*,*i*+4 or *i*,*i*+7) side chains in short peptides.^[^ ^65^ ^]^ More recently, backbone‐to‐backbone stapling approaches have expanded the portfolio.^[^ ^66^, ^67^ ^]^ In the current core set of CLiPs stapling occurs by depsi bond formation involving the Ser or Thr side chain and the C‐terminus of the peptide, likely also contributing to improved helix stability. Indeed, hydrolysis of the depsi bond, producing the linear CLiP variant leads to their inactivation, possibly due to the loss of their specific structural organization.^[^ ^68^, ^69^ ^]^ This naturally occurring stapling method that does not find any currently established synthetic precedent, has the potential to inspire a new class of side chain‐to‐backbone stapled peptides.

As the number of residues in the macrocycle increases from *m* = 6 to *m* = 10 in the core set (CLiPs with *l–m* = 2), the loop lengthens and adopts a conformation uniquely characteristic for each of these CLiP family, as evident from the phi, psi angles and the superposition of the CLiP structures in Figure 3C. (See Table S15, Supporting Information for direct comparisons.) As the spatial distance between the helix terminus and the stapling point remains constant, the loop conformation adapts to accommodate the varying number of residues in the macrocycle, causing the loop to expand as *m* increases. With four macrocycle residues already involved in the α_L_‐helix, the residues available to close the macrocycle loop equal *c = m–*4 and evolve from 6 in tanniamide (12:10) to only 2 in MDN‐0066 (8:6). Here, two l‐configured residues in the loop are likely required to facilitate its closure as the adopted phi, psi values are energetically less amenable to d‐residues. The strain caused by the shortening of the loop is apparent from the evolution in phi, psi values of the helix terminating Leu5 and d‐Ser6/d‐Gln6 residues (vide supra). Visually this is best reflected by the reorientation of the Ser6/d‐Gln6 Cα–C’ bond as shown in Figure 3C, as it is rotated from being parallel to almost perpendicular with the main helical axis.

The prominent change in conformational motif observed when the number of residues making up the macrocycle is reduced below *m* = 6 comes as no surprise, therefore. While the longer exocyclic oligopeptide present in putisolvin (12–4 = 8) and entolysin (14–5 = 9) extends the left‐handed α‐helix from 2.5 to almost 3 turns, respectively, the α_L_‐helix no longer partakes in the macrocycle. (Figure 3B; Table S16, Supporting Information) Rather, the macrocycle forms a distinct loop that bulges out from the helical cylinder, creating an overall ‘catch‐pole’ appearance. We propose to refer to a structuration of this type as a catch‐pole helix motif, which distinguishes itself from the stapled‐helix motif by a macrocycle that forms a separate, closed loop at the end of the helix rather than being stapled to its side. Like in stapled‐helix CLiPs, the presence of l‐Leu residues at position 1 (both CLiPs) and 4 (putisolvin) is well tolerated and does not disrupt the α_L_‐helix, which is also characterized by an extended network of *i*,*i*+4 hydrogen bonds in both CLiPs. The helix is well maintained and terminates with the d‐Ser residue acting as the latch closing the macrocycle. In putisolvin, an *i*,*i*+3 hydrogen bond between d‐Val7 CO and l‐Leu10 NH causes the C‐terminal end of the helix to be capped by the macrocycle. In entolysin, capping involves an *i*,*i*+4 hydrogen bond between d‐Val7 CO and d‐Val11 NH, causing a different spatial orientation of the macrocycle with respect to the helix, as evident from Figure 3B. Fitting an ellipsoid to the macrocycle backbone, its long axis has 140° (entolysin) versus 45° (putisolvin) angle with the main axis of the helix. (Figure S13, Section 4.3, Supporting Information)

**Figure 4:**
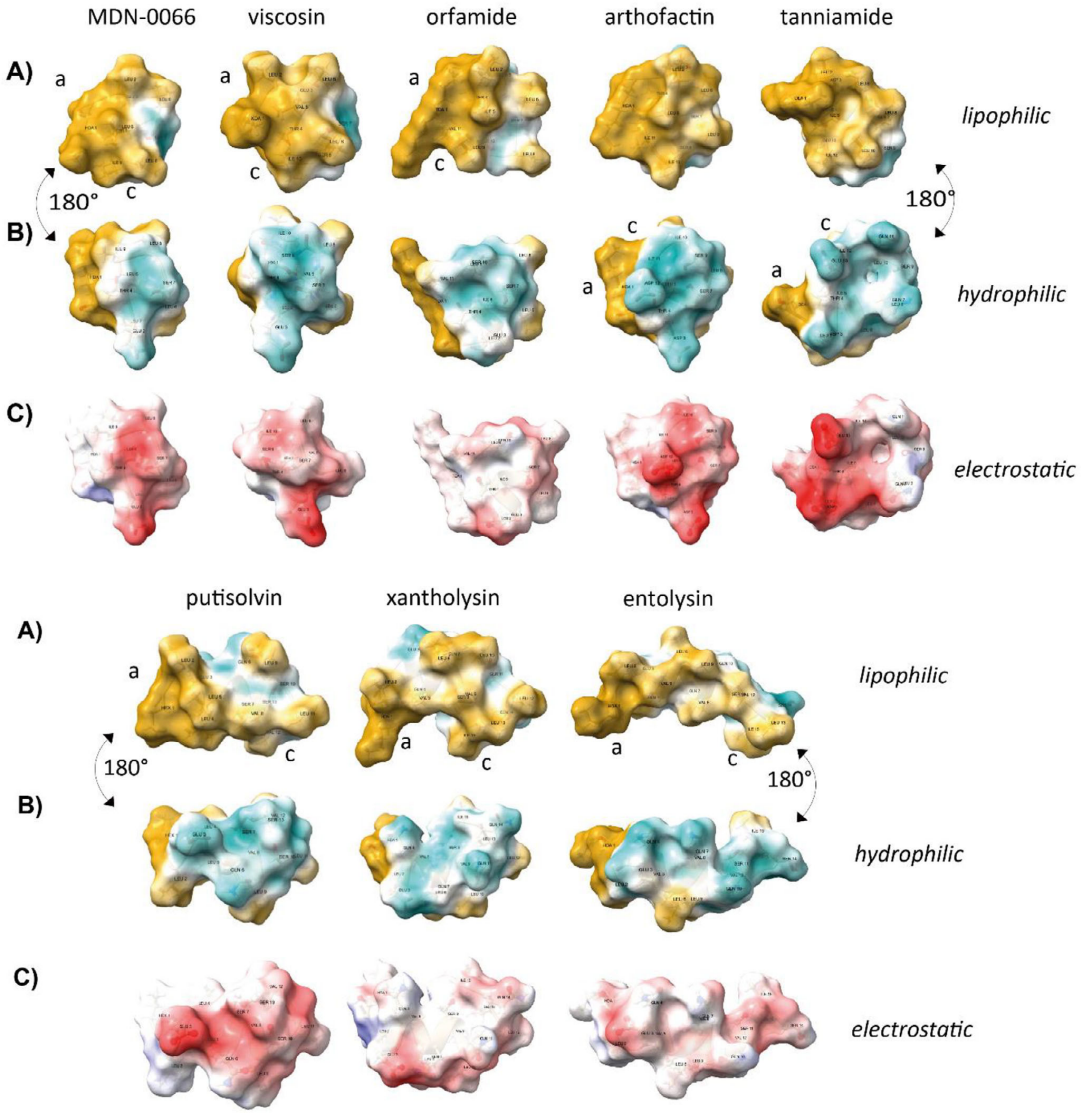
Molecular surface views of MDN‐0066, viscosin, orfamide, arthrofactin, tanniamide, putisolvin, xantholysin, entolysin generated from their derived conformations. To guide the eye labels “a” and “c” were placed close the acyl chain and respectively C‐terminal amino acid Cα atom. A) Surfaces colored by molecular lipophilicity potential (MLP) highlight the predominantly lipophilic face (gold), with the structures oriented accordingly. The acyl chain, although conformationally dynamic, consistently aligns along the lipophilic face. B) The same structures rotated 180° along the vertical axis to display the hydrophilic face (cyan/white), illustrating the spatial separation between hydrophobic and polar regions. This dual‐face orientation demonstrates the amphipathic organization, in which lipophilic and hydrophilic surfaces remain well‐separated regardless of macrocycle size or sequence variation. C) Electrostatic surface potentials, colored according to Coulombic potential (red: negative, blue: positive), are shown for the hydrophilic face only, as the opposing lipophilic face exhibits minimal electrostatic variation and provides limited additional insight. The clustering of acidic residues, particularly at position 2 and, when present, at the C‐terminus (arthrofactin, tanniamide), contributes to directional charge distribution across the molecular surface.

3D surface maps of the CLiPs including the acyl chain and color coded according to molecular lipophilicity and Coulomb electrostatic potentials as shown in **Figure** **4** clearly illustrate the amphipathic nature of all CLiPs. (See also Section S4.4, Supporting Information) Irrespective of the macrocycle length ‘*m*’ the lipophilic and hydrophilic faces of the α_L_‐helix are continued without disruption in the macrocycle by virtue of the specific combination of D/L configurations and residue types. Even though the conformation of the acyl chain is ill‐defined, the ensemble space they occupy is always placed along the lipophilic face of the molecules (Figure 2B; Figure S14, Supporting Information). The second acidic residue (Asp/Glu) present at the C‐terminus in arthrofactin and tanniamide clearly co‐localizes with that of the acidic residue always present at position 2, creating a negatively charged patch. Altogether, the well‐separated lipophilic and hydrophilic faces strongly favor interaction with the membrane. The positioning of the CLiPs at the water:DPC interface, as revealed by the MD trajectories (Figure 2), further supports this, as also quantified by the number of water molecules within a 5.0 Å sphere centered on each amino acid Cα (Section S4.5, Supporting Information). As one would expect, maxima occur for hydrophilic residues and minima for lipophilic ones, causing a wave‐like pattern for residues involved in the α_L_ helix. (Figure S15, Supporting Information) Finally, we note that the short exocyclic sequence in the CLiPs belonging to the core set creates quite compact, rather spherical amphiphilic structure, while the longer exocyclic sequence in putisolvin, entolysin (and also xantholysin, vide infra) leads to more elongated ones.

### 2.4 One CLiP Conformation Appears Sufficient to Define a (*l*:*m*) CLiP Family

The conformations of putisolvin (12:4), entolysin (14:5), MDN‐0066 (8:6), orfamide (10:8) and tanniamide (12:10) are the first conformations to be determined for their respective CLiP (*l*:*m*) family, while those of viscosin and arthrofactin expand those already described for other members of the Viscosin (9:7) and Amphisin (11:9) family respectively. With backbone RMSD values ranging from 0.41 to 0.53 Å (Figure S16, Supporting Information), the viscosin conformation, determined in micellar DPC solution, superimposes well with the crystal structures of pseudophomin,^[^ ^26^ ^]^ pseudodesmin,^[^ ^27^ ^]^ WLIP^[^ ^29^ ^]^ and the solution structure of viscosinamide^[^ ^30^ ^]^ obtained from acetonitrile solutions. A similar observation holds for arthrofactin (11:9) and the crystal structures of other Amphisin family members, amphisin,^[^ ^40^ ^]^ tensin^[^ ^41^ ^]^ and anikasin^[^ ^42^ ^]^ with the RMSD values ranging from 0.34 to 0.47 Å. (Figure S17, Supporting Information) The quasi‐identical backbone conformations amongst the members of the same families demonstrate that conservative changes in the amino acid type within these families do not significantly affect the overall conformation and surface characteristics. Therefore, knowledge of the conformation of a single CLiP for a particular (*l:m*) group appears generally sufficient to provide the structural template for all its family members.

### 2.5 CLiP Families with the Same *m*‐Value Adopt Similar Macrocycle Conformations

**Figure 5:**
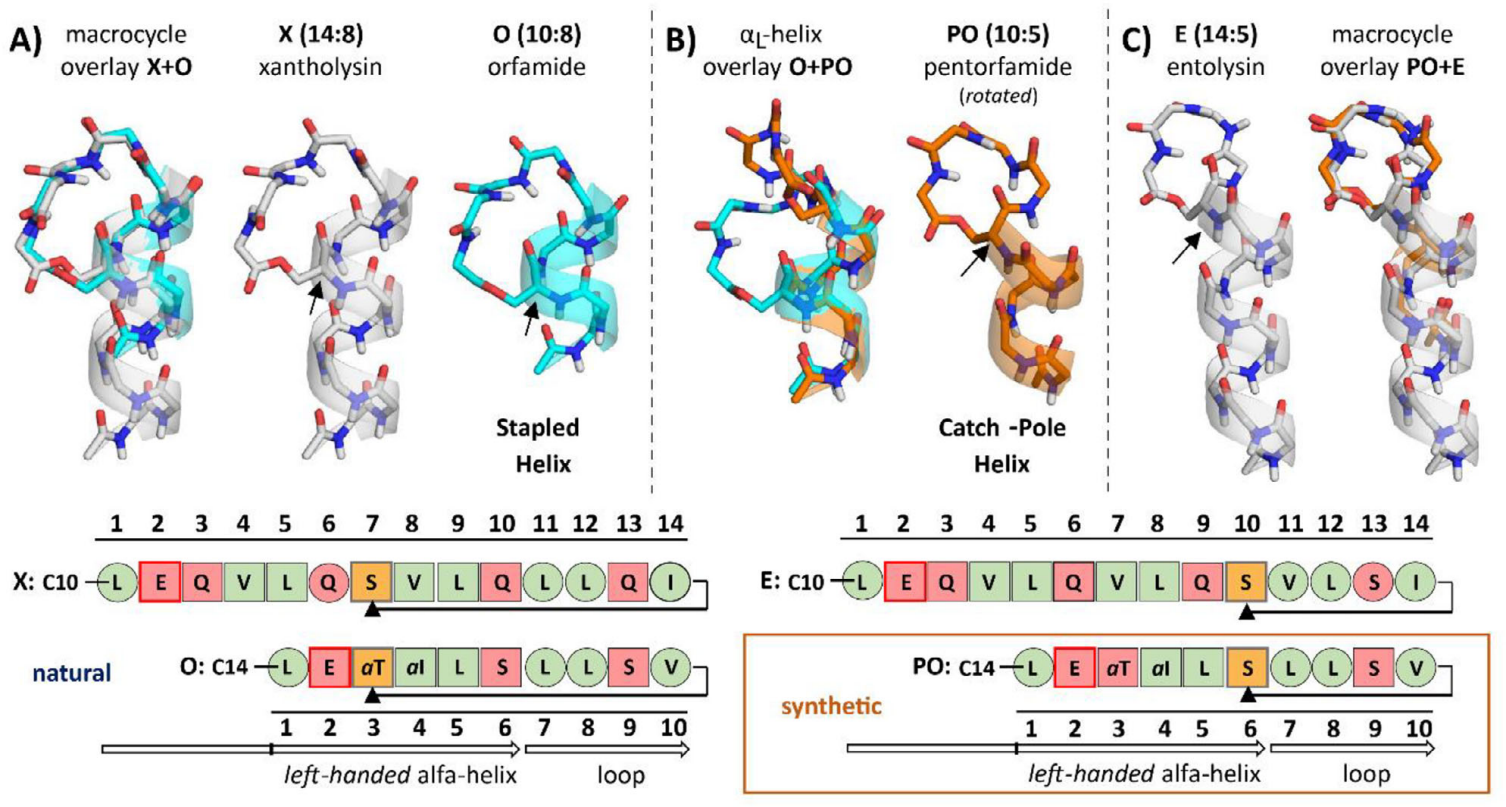
Structural comparisons of A) xantholysin (X, carbon atoms: grey) vs orfamide (O, turquoise); B) pentorfamide (PO, orange) vs orfamide and C) pentorfamide vs entolysin (E, grey). All structures are shown with the N‐terminus at the bottom. The acyl chain moieties are omitted for clarity. The corresponding oligopeptide sequences are aligned at the C‐terminus. The sequence representation codes are described in Figure 1. The depsi bonding latch residues (Cα) are highlighted by arrows both in the structures as well as the oligopeptide sequence representations.

The length of the exocyclic oligopeptide mainly modulates the length of the amphipathic α_L_‐helix, being 2 residues in CLiPs from the core set up to 9 residues in entolysin. This drew our attention to the Xantholysin (14:8) family and its similarity to the Orfamide (10:8) family. With a common macrocycle size of *m* = 8, their sequences differ substantially at the amino acid level as 5 out of 8 amino acids are different. Most notably, the d‐ *allo*‐Thr latch residue in orfamide is replaced by a d‐Ser in xantholysin, whereas glutamines replace both serines. Nevertheless, pairwise sequence alignment anchored to the respective latch residue shows an almost conserved configuration (except l‐Gln6 vs d‐Glu2) and a fully conserved amphipathicity profile in the C‐terminal decapeptide, as shown in **Figure** **5**. We therefore predicted that xantholysin would adopt the stapled‐helix motif as observed for orfamide, with similar macrocycle conformation and the four extra residues contributing to a full turn N‐terminal extension of the α_L_‐helix. This is indeed what is found as the conformation of the macrocycle of xantholysin A (14:8) shows excellent overall agreement with that of orfamide (10:8) and the additional α_L_‐turn maintains the amphipathic character as evident from the surface representation in Figure 4. (See also macrocycle phi/psi angles of the two structures in Table S25, Supporting Information) This establishes that CLiPs from different families having identical *m*‐value adopt highly similar macrocycle conformations provided the backbone configuration of the macrocycles is the same.

### 2.6 Switching Motifs Through Macrocycle Modulation

To validate that macrocycle size *m* determines the conformational motif type of α_L_‐helical CLiPs we synthesized and determined the conformation of pentorfamide (10:5) in micellar DPC solution. Pentorfamide (10:5) features a sequence identical to that of orfamide A (10:8), where the macrocyclisation topology is altered by closing the macrocycle at d‐Ser6 rather than d‐ *allo*‐Thr3. (Figure 5, bottom) The synthesis protocol followed that of pseudodesmin A (9:7) developed earlier^[^ ^70^ ^]^ (see in detail in Section S2.2, Supporting Information), and the same conformation determination workflow was applied as for the natural CLiPs (see analysis of the pentorfamide conformation in Section S5, Supporting Information). By reducing the *m*‐value from 8 to 5, a switch from stapled helix to catch‐pole loop motif is expected and indeed observed. (Figure 5B) The original 1.5‐turn α_L_‐helix remains maintained up to the d‐Ser6 latch residue, but inow completely exocyclic. Pentorfamide adopts the same *m*‐value as entolysin (14:5), and indeed, their macrocycles show identical orientation with respect to the helix axis. (Figure 5C and see ellipsoid fitting in Figure S13, Supporting Information) At the level of phi, psi backbone torsion angles, however, the pentorfamide and entolysin macrocycles display more significant differences compared to the orfamide/xantholysin case above, an almost 180° flip of the 2nd peptide bond in the macrocycle being the most notable. (Table S28, Supporting Information) Likely, this results from the combined effect of the configuration inversion for the penultimate residue and a leucine replacing the β‐branched valine when comparing pentorfamide to entolysin, thus generating an alternative lowest energy macrocycle conformation that preserves the overall amphipathic surface in spite of the configurational inversion. (Figure S19, Supporting Information) This shows that different CLiPs with the same macrocycle size but different macrocycle residue configuration(s) may adopt alternative backbone conformation in the macrocycle to maintain a particular amphipathic surface profile.

### 2.7 Correlation of CLiP Conformation with Producer BGC/NRPS Organization

**Figure 6:**
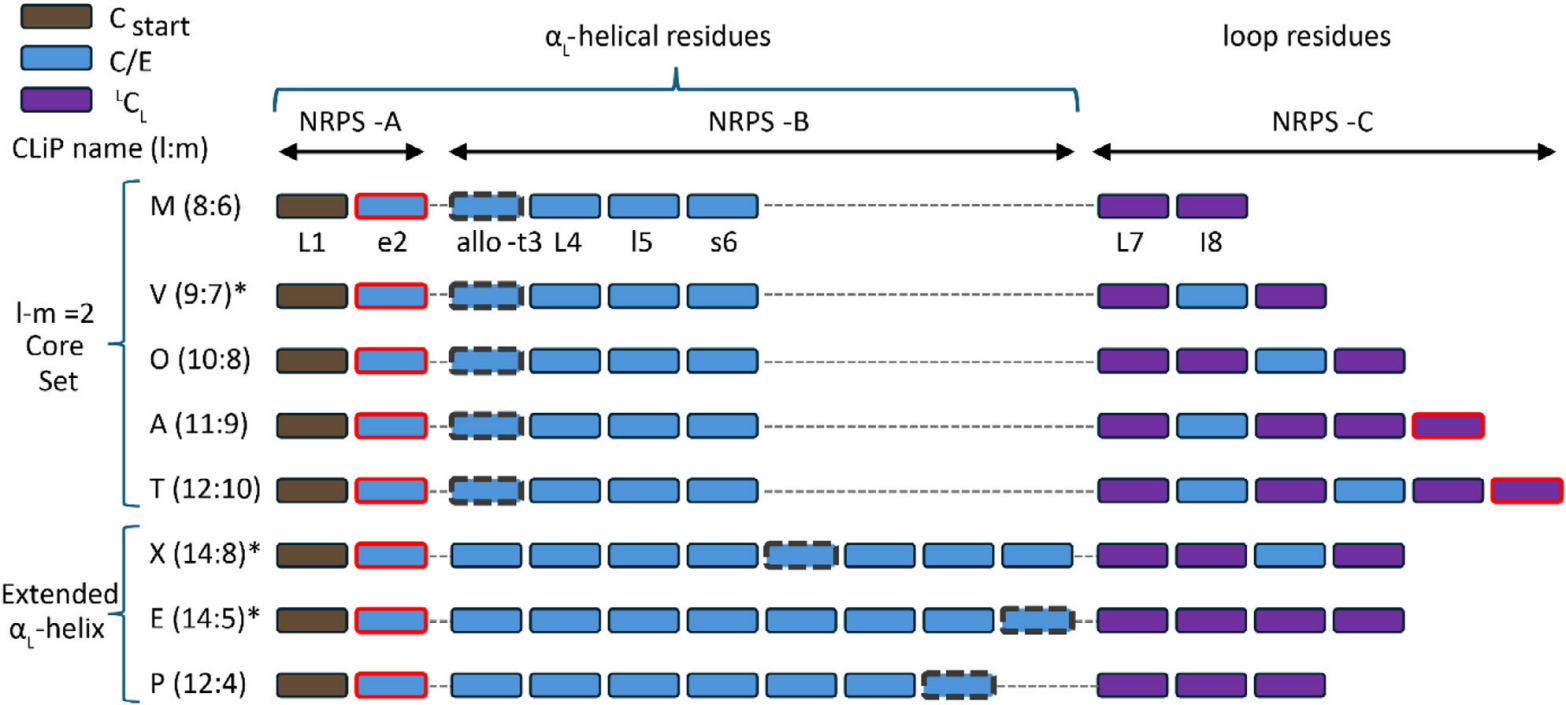
The modular distribution of the NRPS systems of Acidilin CLiP producers (Table 1) investigated in this work.^[^ ^18^, ^31^, ^46^, ^49^, ^50^, ^71^, ^72^, ^73^, ^74^ ^]^ The names of the produced CLiPs are abbreviated as follows: M, MDN‐0066; V, viscosin; O, orfamide; A, arthrofactin; T, tanniamide; X, xantholysin; P, putisolvin; E, entolysin. For clarity, the individual domains (i.e. condensation, adenylation, thiolation) of a module are not shown separately. As exemplified by the sequence of MDN‐0066 (small letter for d‐configuration), each module of a particular NRPS assembly line extends the oligopeptide sequence of the CLiP by one amino acid. The modules are colored according to their condensation domain type being C_start_ (brown), dual C/E (blue) or ^L^C_L_ (purple). Following their recruitment, the C‐start module condenses the N‐terminal amino acid to the fatty acid. The modules providing the acidic residue(s) and the latch residue involved in depsi bond formation are indicated with thick frames colored respectively solid red and dashed grey. ^*^Split BGC, where the first gene encoding NRPS‐A is located at another genomic locus with respect to those encoding NRPS‐B and NRPS‐C. In the Orfamide (10:8) family, only the poaeamides are associated with a split BGC organization.^[^ ^16^ ^]^

The translation of BGC into the various NRPS modules from which the constitution and stereochemistry of the resulting CLiP can be derived is well established in *Pseudomonas*.^[^ ^2^, ^16^ ^]^ We investigated whether the organization of the BGC and the corresponding NRPS enzymes is also reflected in the conformation of the resulting CLiPs. For the 8 Acidilins reported here, three genes *A*, *B* and *C* can be distinguished that each encode respective NRPS multidomain enzyme complexes.^[^ ^18^, ^31^, ^46^, ^49^, ^50^, ^71^, ^72^, ^73^, ^74^ ^]^ (**Figure** **6**) The first gene (*A*) always codes for a di‐modular NRPS‐A that incorporates the N‐acylated Leu1 and the acidic residue at position 2 (d‐Glu2 or d‐Asp2), and may occur at another genomic locus. In the core set (*l–m* = 2) NRPS‐B is consistently tetramodular. It first introduces the latch residue and completes the N‐terminal stapled‐helix. Gene *C* encodes variable NRPS‐C module numbers, parallelling the increase in residues constituting the loop in the macrocycle. The relationship between the specific gene and the α_L_‐helix (NRPS‐A+NRPS‐B), respectively the loop (NRPS‐C) remains consistent in case of the longer CLiPs (*l–m* > 2), xantholysin, putisolvin and entolysin. Indeed, extension of the helix toward the N‐terminus invariably results from insertion of additional modules prior to the one introducing the latch in the NRPS‐B complex, likely caused by duplication and/or reshuffling at the gene level.^[^ ^16^ ^]^ When *m* < 6, this ‘latch module’ becomes the final module of NRPS‐B. The switch from stapled helix to catch‐pole motif thus coincides with NRPS‐C being solely responsible to provide the residues that make up the macrocycle. The distribution of condensation domains over the genes is also notable, as ^L^C_L_ domains only appear in NRPS‐C, while NRPS‐A and NRPS‐B only feature combined condensation/epimerization C/E domains that can epimerize the preceding residue. Therefore, the α_L_‐helix of Acidilins is predicted to consist purely of d‐amino acids, which is indeed the case for arthrofactin (11:9). For most other CLiPs, however, at least one l‐Leu is introduced into the α_L_‐helix due to the absent epimerization activity of the associated C/E domain. Beyond the helix, then, l‐residues in the loop are always incorporated by ^L^C_L_ domains of NRPS‐C rather than inactive C/E domains. If this observation is maintained, it can provide additional assistance in resolving configurational assignments of Acidilin structures. We can conclude that the modular distribution of the NRPS assembly line of *Pseudomonas* defines the secondary structural elements of CLiPs of the Acidilin cluster. Therefore, analysis of the lipopeptide BGCs should allow direct predictions regarding the 3D structure of the produced metabolite.

### 2.8 Higher‐Level Structural Classification of *Pseudomonas* CLiPs

**Figure 7:**
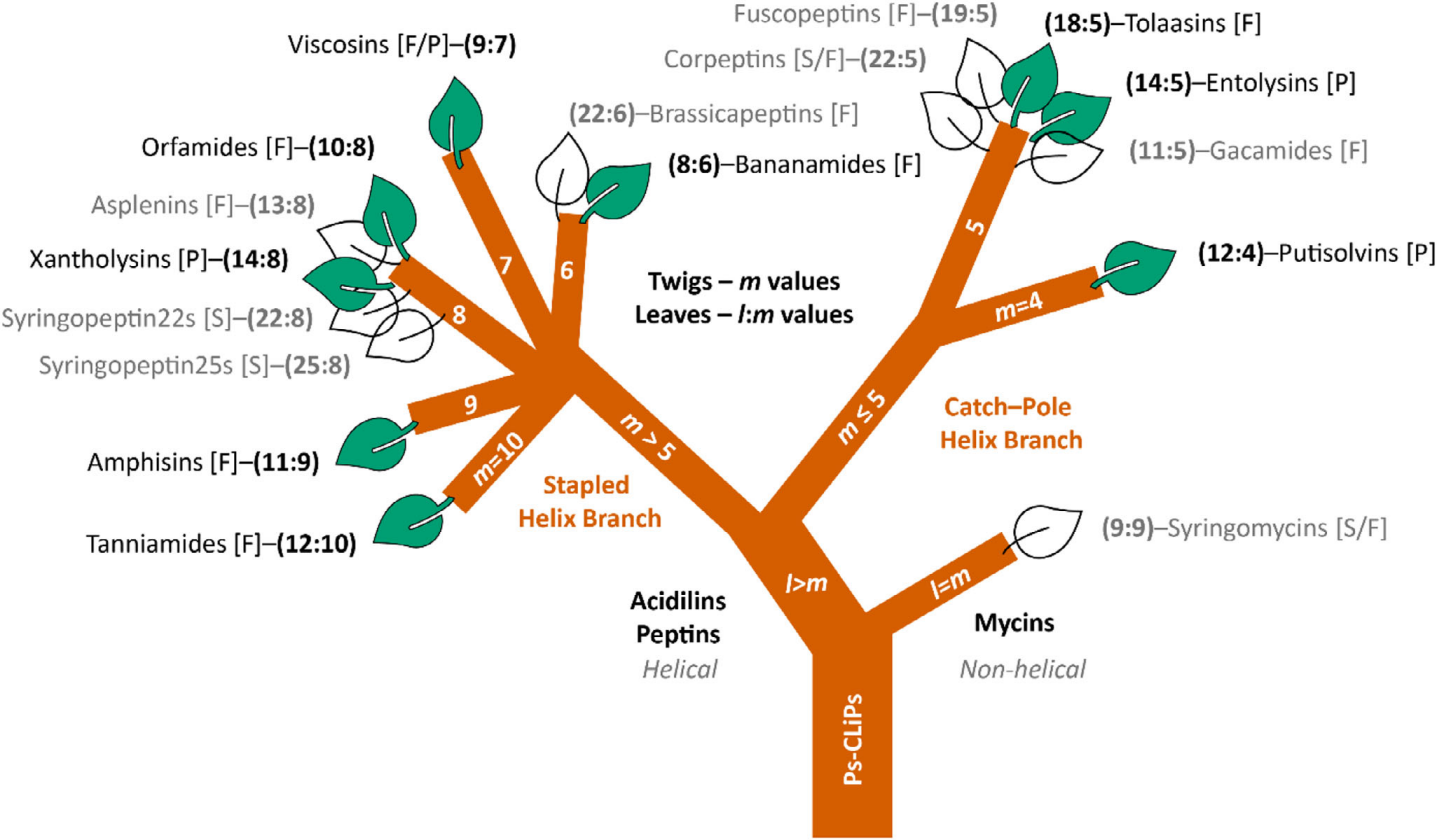
The motif tree of *Pseudomonas* CLiP (Ps‐CLiP) conformations. The 17 CLiP families based on oligopeptide sequence can be organized into a motif tree with the main trunk of *l* > *m* (Acidilins + Peptins) CLiPs and the offshoot of *l* = *m* CLiPs (Mycins). The *l* > *m* trunk can be split into two major branches defined by the macrocycle size: if *m*>5, stapled‐helix branch, if *m* = 5 or 4, catch‐pole branch. The different CLiP conformations according to *l*:*m*‐values are represented by individual leaves attached to the twig of a particular *m*‐value, thus, CLiPs of identical macrocycle sizes correspond to the same twig. Green leaves represent *l*:*m* CLiP families with determined conformation at the water:lipid interface, while empty leaves (CLiP families in grey fonts) represent unknown folded conformations. For each CLiP family, the taxonomic clade(s) of its producer(s) are indicated in squared brackets (F: Fluorescens; P: Putida; S: Syringae). This illustration does not aim to reflect evolutionary or phylogenetic relatedness amongst the different CLiP families.

The results above indicate that Acidilins can adopt two distinct α_L_‐helical motifs depending on the number of macrocycle residues, each defining two major structural branches. When *m ≤*5 the α_L_‐helix of length *l*–*m* is crowned by a macrocycle that adopts a closed loop, giving rise to a catch‐pole motif. When *m* > 5, the α_L_‐helix proceeds into the macrocycle for almost a full turn, before engaging into a closing loop, producing a stapled α_L_‐helix motif. Accordingly, we introduce a ‘tree representation’ in which *Pseudomonas* CLiPs are classified by the structural motif they adopt, as illustrated in **Figure** **7**, with each motif type (stapled vs catch‐pole helix) defining a separate branch and *m* = 5 defining the branching point. Both α_L_‐helical motif branches split up further in various ‘twigs’ depending on the exact value of *m*. Within a branch, the different twigs denote CLiP families adopting the same α_L_‐helical motif type but exhibiting distinct macrocycle conformations beyond the helix as dictated by the specific *m*‐value. The leaves on a particular *m*‐twig indicate CLiP families with the same *m*‐value but different overall length *l*, filled leaves representing CLiPs where the 3D structure is now available. As demonstrated through the orfamide/xantholysin and pentorfamide/entolysin comparisons, similar macrocycle conformations are adopted by leaves on the same twig, provided that the stereochemistry is maintained.

The recently described Acidilin families represented by gacamide A (11:5)^[^ ^75^ ^]^ and asplenin (13:8)^[^ ^76^ ^]^ remain to be solved in order to complete the 3D structure portrait of the Acidilin cluster and currently are represented by blank leaves. Based on their sequences (Figure S1, Supporting Information) and *m*‐values, Gacamides (11:5) are expected to adopt a catch‐pole motif similar to Entolysins (14:5), while Asplenins (13:8) would resemble the stapled helix motif of Xantholysins (14:8), each presenting an α_L_‐helix shortened by three, and respectively, one residue. These predictions are in line with the modular distributions of the NRPS enzymes, with NRPS‐A and NRPS‐B together encoding for 7 modules in gacamide (vs. 10 for entolysin) and 9 modules for asplenin (vs 10 for xantholysin), both genes together defining the number of residues incorporated in their respective α_L_‐helices.

We next considered if our classification could also encompass the Peptins and Mycins which, in addition to the differences in charge, feature distinct BGC architectures than the Acidilins and include several non‐proteinogenic amino acids in their oligopeptide sequences which are not observed in Acidilins (vide infra). Few conformations have been described for *Pseudomonas* CLiPs belonging to these clusters. The only example where coordinates are available is for tolaasin I (18:5)^[^ ^77^ ^]^ from the eponymous CLiP family that can be considered a member of the Peptin cluster, featuring two positive charges at its C‐terminus. Produced by *Pseudomonas tolaasii*, it is best known for causing the characteristic brown blotches on mushroom caps, in addition to other effects including phytotoxicity to tobacco leaves.^[^ ^13^, ^14^ ^]^ Its conformation, solved in micellar SDS solution by Jourdan *et al.*, features a 13 residue long α_L_‐helix topped by the ‘boat‐like’ macrocycle protruding from the main helical axis.^[^ ^78^ ^]^ The macrocycle of tolaasin adopts the same ≈140° angle with respect to the main α_L_‐helix axis as observed for entolysin (14:5) and synthetic pentorfamide (10:5). (Figure S13, Supporting Information) Similarly to pentorfamide, tolaasin features a D/L configurational switch at the penultimate C‐terminal position compared to entolysin. Again, this leads to several localized differences in macrocycle conformation between the two natural *Pseudomonas* CLiPs, however, when tolaasin and pentorfamide are compared visually, we find that their macrocycles show excellent superimposition (Figure S20, Supporting Information) with the backbone phi, psi angles showing agreement to within a few degrees for all residues preceding the depsi‐bonding one (Table S29, Supporting Information). While the authors likened the conformation of the tolaasin molecule to a ‘golf‐club’, we preferred to resort to the ‘catch‐pole’ denomination for the conformational motif type, as this also allows to cover the Putisolvins (12:4) which lack similarity to a golf club due to the quite distinct macrocycle bend angle (≈45°). Like for the Acidilins, the overall amphipathic sequence of tolaasin I translates into well separated hydrophobic and hydrophilic surfaces,^[^ ^78^ ^]^ supporting its pore forming activity in biological membranes.^[^ ^13^, ^14^, ^15^ ^]^ Thus, in spite of distinct differences in amino acid composition compared to entolysin, including a d‐Pro2, two (*Z*)‐dehydroaminobutyric (*Z*‐Dhb) acid residues in the exocyclic helix, as well as homoserine (Hse), d‐diaminobutyric acid (Dba) and Lys residues in the macrocycle loop, these are found to decorate the same *Pseudomonas* CLiP conformational motif type.

Other Peptin CLiP families such as the Fuscopeptin (19:5), Corpeptin (22:5), Brassicapeptin (22:6), Syringopeptin‐22 (22:8) and Syringopeptin‐25 (25:8) families distinguish themselves from the Tolaasins by the completely hydrophobic nature of the exocyclic sequence abundant in d‐Ala and d‐Val residues, suggesting an alternative membrane interaction mode. (For representative sequences see Figure S1 (Supporting Information). Broader overview of the Peptins and Mycins can be found in reviews [3] and [5].) Of these, fuscopeptin B (19:5),^[^ ^79^ ^]^ and syringopeptin‐25A (25:8)^[^ ^80^ ^]^ were the subjects of early solution state NMR and circular dichroism (CD) studies in water:trifluoroethanol (TFE) solvent mixtures considered to mimic a water:lipid interface environment. While unstructured in pure water, for these peptins increasing α‐helical content was observed upon increasing TFE, a feature typical for amphipathic peptides. Based on these observations, we propose that Peptins will also structure themselves at the water:lipid interface, whereby the long exocyclic peptide sequences are expected to fold into fully hydrophobic α_L_‐helices. Since Fuscopeptins (19:5) and Corpeptins (22:5) display the same macrocycle backbone stereochemistry as the Tolaasins, they are expected to adopt a tolaasin‐like catch‐pole motif with more extended, hydrophobic α_L_‐helices. Syringopeptin‐22A and syringopeptin‐25A, which represent the largest known CLiPs in terms of residue number (*l*), are identical to Orfamides (10:8) and Xantholysins (14:8) regarding macrocycle size and stereochemistry albeit hydrophilic residues only occur in the macrocycle. Consequently, Syringopeptins likely adopt a stapled helix motif with macrocycle conformation similar to orfamide/xantholysin but more extended and fully hydrophobic α_L_‐helices, in the presence of a suitable water:lipid interface.

As the large majority of *Pseudomonas* CLiPs belong to the Acidilin (10 families) or Peptin (6 families) clusters, the scheme in Figure 7 provides a higher order classification depending on the α_L_‐helical motif adopted as a result of the *m*‐value based on the experimental or predicted 3D structures of CLiP family representatives. The stapled helix branch is more populated (10/17 (*l*:*m*) CLiP families) than the catch‐pole helix branch (6/17). Also, there is no correlation between either of these α_L_‐helical motif types and the affiliation of the respective *Pseudomonas* producers with the Fluorescens, Putida or Syringae phylogenetic clades.

Finally, the Mycins (9:9) most likely constitute a distinct case within the portfolio of *Pseudomonas* CLiPs regarding their conformation as their primary sequences are dominated by hydrophilic residues while the macrocycle encompasses the entire peptide sequence, i.e. *l* = *m*. Unlike the Acidilins and Peptins where *l* > *m*, these fully cyclic peptides are not expected to adopt either α‐helical motif. Previous studies^[^ ^81^ ^]^ used limited nOe data obtained in water:SDS solution to reveal a ‘saddle‐like’ conformation for syringomycin E, supporting this view. Thus, Mycins (9:9) constitute a separate *l* = *m* ‘offshoot’ for the *Pseudomonas* CLiP motif tree, whereas all other families satisfy *l* > *m* and constitute the main trunk of the tree.

## 3 Conclusion

*Pseudomonas* CLiPs feature large structural diversity in their chemical constitution but appear to display conformations of limited variety, providing the basis for a 3D structure based, higher‐order classification of this class of specialized metabolites. Elucidating the conformation of 8 antimicrobial, non‐phytotoxic CLiPs and that of a synthetic analogue (pentorfamide) representing the Acidilin cluster, together with previous investigations on CLiPs representing the Peptins, we find that CLiPs where the oligopeptide is partially exocyclic (16 out of the 17 known *Pseudomonas* CLiP families) consistently display left‐handed α‐helical conformation, giving rise to only two possible structural motif types defined by the residue number in the macrocycle (*m*): a stapled‐helix (*m* > 5) or a catch‐pole helix (*m* ≤ 5). In these structures well separated hydrophobic and hydrophilic faces emerge which substantiates the membrane interaction capacity of CLiPs. This includes the acyl chain which is unstructured but consistently located along the hydrophobic face. CLiP families sharing the same *m*‐value display alike macrocycle conformations provided the D/L configurations are maintained in the sequence, with differences in the number of exocyclic residues (*l*) modulating the length of the α_L_‐helix. By covering all known *m*‐values (4–10) amongst *Pseudomonas* CLiPs we provide an array of structural templates available from the RCSB Protein Data Bank (https://www.rcsb.org/) to provide essential starting points for structure‐function relationship studies,^[^ ^70^ ^]^ support the interpretation of biophysical and in silico studies involving interactions with (model) membranes and help uncover phylogenetic patterns relating these to biosynthetic gene cluster organization. Indeed, while reliable structure prediction methods based on machine learning algorithms are now available for peptides and proteins, the absence in the training set of peptide structures containing d‐ and other non‐proteinogenic amino acids as well as one or more cycles, such as seen in CLiPs or lantibiotics^[^ ^82^, ^83^ ^]^ for instance, precludes their application.^[^ ^84^, ^85^ ^]^

The 8 available CLiP 3D structures and higher‐order classification scheme (Figure 7) can now be used as input for homology modelling of existing or newly identified members of these families. More generally, we propose that any *Pseudomonas* CLiP where *l* > *m*, can be classified to either left‐handed α‐helical structural motif based on the *m*‐value as derived from chemical and/or bioinformatic analysis. CLiPs from families with *m*‐value and macrocycle sequence configuration identical to an existing 3D structure, but differing in *l*‐value can be built using the existing structure as a template, with adequate extension or truncation of the α_L_‐helix depending on *l*. As new CLiP families are identified, the tree representation may grow more leaves, twigs or branches, and potentially novel offshoots, which will contribute to fine tune the higher order classification scheme. Furthermore, the correlations found between the conformation of Acidilin‐type CLiPs and the modular distribution of their biosynthetic gene cluster allows, for the first time, guidance for conformational predictions of cluster members based on the genetic make‐up of the *Pseudomonas* producer. This unlocks a crucial step toward the genomic engineering of such metabolites taking into account their 3D structure. It warrants investigating correlations between the conformational motif type and the bioactivity profile and/or mode of action via standardized benchmark assays toward the future. In this respect, a comparison between related but motif‐divergent natural CLiPs (xantholysin versus entolysin) can be informative, while the systematic investigation of biophysical properties and biological activity of orfamide A and the synthetic pentorfamide analogue, promises to reveal motif‐related modulations in membrane interaction properties and bioactivities.

In addition, our results can inspire the exploration of molecular design strategies that could counteract the enzymatic hydrolysis of the depsi bond which is emerging as a detoxification or inactivation strategy in microbial communities. Previous studies have shown that both catch‐pole (tolaasin) and stapled‐helix (pseudodesmin, orfamide A) CLiPs can be linearized by microbial enzymes released by competing organisms.^[^ ^86^, ^87^ ^]^ Indeed, the availability of defined structural templates now enables the design and synthesis of analogues that retain the characteristic motif while resisting enzymatic cleavage, potentially improving their stability and usability in soil and rhizosphere environments. Beyond CLiPs, the Ser/Thr side‐chain to C‐terminus stapling giving rise to the stapled‐helix motif has the potential to inspire a novel class of side‐chain‐to‐backbone stapled peptides or serve as a template for grafting protein‐derived helical segments. Furthermore, these conformationally stabilized cyclic peptides may define novel starting points for the rational design of simplified CLiP‐inspired scaffolds, including analogues that omit costly D‐amino acids while preserving key conformational features. More generally, CLiPs offer novel templates for the development of bioactive molecules with novel, tunable properties. For instance, their amphipathic, membrane‐interacting α‐helical architectures, may provide a valuable blueprint for designing new cell‐penetrating peptides, capable of delivering small‐molecule cargos, or possibly even oligonucleotides or small proteins, thereby expanding their utility well beyond natural antimicrobial functions.

## Conflict of Interest

The authors declare no conflict of interest.

## Author Contributions

B.K. produced and analyzed the majority of the NMR and computational data and obtained the presented conformations of the 8 *Pseudomonas* CLiPs and pentorfamide. The work was initiated and supervised by J.C.M.D.P. assisted in the structure determination of tanniamide and contributed to overall conformational analysis, visual representations and data interpretation. V.D.R assisted in the structure determination of MDN‐0066 and initial entolysin structure determination. The synthesis of pentorfamide was performed by M.V. with the assistance of P.M. under the supervision of A.M. Contexts concerning biosynthetic gene cluster analysis and CLiP biological functions were contributed by R.D.M and M.H following multiple discussions. They also provided the various *Pseudomonas* strains for natural CLiP production. N.G. coordinated most of this eight‐year effort, in particular with regards to NMR sample preparation, the determination of several CLiP structures and various steps along the workflow, as well as contributing to scientific data interpretation and discussions. The manuscript was written by B. K. and J. C. M. and refined by all authors.

## Supporting information



Supporting Information

## Data Availability

The NMR structures of MDN‐0066, viscosin, orfamide A, arthrofactin A, tanniamide A, xantholysin A, putisolvin I and entolysin A in micellar DPC solution can be obtained from the RCSB Protein Data Bank (https://www.rcsb.org/). ^1^H and ^13^C Chemical shifts are available in the BMRB (https://bmrb.io/) database (for PDB and BMRB accession codes see Table 1) as well as the Supporting Information of the manuscript which also contains detailed Experimental and Methods Sections including information on NMR sample preparation (Section S2, Supporting Information), NMR structure calculation protocol (SI Sections 3), the conformational analysis of the investigated CLiPs (Sections S4 and S5, Supporting Information) and additional references.^[88–106]^ For all natural CLiPs as well as the synthetic pentorfamide the crude Bruker spectra, input files for the CNS structure calculations, the subsequent AMBER MD simulations in water:DPC environment as well as the produced 100 ns trajectories are available from the Zenodo data repository. (https://doi.org/10.5281/zenodo.15024231) A list of known CLiPs produced by *Pseudomonas* collected from literature can be made available upon request.
