# Supplementary material for: Higher‐Level Structural Classification of Pseudomonas Cyclic Lipopeptides through Their Bioactive Conformation
Source: Adv Sci (Weinh). 2025 Dec 14;13(12):e20365. doi: 10.1002/advs.202520365 (PMC12948218; doi:10.1002/advs.202520365)
Supplement: Supplementary file 1 — Supporting Information [file ADVS-13-e20365-s001.docx]

Supporting Information

**Higher-Level Structural Classification of Pseudomonas Cyclic Lipopeptides through Their Bioactive Conformation**

Benjámin Kovács, Durga Prasad, Vic De Roo, Matthias Vanheede, Penthip Muangkaew, Annemieke Madder, Monica Höfte, René De Mot, Niels Geudens and José C. Martins*

Table of Contents

[1. Overview of *Pseudomonas* CLiP families and selection of representative CLiPs for structure elucidation 3](#_Toc215150539)

[2. Experimental methods 5](#_Toc215150540)

[2.1. Production, isolation and characterization of *Pseudomonas* CLiPs 5](#_Toc215150541)

[2.2. Synthesis of pentorfamide (10:5) 5](#_Toc215150542)

[2.3. NMR sample preparation, data acquisition and resonance assignment 8](#_Toc215150543)

[2.4. ^1^H and ^13^C chemical shifts of 8 natural CLiPs and pentorfamide 10](#_Toc215150544)

[2.5. Characterisation of CLiP incorporation into DPC micelles 25](#_Toc215150545)

[3. NMR structure calculation protocol 26](#_Toc215150546)

[3.1. Initial CNS structure generation 26](#_Toc215150547)

[3.2. Structure refinement via molecular dynamics with explicit DPC micelle 26](#_Toc215150548)

[3.3. Molecular dynamics trajectory analysis 27](#_Toc215150549)

[4. Conformational analyses and comparisons of 8 *Pseudomonas* CLiPs 29](#_Toc215150550)

[4.1. The backbone dynamics of the natural CLiP structures in membrane-bound state 29](#_Toc215150551)

[4.2. PROCHECK analysis of the CNS vs MD refined CLiP conformations 37](#_Toc215150552)

[4.3. Determination of the angle positioning the macrocycle with respect to catch-pole helices 45](#_Toc215150553)

[4.4. Surface property calculations and acyl chain reorientations 45](#_Toc215150554)

[4.5. Solvent exposure graphs 49](#_Toc215150555)

[4.6. Backbone alignments of Viscosins (9:7) and Amphisins (11:9) 51](#_Toc215150556)

[5. Pentorfamide conformation analysis 53](#_Toc215150557)

[References 59](#_Toc215150558)

# Overview of *Pseudomonas* CLiP families and selection of representative CLiPs for structure elucidation

The 17 currently known CLiP families according to *l*:*m* classification are enlisted in Figure S1 along with representative sequences of which 8 CLiPs were selected for structure elucidation. The rationale in selecting these 8 CLiPs results from a number of initial considerations and subsequent developments in the course of this work. Originally, we had focused on the solution structures of pseudodesmin A and viscosinamide A, two atypical Acidilins from the (9:7) Viscosin family which lack an acidic residue due to a Glu to Gln substitution^[30]^. As all other CLiPs within the 8 investigated families feature an acidic residue at position 2 in the sequence, we considered these two CLiPs less typical and selected viscosin, the first ever reported *Pseudomonas* CLiP, as representative CLiP for the (9:7) family. Starting from viscosin and focusing only on the oligopeptide sequence, we noted from the (*l*:*m*) tag that 4 families have a similar fatty-acid N-capped exocyclic dipeptide (*l*–*m*=2) in common, which is followed by a macrocycle that increases in size from 6 to 9. These families and their representative CLiPs are shown in the core of Figure 1: (8:6) Bananamides with MDN-0066_,_ (9:7) Viscosins with the eponymous viscosin, (10:8) Orfamides with orfamide A and (11:9) Amphisins where arthrofactin A was selected, all extracted from *Pseudomonas* strains as indicated in Table 1 in the main text. Later on, this core set of CLiPs was expanded to include (12:10) tanniamide A from the Tanniamide family, currently the largest oligopeptide satisfying the *l–m*=2 condition. Using the d-*allo*-Thr3 residue involved in macrocyclization as the anchor point, alignment of the five sequences reveals strong conservation of amphipathicity within the N-terminal hexapeptide. This region features conservative amino acid substitutions (Asp/Glu; Leu/Val/Ile) and occasional d-/l-configurational switches at Leu1 and Leu5. In contrast, the C-terminal segment displays markedly more variability, both in sequence and stereochemistry, with the amphipathic alignment no longer upholding. The alignment suggests a common conformation for the N-terminal ‘core’ followed by family specific variations in macrocycle conformation associated with the more variable C-terminal extension, setting the stage for the structure determination effort.

Putisolvin I and entolysin A were chosen to represent their eponymous (12:4) and (14:5) families, respectively. Both CLiPs maintain good alignment with the N-terminal hexapeptide core sequences, but feature a smaller macrocycle, now cyclized via a d-Ser side-chain. They also have a more extended exocyclic octa- and nonapeptide segment respectively (top, Figure 1). By investigating Acidilin structures with *m* from 4 to 10 we cover all known macrocycle sizes amongst Acidilins as well as *Pseudomonas* CLiPs in general. Finally, xantholysin A representing (14:8) Xantholysins was included as a second *m*=8 family in addition to the Orfamides, to investigate whether macrocycle conformation is maintained across family boundaries, and assess the possible impact of the longer, N-terminal oligopeptide extension (*l*=14 vs *l*=10). The obstacle posed by the partial, absent or unreliable knowledge on stereochemistry for six of these CLiPs was overcome by the development of an NMR based fingerprinting method to decode the stereochemistry of CLiPs, as described elsewhere.^[19]^


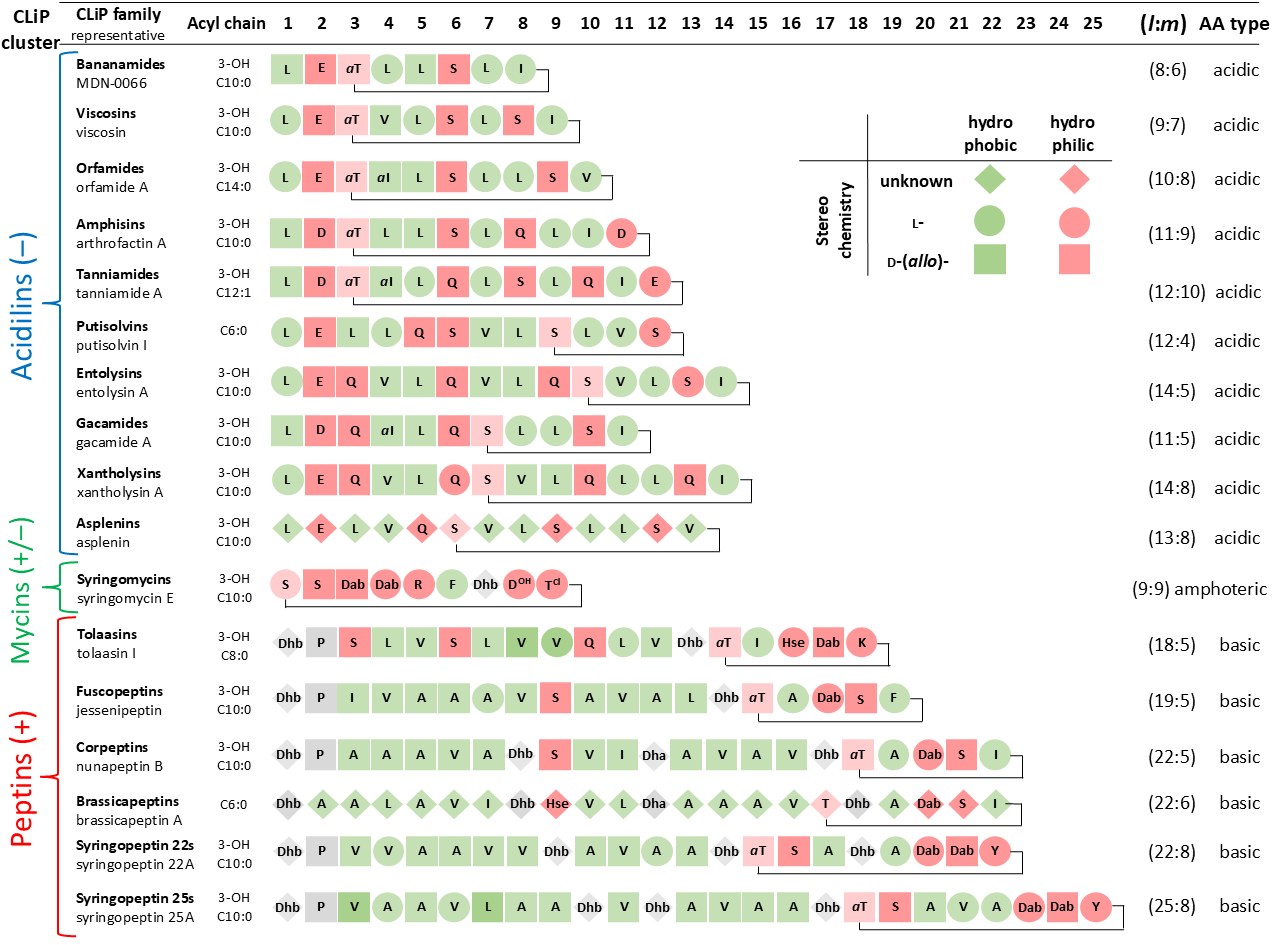


Figure S1. The 17 known *Pseudomonas* cyclic lipodepsipeptide (CLiP) families (first letter capitalized) based on *l*:*m* classification and representative sequences (first letter small, except for MDN-0066). Residues are shown as follows: circle: l-amino acid; square: d-amino acid; diamond: unknown/non-chiral Cα stereochemistry; green: hydrophobic side-chain; red: hydrophilic side-chain. Proteinogenic amino acids and their d-enantiomers are represented by 1-letter codes, otherwise: ‘D^OH^’: 3-hydroxy-(*S*)-l-Asp; ‘T^Cl^’: l-chloro-Thr; ‘*a*T’: d-*allo*-threonine; ‘*a*I’: d-*allo*-isoleucine; ‘Dhb’: (*Z*)-dehydrobutyric acid; ‘Dha’: dehydroalanine; ‘Dab’: 2,4-diaminobutyric acid; ‘Hse’: homoserine. The length and saturation level of the N-terminal acyl chains together with the presence of a hydroxyl group attached to the (*R*-configured) β-carbon atom are also indicated. ‘C6:0’ = hexanoic acid; ‘3-OH C10:0’ = (*R*)-3-hydroxydecanoic acid; ‘3-OH C14:0’ = (*R*)-3-hydroxytetradecanoic acid; ‘3-OH C12:1’ = (*R*)-3-hydroxydodec-(*Z*)-5-enoic acid. The corresponding cluster (Acidilins, Peptins, Mycins) and the presence of ionizable side-chains at physiological pH are also indicated for each CLiP group/sequence.

# 2. Experimental methods

## **2.1. Production, isolation and characterization of *Pseudomonas* CLiPs**

The biosynthetic production, extraction and purification of the *Pseudomonas* CLiPs MDN-0066, viscosin, orfamide A, arthrofactin A, tanniamide A, putisolvin I, entolysin A and xantholysin A were performed according to protocols as described in our earlier works.^[18,19,46,30,50]^ Briefly, the producing *Pseudomonas* strains (Table 1) were grown for 24 h (28°C, 150 rpm) in King’s broth (KB, Difco Laboratories, Sparks, MD, USA) except for MDN-0066 producing *P. azadiae* SWRI103 where minimal salt (M9) medium was used to improve production levels. Following bacterial growth, the broth was centrifuged to separate the cells and the medium (10 min, 5 °C, 8000 rpm). As both can contain CLiPs, the supernatant was extracted through acidification at low temperature (5^o^C) using a 2 M HCl solution at pH 2–3, while the cell pellet was resuspended in ethyl acetate and subjected to multiple freeze-thaw cycles using liquid nitrogen, prior to extraction as described for the supernatant. The crude medium and cell extracts were dissolved in methanol prior to HPLC on a Prostar HPLC (Varian) equipped with a Kinetex C18 semi-preparative column running at 30°C. Validation of the chemical structures of all CLiPs was performed using a combination of LC-MS data and 2D NMR experiments recorded at 700 MHz in organic solvents (AcN-*d*3 or DMF-*d*7) including TOCSY, NOESY, ^1^H-^13^C HSQC and ^1^H-^13^C HMBC.

## **2.2. Synthesis of pentorfamide (10:5)**

The general approach for the synthesis of pentorfamide is based on the previously developed solid-phase peptide synthesis approach for pseudodesmin A^[70]^ with some minor changes. The synthetic route for the synthesis of pentorfamide is shown in SI Scheme S1.

*Loading of the 2-CTC resin via side-chain anchoring of Fmoc-d-Ser(OH)-Oallyl*

Fmoc-d-Ser(OH)-Oallyl (2.2 g, 5.99 mmol, 1.5 eq.) was dissolved in dry DMF (10 mL) in a round bottom flask under an inert (Ar) atmosphere, followed by the addition of dry pyridine (0.967 mL, 11.98 mmol, 3 eq.). The 2-CTC resin (2.495 g, 3.99 mmol, 1 eq.) was added to a double-walled glass reactor under argon atmosphere. The reaction mixture was added to this reactor and was connected to a thermostat with temperature set to 60°C. The reactor was shaken for 24 hours in a Selecta Vibromatic Shaker. Next, all excess reagents were filtered and the resin was washed with dry DCM. The unreacted functionalities were capped by adding a solution of dry DCM:dry MeOH:dry DIPEA (17:2:1) for 2x10 minutes. Afterwards, the resin beads were thoroughly washed with DMF (5x), DCM (5x), MeOH (5x) and Et_2_O (5x). The resin was dried at the oil pump overnight. The loading was determined by UV monitoring the absorbance of the dibenzofulvene-piperidine adduct formed after Fmoc-deprotection (at 300 nm). A loading of 0.520 mmol^.^g^–1^ was obtained.

**Scheme S1.** Synthetic route towards pentorfamide. Reagents and conditions: **(a)** 40% piperidine in DMF (v/v), microwave 45°C, 2 x 4 min; **(b)** Fmoc-AA-OH (5 eq.), Oxyma Pure (5 eq.), DIC (5 eq.), DIPEA (0.25 eq.), DMF/NMP, microwave 65°C, 2 x 10 min; **(c)** alloc-l-Val-OH (10 eq.), DIC (11 eq.), DMAP (0.2 eq.), pyridine (5 eq.), DMF; **(d)** phenylsilane (60 eq.), Pd(PPh_3_)_4_ (0.25 eq.), CH_2_Cl_2_, 2 x 1 hour; **(e)** Oxyma Pure (5 eq.), DIC (5 eq.), DIPEA (0.25 eq.), DMF/NMP, microwave 75°C, 2 x 15 min; **(f)** TFA/TIS/H_2_O (95/2.5/2.5).

*Solid-phase peptide synthesis*

Semi-automated solid-phase peptide synthesis (SPPS) towards pentorfamide was performed on a Biotage Initiator+ Microwave Synthesizer. The peptide synthesis made use of the Fmoc/tBu protecting strategy with DIC/Oxyma Pure as coupling reagents. Fmoc-deprotection was carried out by adding a 40% piperidine solution in DMF (v/v) for 4 min at 45°C under microwave irradiation. This step was repeated to ensure complete deprotection. The coupling step was carried out on-resin by dissolving the Fmoc-protected amino acid (5 eq.) in DMF, followed by the addition of Oxyma Pure (5 eq.) in DMF and DIC (5 eq.)/DIPEA (0.25 eq.) in NMP for 10 minutes at 65°C. The coupling step was repeated to ensure full incorporation of the amino acid in the peptide sequence. In a C🡪N fashion, Fmoc-l-Leu-OH, Fmoc-l-Leu-OH, Fmoc-d-Ser(OH)-OH, Fmoc-d-Leu-OH, Fmoc-d-*allo*-Ile-OH, Fmoc-d-*allo*-Thr(OtBu)-OH, Fmoc-d-Glu(OtBu)-OH and Fmoc-l-Leu-OH were coupled onto the modified Fmoc-d-Ser-Oallyl-2-CTC resin (192 mg, 100 µmol).

For the esterification reaction, alloc-l-Val-OH (201 mg, 1.0 mmol, 10 eq.) was dissolved in 1.5 mL dry DMF in a dry pear-shaped flask under Ar atmosphere and placed in an ice bath. DIC (0.172 mL, 1.1 mmol, 11 eq.) was added to the reaction mixture and the solution was stirred for 30 minutes. Afterwards, DMAP (2.44 mg, 0.02 mmol, 0.2 eq.) and pyridine (40.3 µL, 0.5 mmol, 5 eq.) were dissolved in 500 µL dry DMF and were also added to the reaction mixture. The preactivated solution was added to the peptidyl resin and they were shaken for 24 hours at room temperature. Afterwards, the resin was washed with DMF (5x), DCM (5x), MeOH (5x) and Et_2_O (5x).

Next, the allyl and alloc protecting groups were selectively removed. Therefore, the resin was swollen in dry DCM under Ar atmosphere. A premixed solution of phenylsilane (0.740 mL, 6 mmol, 60 eq.) and Pd(PPh_3_)_4_ (28.89 mg, 0.025 mmol, 0.25 eq.) dissolved in dry DCM (2 mL) was added to the peptidyl resin under Ar atmosphere. The reactor was shielded from light and was shaken for 1 hour at room temperature. Consequently, the solution was filtered and the beads were washed with dry DCM (3x). This deprotection reaction was repeated to ensure complete removal of both the allyl and alloc groups.

The on-resin cyclisation step was performed by adding Oxyma Pure (5 eq.) in DMF and DIC (5 eq.) in the presence of DIPEA (0.25 eq) in NMP to the peptidyl resin. This reaction was carried out for 15 minutes at 75°C on the Biotage Initiator+ Microwave Synthesizer.

In the final step, the lipid tail ((R)-3-hydroxytetradecanoic acid) was incorporated in the sequence. After Fmoc-deprotection of the last amino acid (l-Leu) in the sequence, the lipid tail was inserted via a peptide coupling reaction, as described above. Afterwards, the peptide was cleaved from the 2-CTC resin with TFA/TIS/H_2_O (95/2.5/2.5). After precipitation with MTBE, the peptide was purified by semi-preparative reversed-phase (RP) HPLC using elution by a linear gradient over 25 min of H_2_O (containing 0.1% TFA) and CH_3_CN from 50:50 to 0:100 using a Phenomenex AXIA packed Luna C18 column (250 x 21.2 mm, 5 µm particle size at 35°C). The combined product fractions were lyophilized and the pure peptide was analyzed by LC-MS.

LC-MS analysis

Exact expected mass for pentorfamide C_64_H_114_N_10_O_17_ = 1294.84 Da. The pure compound (SI Figure S2) eluded with a retention time of 7.67 min under the applied RP-HPLC conditions (see SI Figure S2 legend) with the corresponding mass of 1295.66 Da that belongs to the protonated ([M+H]^+^) state.

NMR analysis

All NMR characterizations for building blocks were performed at 400.13 MHz (^1^H) and 100.613 MHz (^13^C) on a Bruker Avance NEO equipped with a TCI Prodigy cryoprobe. Chemical shift assignments were identical to results published previously. Characterization of the pentorfamide end-product was performed directly in DPC micelles as described in the next section (Table S10).


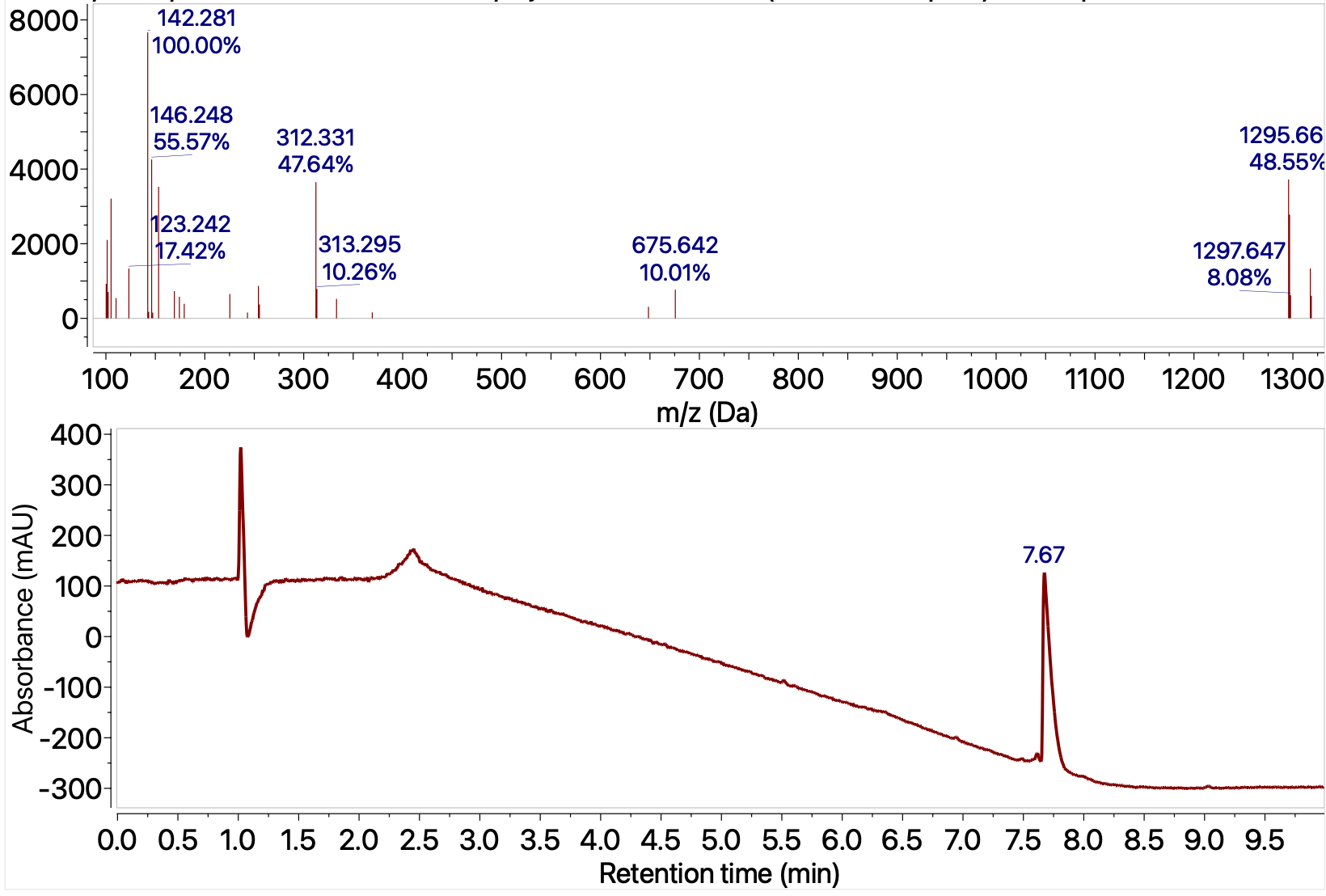


**Figure S2.** RP-HPLC chromatogram of pure pentorfamide (bottom). Kinetex C-18 column with linear gradient over 6 min of 0.1% HCOOH in H_2_O and CH_3_CN from 100:0 to 0:100. Detection at a wavelength of λ=214 nm. The MS spectrum (top) is also included.

## **2.3. NMR sample preparation, data acquisition and resonance assignment**

Sample preparation details specific to each CLiP are collected in Supplementary Table S1. To provide membrane mimicking circumstances, all NMR measurements were performed in a buffered solution of zwitterionic DPC-*d*38 (98% atom D, Cambridge Isotope Laboratories) in 90% H_2_O/D_2_O (v/v) at pH 7.4 using high-precision 5-mm NMR tubes (Norell Inc). A 10 mm Na_2_HPO_4_/NaH_2_PO_4_ buffer was added to solid DPC-*d*38 followed by sonication until complete dissolution of the detergent. Subsequently, 1.0 to 3.3 mg of the purified CLiP powder was dissolved in the micellar phosphate buffer solution well above the CMC reported as 1.35 mm under these conditions.^[88]^ In all cases the DPC to CLiP ratio was at least 50:1, aiming at avoiding multiple CLiPs per micelle, considering that the aggregation number of DPC is 56.^[89]^ The mixture was once again sonicated until the complete dissolution of the CLiP and transferred into the NMR tube. The final concentration of the CLiP and the DPC (in the NMR tube) was determined using the digital ERETIC method^[90]^ as implemented in TopSpin 3.6 software (Bruker). All ERETIC and diffusion NMR measurements used a Bruker Avance III spectrometer operating at a respective ^1^H and ^31^P frequency of 500.13 MHz and 202.4 MHz equipped with a BBI-Z probe.

**Table S1.** Quantity of the solutes, their final concentration and the corresponding peptide to micelle ratio for each sample M: MDN-0066, V: viscosin, O: orfamide, A: arthrofactin, T: tanniamide, PU: putisolvin, EL: entolysin, X: xantholysin and PO: pentorfamide.

| CLiP | M | V | O | A | T | PU | EL | X | PO |
| --- | --- | --- | --- | --- | --- | --- | --- | --- | --- |
| *l:m* | 8:6 | 9:7 | 10:8 | 11:9 | 12:10 | 12:4 | 14:5 | 14:8 | 10:5 |
| $m_{CLiP}$ (mg) | 1.0 | 1.1 | 1.5 | 1.1 | 1.6 | 2.1 | 2.7 | 3.3 | 1.3 |
| $c_{CLiP}$ (mm) | 1.5 | 1.8 | 1.9 | 1.3 | 1.8 | 2.7 | 2.5 | 3.2 | 1.8 |
| $m_{DPC}$ (mg) | 21.8 | 26.1 | 23.2 | 31.3 | 22.4 | 26.8 | 33.3 | 36.2 | 22.5 |
| $c_{DPC}$ (mm) | 104.3 | 124.3 | 111.9 | 142.8 | 102.0 | 127.8 | 150.0 | 162.9 | 102.5 |
| DPC:CLiP | 70 | 69 | 59 | 110 | 56 | 50 | 60 | 51 | 56 |
| CLiP:M*** | 0.8 | 0.8 | 1.0 | 0.5 | 1.0 | 1.2 | 1.1 | 1.1 | 1.0 |

*** *CLiP to micelle (M) ratio assuming 56 molecules per micelle as the aggregation number for DPC.*

The assignment of the CLiP ^1^H and ^13^C resonances in DPC micelles was obtained from measurements performed on a Bruker Avance II spectrometer operating at a respective ^1^H and ^13^C frequency of 700.13 MHz and 176.05 MHz and equipped with a 5 mm Prodigy N2 cryocooled TCI probe. Assignment of the ^1^H and protonated ^13^C nuclei in the CLiP structures to their position in the spectra was achieved at either 298.0 K or 310.0 K via a combined analysis of COSY, NOESY (100 ms), TOCSY with 80 ms DIPSI2 spin lock, and gradient-enhanced multiplicity edited ^1^H-^13^C HSQC spectra, as follows. For the COSY, TOCSY and me-HSQC experiments the interscan relaxation delay was set to 1.0 s, the spectral width was set to 11 ppm in the ^1^H dimension and 90 ppm in the ^13^C dimension (HSQC) with 32 to 48 scans acquired. Generally, 2048 data points were sampled in the direct dimension and 256 data points in the indirect dimension. For 2D processing, the spectra were zero filled to a 2048×2048 real data matrix and multiplied with a squared cosine bell function in both dimensions before Fourier transformation. The NOESY experiments, which were also used for collecting data for the structure calculations were recorded with an increased number of scans (NS=128 or higher), an interscan delay of 1.5 s, and a uniform mixing time of 100 ms established from the analysis of nOe build-up curves. For the NOESY experiments 2048 data points were sampled in the direct dimension and 512 data points in the indirect dimension combining to a total acquisition time of ~30 h per sample. The NOESY correlations were assigned by first identifying the respective spin systems using 2D TOCSY/COSY and ^1^H-^13^C HSQC spectra whereas sequence location was obtained from the analyzing sequential nOe contacts in the corresponding NOESY spectra. The temperature of the measurements was optimized at either 298.0 K or 310.0 K to minimize amide ^1^H resonance overlap. All ^1^H experiments were performed with excitation sculpting^[91]^ to suppress the water signal. Standard pulse sequences as present in the Bruker library were used throughout. The ^1^H and ^13^C dimensions were referenced to the methyl signals of DSS. The assigned ^1^H and ^13^C chemical shifts are provided in SI Tables S2–S10 and deposited in the Biological Magnetic Resonance Data Bank (BMRB). Accession codes are collected in Table 1 in the main manuscript. The raw NMR data were recorded and processed with Bruker’s TopSpin 3.6 software. The original Bruker spectra of each CLiP in micellar DPC solution are available from the Zenodo data repository. (<https://doi.org/10.5281/zenodo.15024231>)

## **2.4. ^1^H and ^13^C chemical shifts of 8 natural CLiPs and pentorfamide**

**Table S2.** The ^1^H and ^13^C chemical shifts of **MDN-0066 (8:6)** in DPC-*d*38 solution at 298.0 K.

| Residue | Group or atom | *δ*_H_ / ppm | *δ*_C_ / ppm |
| --- | --- | --- | --- |
| d-Leu1 | NH | 8.74 | - |
|  | CHα | 3.95 | 54.2 |
|  | CH_2_β | 1.69/1.54 | 39.1 |
|  | CHγ | 1.75 | 24.5 |
|  | CH_3_δ1 | 0.92 | 22.6 |
|  | CH_3_δ2 | 0.85 | 21.2 |
| d-Glu2 | NH | 8.68 | - |
|  | CHα | 3.96 | 55.8 |
|  | CH_2_β | 1.95/1.95 | 26.2 |
|  | CH_2_γ | 2.19/2.19 | 33.6 |
| d-*a*Thr3 | NH | 8.19 | - |
|  | CHα | 4.22 | 59.5 |
|  | CHβ | 5.09 | 71.2 |
|  | CH_3_γ | 1.17 | 18.0 |
| l-Leu4 | NH | 7.12 | - |
|  | CHα | 4.07 | 54.5 |
|  | CH_2_β | 1.76/1.54 | 40.4 |
|  | CHγ | 1.60 | 24.2 |
|  | CH_3_δ1 | 0.95 | 23.0 |
|  | CH_3_δ2 | 0.87 | 23.0 |
| d-Leu5 | NH | 9.41 | - |
|  | CHα | 3.98 | 53.6 |
|  | CH_2_β | 1.84/1.78 | *n.a.* |
|  | CHγ | 1.71 | 24.6 |
|  | CH_3_δ1 | 0.95 | 21.7 |
|  | CH_3_δ2 | 0.84 | 20.3 |
| d-Ser6 | NH | 8.23 | - |
|  | CHα | 4.46 | 57.5 |
|  | CH_2_β | 4.05/3.96 | 59.9 |
| l-Leu7 | NH | 7.69 | - |
|  | CHα | 4.40 | 53.2 |
|  | CH_2_β | 1.56/1.43 | 43.4 |
|  | CHγ | 1.59 | 24.1 |
|  | CH_3_δ1 | 0.86 | 23.0 |
|  | CH_3_δ2 | 0.82 | 21.5 |
| l-Ile8 | NH | 7.33 | - |
|  | CHα | 4.29 | 58.0 |
|  | CHβ | 2.01 | 36.0 |
|  | CH_2_γ1 | 1.51/1.14 | 24.8 |
|  | CH_3_γ2 | 0.89 | 15.7 |
|  | CH_3_δ | 0.82 | 10.6 |
| 3-OH C10:0 | CH_2_α | 2.61/2.47 | 43.6 |
|  | CHβ | *n.a.* | *n.a.* |
| *n.a.* = not assigned | | | |

**Table S3.** The ^1^H and ^13^C chemical shifts of **viscosin (9:7)** in DPC-*d*38 solution at 310.0 K.

| Residue | Group or atom | *δ*_H_ / ppm | *δ*_C_ / ppm |
| --- | --- | --- | --- |
| l-Leu1 | NH | 9.43 | - |
|  | CHα | 4.00 | 52.1 |
|  | CH_2_β | 1.89/1.67 | 38.7 |
|  | CHγ | 1.82 | 24.4 |
|  | CH_3_δ1 | 0.97 | 21.7 |
|  | CH_3_δ2 | 0.97 | 23.0 |
| d-Glu2 | NH | 8.97 | - |
|  | CHα | 4.13 | 56.8 |
|  | CH_2_β | 2.08/2.08 | 26.9 |
|  | CH_2_γ | 2.36/2.31 | 33.8 |
| d-*a*Thr3 | NH | 7.93 | - |
|  | CHα | 4.26 | 60.3 |
|  | CHβ | 5.42 | 70.5 |
|  | CH_3_γ | 1.41 | 17.5 |
| d-Val4 | NH | 8.22 | - |
|  | CHα | 3.44 | 64.9 |
|  | CHβ | 2.29 | 28.9 |
|  | CH_3_γ1 | 1.00 | 20.3 |
|  | CH_3_γ2 | 0.98 | 19.0 |
| l-Leu5 | NH | 8.55 | - |
|  | CHα | 3.94 | 53.0 |
|  | CH_2_β | 2.07/1.68 | 41.1 |
|  | CHγ | 1.68 | 24.8 |
|  | CH_3_δ1 | 0.99 | 21.4 |
|  | CH_3_δ2 | 0.95 | 23.6 |
| d-Ser6 | NH | 7.37 | - |
|  | CHα | 4.60 | *n.a.* |
|  | CH_2_β | 4.22/4.02 | 63.1 |
| l-Leu7 | NH | 7.89 | - |
|  | CHα | 4.28 | 54.3 |
|  | CH_2_β | 2.09/2.09 | *n.a.* |
|  | CHγ | 2.09 | 24.5 |
|  | CH_3_δ1 | 1.05 | 21.2 |
|  | CH_3_δ2 | 1.00 | 23.0 |
| d-Ser8 | NH | 8.30 | - |
|  | CHα | 4,65 | *n.a.* |
|  | CH_2_β | 4.00/3.87 | 61.8 |
| l -Ile9 | NH | 6.87 | - |
|  | CHα | 4.71 | *n.a.* |
|  | CHβ | 2.10 | 36.2 |
|  | CH_2_γ | 1.23/1.03 | 24.2 |
|  | CH_3_γ | 0.92 | 13.9 |
|  | CH_3_δ | 0.92 | 11.8 |
| 3-OH C10:0 | CH_2_α | 2.78/2.53 | 43.7 |
|  | CHβ | 4.18 | 68.4 |
| *n.a.* = not assigned | | | |

**Table S4.** The ^1^H and ^13^C chemical shifts of **orfamide A (10:8)** in DPC-*d*38 solution at 310.0 K.

| Residue | Group or atom | *δ*_H_ / ppm | *δ*_C_ / ppm |
| --- | --- | --- | --- |
| l-Leu1 | NH | 9.43 | - |
|  | CHα | 3.99 | 52.0 |
|  | CH_2_β | 1.72/1.71 | 37.6 |
|  | CHγ | 1.80 | 24.3 |
|  | CH_3_δ1 | 0.98 | 23.5 |
|  | CH_3_δ2 | 0.98 | 21.5 |
| d-Glu2 | NH | 9.09 | - |
|  | CHα | 4.05 | 57.1 |
|  | CH_2_β | 2.09/2.09 | 26.6 |
|  | CH_2_γ | 2.38/2.38 | 33.7 |
| d-*a*Thr3 | NH | 7.81 | - |
|  | CHα | 4.34 | 60.1 |
|  | CHβ | 5.31 | 70.9 |
|  | CH_3_γ | 1.44 | 17.8 |
| d-*a*Ile4 | NH | 7.94 | - |
|  | CHα | 3.70 | 63.6 |
|  | CHβ | 2.19 | 35.7 |
|  | CH_2_γ | 1.48/1.12 | 25.4 |
|  | CH_3_γ | 0.93 | 11.1 |
|  | CH_3_δ | 0.98 | 16.5 |
| d-Leu5 | NH | 8.35 | - |
|  | CHα | 4.06 | 54.6 |
|  | CH_2_β | 1.85/1.71 | 38.7 |
|  | CHγ | 1.90 | 24.7 |
|  | CH_3_δ1 | 0.97 | 21.5 |
|  | CH_3_δ2 | 0.94 | 20.1 |
| d-Ser6 | NH | 7.58 | - |
|  | CHα | 4.44 | 56.9 |
|  | CH_2_β | 4.15 | 61.2 |
| l -Leu7 | NH | 7.88 | - |
|  | CHα | 4.60 | 53.3 |
|  | CH_2_β | 1.84/1.62 | 41.8 |
|  | CHγ | 1.75 | 24.4 |
|  | CH_3_δ1 | 1.02 | 22.8 |
|  | CH_3_δ2 | 0.96 | 20.8 |
| l-Leu8 | NH | 7.44 | - |
|  | CHα | 4.42 | 53.3 |
|  | CH_2_β | 1.86/1.77 | 41.7 |
|  | CHγ | 1.93 | 24.7 |
|  | CH_3_δ1 | 1.00 | 23.2 |
|  | CH_3_δ2 | 0.95 | 22.6 |
| d-Ser9 | NH | 7.51 | - |
|  | CHα | 4.29 | 58.0 |
|  | CH_2_β | 4.00/3.98 | 60.5 |
| l-Val10 | NH | 6.92 | - |
|  | CHα | 4.68 | *n.a.* |
|  | CHβ | 2.34 | 30.7 |
|  | CH_3_γ1 | 0.92 | 18.5 |
|  | CH_3_γ2 | 0.76 | 17.0 |
| 3-OH C14:0 | CH_2_α | 2.81/2.55 | 44.0 |
|  | CHβ | 4.13 | 68.3 |

**Table S5.** The ^1^H and ^13^C chemical shifts of **arthrofactin A (11:9)** in DPC-*d*38 solution at 310.0 K.

| Residue | Group or atom | *δ*_H_ / ppm | *δ*_C_ / ppm |
| --- | --- | --- | --- |
| d-Leu1 | NH | 9.26 | - |
|  | CHα | 3.98 | 56.1 |
|  | CH_2_β | 1.79/1.75 | 39.1 |
|  | CHγ | 1.85 | 24.5 |
|  | CH_3_δ1 | 1.05 | 22.0 |
|  | CH_3_δ2 | 1.00 | 22.2 |
| d-Asp2 | NH | 9.00 | - |
|  | CHα | 4.37 | 53.6 |
|  | CH_2_β | 2.86/2.80 | 35.3 |
| d-*a*Thr3 | NH | 7.63 | - |
|  | CHα | 4.37 | 59.7 |
|  | CHβ | 5.68 | 69.4 |
|  | CH_3_γ | 1.38 | 17.3 |
| d-Leu4 | NH | 8.37 | - |
|  | CHα | 4.09 | 55.9 |
|  | CH_2_β | 1.88/1.77 | 39.5 |
|  | CHγ | 1.77 | 24.9 |
|  | CH_3_δ1 | 0.98 | 20.4 |
|  | CH_3_δ2 | 0.93 | 20.1 |
| d-Leu5 | NH | 8.28 | - |
|  | CHα | 4.12 | 53.6 |
|  | CH_2_β | 1.88/1.78 | 38.6 |
|  | CHγ | 1.96 | 24.6 |
|  | CH_3_δ1 | 0.98 | 22.9 |
|  | CH_3_δ2 | 0.93 | 22.4 |
| d-Ser6 | NH | 7.66 | - |
|  | CHα | 4.27 | 58.4 |
|  | CH_2_β | 4.27/4.19 | 61.5 |
| l-Leu7 | NH | 7.32 | - |
|  | CHα | 4.29 | 53.5 |
|  | CH_2_β | 1.89/1.73 | 40.3 |
|  | CHγ | 1.89 | 24.6 |
|  | CH_3_δ1 | 1.02 | 23.1 |
|  | CH_3_δ2 | 0.98 | 22.1 |
| d-Ser8 | NH | 8.20 | - |
|  | CHα | 4.27 | 57.4 |
|  | CH_2_β | 3.88/3.80 | 61.2 |
| l-Ile9 | NH | 8.13 | - |
|  | CHα | 4.16 | 60.3 |
|  | CHβ | 1.76 | 36.9 |
|  | CH_2_γ | 1.60/1.13 | 24.9 |
|  | CH_3_γ | 1.00 | 15.2 |
|  | CH_3_δ | 0.93 | 10.9 |

**Table S5.** (continued)

| Residue | Group or atom | *δ*_H_ / ppm | *δ*_C_ / ppm |
| --- | --- | --- | --- |
| l -Ile10 | NH | 7.36 | - |
|  | CHα | 4.40 | 56.6 |
|  | CHβ | 1.78 | 39.3 |
|  | CH_2_γ | 1.53/1.15 | 24.8 |
|  | CH_3_γ | 0.98 | 15.1 |
|  | CH_3_δ | 0.93 | 11.4 |
| l-Asp11 | NH | 8.90 | - |
|  | CHα | 4.42 | 51.4 |
|  | CH_2_β | 2.80/2.80 | 36.4 |
| 3-OH C10:0 | CH_2_α | 2.82/2.70 | 43.4 |
|  | CHβ | 4.16 | 68.6 |

**Table S6.** The ^1^H and ^13^C chemical shifts of **tanniamide A (12:10)** in DPC-*d*38 solution at 310 K.

| Residue | Group or atom | *δ*_H_ / ppm | *δ*_C_ / ppm |
| --- | --- | --- | --- |
| d-Leu1 | NH | 9.37 | - |
|  | CHα | 3.93 | 56.0 |
|  | CH_2_β | 1.80/1.67 | 39.1 |
|  | CHγ | 1.87 | 24.5 |
|  | CH_3_δ1 | 1.02 | 22.5 |
| d-Asp2 | NH | 8.94 | - |
|  | CHα | 4.31 | 53.6 |
|  | CH_2_β | 2.72/2.66 | 36.8 |
| d-*a*Thr3 | NH | 7.62 | - |
|  | CHα | 4.40 | 59.8 |
|  | CHβ | 5.71 | 69.3 |
|  | CH_3_γ | 1.40 | 17.3 |
| d-*a*Ile4 | NH |  |  |
|  | CHα | 3.70 | 62.8 |
|  | CHβ | 1.86 | 36.0 |
|  | CH_2_γ | 1.58/1.21 | 25.1 |
|  | CH_3_γ | 1.02 | 15.8 |
|  | CH_3_δ | 0.90 | 10.5 |
| l-Leu5 | NH | 8.61 | - |
|  | CHα | 4.00 | 53.4 |
|  | CH_2_β | 2.02/1.85 | 38.0 |
|  | CHγ | 1.72 | 24.7 |
| d-Gln6 | NH | 7.41 | - |
|  | CHα | 4.19 | 55.2 |
|  | CH_2_β | 2.35/2.35 | 26.2 |
|  | CH_2_γ | 2.76/2.59 | 31.8 |
| l-Leu7 | NH | 7.92 | - |
|  | CHα | 4.19 | 54.2 |
|  | CH_2_β | 1.94/1.53 | 41.0 |
|  | CHγ | 1.88 | 23.7 |
| d-Ser8 | NH | 8.49 | - |
|  | CHα | 4.31 | 57.5 |
|  | CH_2_β | 3.98/3.97 | 61.2 |
| l-Leu9 | NH | 8.35 | - |
|  | CHα | 4.25 | 53.3 |
|  | CH_2_β | 1.83/1.73 | 39.5 |
|  | CHγ | 1.74 | 24.3 |
|  | CH_3_δ1 | 0.92 | 20.2 |
| d-Gln10 | NH | 7.96 | - |
|  | CHα | 4.47 | 52.4 |
|  | CH_2_β | 2.26/1.76 | 27.2 |
|  | CH_2_γ | 2.27/2.27 | 31.2 |
| l-Ile11 | NH | 6.73 | - |
|  | CHα | 4.34 | 57.0 |
|  | CHβ | 1.66 | 37.5 |
|  | CH_2_γ | 1.50/1.13 | 24.5 |
|  | CH_3_γ | 0.98 | 15.1 |
|  | CH_3_δ | 0.88 | 11.0 |

**Table S6.** (continued)

| Residue | Group or atom | *δ*_H_ / ppm | *δ*_C_ / ppm |
| --- | --- | --- | --- |
| l-Gln12 | NH | 8.97 | - |
|  | CHα | 4.32 | 52.6 |
|  | CH_2_β | 2.16/2.00 | 27.1 |
|  | CH_2_γ | 2.33/2.30 | 33.8 |
| 3-OH C12:1 | CH_2_α | 2.86/2.76 | 42.2 |
|  | CHβ | 4.35 | 68.0 |

**Table S7.** The ^1^H and ^13^C chemical shifts of **putisolvin I (12:4)** in DPC-*d*38 solution at 298.0 K

| Residue | Group or atom | *δ*_H_ / ppm | *δ*_C_ / ppm |
| --- | --- | --- | --- |
| l-Leu1 | NH | 8.95 | - |
|  | CHα | 3.89 | 52.2 |
|  | CH_2_β | 1.80/1.59 | 37.4 |
|  | CHγ | 1.59 | 24.2 |
|  | CH_3_δ1 | 0.86 | 20.9 |
|  | CH_3_δ2 | 0.85 | 22.3 |
| d-Glu2 | NH | 8.82 | - |
|  | CHα | 4.00 | 56.9 |
|  | CH_2_β | 1.97/1.97 | 26.2 |
|  | CH_2_γ | 2.28/2.21 | 33.5 |
| d-Leu3 | NH | 7.88 | - |
|  | CHα | 4.02 | 55.2 |
|  | CH_2_β | 1.72/1.71 | 38.8 |
|  | CHγ | 1.54 | 24.6 |
|  | CH_3_δ1 | 0.89 | 22.4 |
|  | CH_3_δ2 | 0.85 | 23.6 |
| l-Leu4 | NH | 8.86 | - |
|  | CHα | 3.64 | 51.8 |
|  | CH_2_β | 1.81/1.54 | 36.6 |
|  | CHγ | 1.81 | 24.5 |
|  | CH_3_δ1 | 0.82 | 23.0 |
|  | CH_3_δ2 | 0.79 | 21.0 |
| d-Gln5 | NH | 8.43 | - |
|  | CHα | 3.85 | 56.6 |
|  | CH_2_β | 2.08/2.08 | 26.1 |
|  | CH_2_γ | 2.40/2.31 | 31.5 |
| d-Ser6 | NH | 7.88 | - |
|  | CHα | 4.37 | 58.2 |
|  | CH_2_β | 3.94/3.92 | 60.4 |
| d-Val7 | NH | 8.12 | - |
|  | CHα | 3.55 | 64.3 |
|  | CHβ | 2.32 | 28.6 |
|  | CH_3_γ1 | 1.00 | 20.8 |
|  | CH_3_γ2 | 0.90 | 19.1 |
| d-Leu8 | NH | 7.85 | - |
|  | CHα | 4.04 | 54.6 |
|  | CH_2_β | 1.82/1.62 | 38.6 |
|  | CHγ | 1.82 | 24.5 |
|  | CH_3_δ1 | 0.86 | 20.9 |
|  | CH_3_δ2 | 0.85 | 22.3 |
| d-Ser9 | NH | 7.46 | - |
|  | CHα | 4.96 | 51.7 |
|  | CH_2_β | 4.88/4.19 | 66.4 |
| l-Leu10 | NH | 8.17 | - |
|  | CHα | 4.19 | 54.5 |
|  | CH_2_β | 2.07/1.48 | 39.8 |
|  | CHγ | 1.79 | 24.5 |
|  | CH_3_δ1 | 0.90 | 22.9 |

**Table S7.** (continued)

| Residue | Group or atom | *δ*_H_ / ppm | *δ*_C_ / ppm |
| --- | --- | --- | --- |
| l-Leu10 | CH_3_δ2 | 0.84 | 20.8 |
| l-Val11 | NH | 7.44 | - |
|  | CHα | 3.55 | 61.9 |
|  | CHβ | 1.89 | 28.7 |
|  | CH_3_γ1 | 0.86 | 18.6 |
|  | CH_3_γ2 | 0.81 | 18.9 |
| l-Ser12 | NH | 8.63 | - |
|  | CHα | 4.72 | *n.a.* |
|  | CH_2_β | 4.01/3.68 | 62.0 |
| C6:0 | CH_2_α | 2.40/2.27 | 35.0 |
| *n.a.: not assigned* | | | |

**Table S8.** The ^1^H and ^13^C chemical shifts of **entolysin A (14:5)** in DPC-*d*38 solution at 298.0 K

| Residue | Group or atom | *δ*_H_ / ppm | *δ*_C_ / ppm |
| --- | --- | --- | --- |
| l-Leu1 | NH | 9.10 | - |
|  | CHα | 3.79 | 51.8 |
|  | CH_2_β | 1.69/1.63 | 38.5 |
|  | CHγ | 1.69 | 24.2 |
|  | CH_3_δ1 | 0.86 | 23.3 |
|  | CH_3_δ2 | 0.85 | 21.8 |
| d-Glu2 | NH | 8.77 | - |
|  | CHα | 3.94 | 56.3 |
|  | CH_2_β | 2.04/1.94 | 25.1 |
|  | CH_2_γ | 2.45/2.43 | 30.4 |
| d-Gln3 | NH | 7.75 | - |
|  | CHα | 4.07 | 56.8 |
|  | CH_2_β | 2.21/1.97 | 25.7 |
|  | CH_2_γ | 2.40/2.35 | 32.4 |
| d-Val4 | NH | 7.98 | - |
|  | CHα | 3.47 | 64.0 |
|  | CHβ | 2.32 | 28.5 |
|  | CH_3_γ1 | 0.96 | 20.7 |
|  | CH_3_γ2 | 0.83 | 18.6 |
| d-Leu5 | NH | 8.07 | - |
|  | CHα | 3.90 | 55.7 |
|  | CH_2_β | 1.67/1.67 | 38.4 |
|  | CHγ | 1.67 | 24.2 |
|  | CH_3_δ1 | 0.84 | 23.7 |
|  | CH_3_δ2 | 0.82 | 21.6 |
| d-Gln6 | NH | 7.96 | - |
|  | CHα | 3.99 | 56.4 |
|  | CH_2_β | 2.21/2.07 | 25.7 |
|  | CH_2_γ | 2.55/2.35 | 31.6 |
| d-Val7 | NH | 7.78 | - |
|  | CHα | 3.71 | 64.1 |
|  | CHβ | 2.25 | 28.8 |
|  | CH_3_γ1 | 1.07 | 20.8 |
|  | CH_3_γ2 | 0.85 | 18.9 |
| d-Leu8 | NH | 8.21 | - |
|  | CHα | 3.97 | 55.1 |
|  | CH_2_β | 1.87/1.87 | 38.8 |
|  | CHγ | 1.87 | 24.0 |
|  | CH_3_δ1 | 0.79 | 23.2 |
|  | CH_3_δ2 | 0.76 | 20.2 |
| d-Gln9 | NH | 7.75 | - |
|  | CHα | 4.02 | 54.9 |
|  | CH_2_β | 2.05/2.05 | 26.3 |
|  | CHγ | 2.47/2.29 | 31.8 |
| d-Ser10 | NH | 7.63 | - |
|  | CHα | 4.52 | *n.a.* |
|  | CH_2_β | 4.57/4.35 | *n.a.* |

**Table S8.** (continued)

| Residue | Group or atom | *δ*_H_ / ppm | *δ*_C_ / ppm |
| --- | --- | --- | --- |
| l-Val11 | NH | 7.93 | - |
|  | CHα | 4.06 | 59.4 |
|  | CHβ | 2.18 | 29.6 |
|  | CH_3_γ1 | 1.03 | 19.0 |
|  | CH_3_γ2 | 0.95 | 17.0 |
| l-Leu12 | NH | 9.30 | - |
|  | CHα | 3.83 | 53.2 |
|  | CH_2_β | 1.83/1.69 | 36.9 |
|  | CHγ | 1.56 | 24.7 |
|  | CH_3_δ1 | 0.87 | 20.7 |
|  | CH_3_δ2 | 0.86 | 21.2 |
| l-Ser13 | NH | 7.92 | - |
|  | CHα | 3.99 | 58.2 |
|  | CH_2_β | 4.17/3.96 | 60.4 |
| l-Ile14 | NH | 8.19 | - |
|  | CHα | 4.61 | *n.a.* |
|  | CHβ | 2.14 | 37.2 |
|  | CH_2_γ | 1.50 | 24.9 |
|  | CH_3_γ | 1.15 | 16.9 |
|  | CH_3_δ | 0.86 | 12.2 |
| 3-OH C10:0 | CHα | 2.73/2.39 | 43.6 |
|  | CHβ | 3.97 | 68.2 |
| *n.a.: not assigned* | | | |

**Table S9.** The ^1^H and ^13^C chemical shifts of **xantholysin A (14:8)** in DPC-*d*38 solution at 298.0 K.

| Residue | Group or atom | *δ*_H_ / ppm | *δ*_C_ / ppm |
| --- | --- | --- | --- |
| l-Leu1 | NH | 9.19 | - |
|  | CHα | 3.82 | 51.8 |
|  | CH_2_β | 1.66/1.61 | 38.1 |
|  | CHγ | 1.65 | 24.2 |
|  | CH_3_δ1 | 0.86 | 21.0 |
|  | CH_3_δ2 | 0.85 | 20.9 |
| d-Glu2 | NH | 8.91 | - |
|  | CHα | 3.91 | 57.2 |
|  | CH_2_β | 2.00/1.95 | 26.7 |
|  | CH_2_γ | 2.26/2.20 | 33.7 |
| d-Gln3 | NH | 7.79 | - |
|  | CHα | 4.09 | 56.1 |
|  | CH_2_β | 2.18/2.03 | 25.9 |
|  | CH_2_γ | 2.40/2.37 | 31.9 |
| d-Val4 | NH | 8.09 | - |
|  | CHα | 3.54 | 63.9 |
|  | CHβ | 2.22 | 28.8 |
|  | CH_3_γ1 | 0.95 | 20.4 |
|  | CH_3_γ2 | 0.79 | 18.5 |
| d-Leu5 | NH | 8.32 | - |
|  | CHα | 3.78 | 56.0 |
|  | CH_2_β | 1.79/1.57 | 38.9 |
|  | CHγ | 1.56 | 24.5 |
|  | CH_3_δ1 | 0.89 | 22.9 |
|  | CH_3_δ2 | 0.83 | 22.9 |
| l-Gln6 | NH | 8.40 | - |
|  | CHα | 3.78 | 53.2 |
|  | CH_2_β | 2.08/2.07 | 23.8 |
|  | CH_2_γ | 2.32/2.22 | 31.2 |
| d-Ser7 | NH | 7.66 | - |
|  | CHα | 4.20 | 56.7 |
|  | CH_2_β | 4.22/4.21 | 63.4 |
| d-Val8 | NH | 8.22 | - |
|  | CHα | 3.54 | 64.6 |
|  | CHβ | 2.26 | 28.9 |
|  | CH_3_γ1 | 0.98 | 20.2 |
|  | CH_3_γ2 | 0.87 | 18.8 |
| d-Leu9 | NH | 8.37 | - |
|  | CHα | 4.04 | 54.6 |
|  | CH_2_β | 1.83/1.80 | 38.9 |
|  | CHγ | 1.52 | 24.2 |
|  | CH_3_δ1 | 0.90 | 23.1 |
|  | CH_3_δ2 | 0.87 | 23.1 |
| d-Gln10 | NH | 7.65 | - |
|  | CHα | 4.26 | 54.2 |
|  | CH_2_β | 2.27/1.98 | 26.1 |
|  | CH_2_γ | 2.55/2.38 | 31.9 |

**Table S9.** (continued)

| Residue | Group or atom | *δ*_H_ / ppm | *δ*_C_ / ppm |
| --- | --- | --- | --- |
| l-Leu11 | NH | 7.80 | - |
|  | CHα | 4.26 | 53.2 |
|  | CH_2_β | 1.91/1.49 | 40.8 |
|  | CHγ | 1.71 | 24.4 |
|  | CH_3_δ1 | 0.92 | 20.9 |
|  | CH_3_δ2 | 0.85 | 20.9 |
| l-Leu12 | NH | 7.78 | - |
|  | CHα | 4.34 | 52.5 |
|  | CH_2_β | 1.58/1.50 | 40.8 |
|  | CHγ | 1.70 | 24.3 |
|  | CH_3_δ1 | 0.89 | 23.3 |
|  | CH_3_δ2 | 0.85 | 23.3 |
| d-Gln13 | NH | 7.37 | - |
|  | CHα | 4.18 | 52.7 |
|  | CH_2_β | 2.13/1.85 | 25.2 |
|  | CH_2_γ | 2.26/2.14 | 31.1 |
| l-Ile14 | NH | 7.31 | - |
|  | CHα | 4.30 | 57.4 |
|  | CHβ | 1.86 | 36.1 |
|  | CH_2_γ | 1.30/1.07 | 24.7 |
|  | CH_3_γ | 0.82 | 15.3 |
|  | CH_3_δ | 0.80 | 11.0 |
| 3-OH C10:0 | CH_2_α | 2.75/2.42 | 43.6 |
|  | CHβ | 3.96 | 68.3 |

**Table S10.** The ^1^H and ^13^C chemical shifts of **pentorfamide (10:5)** in DPC-*d*38 solution at 325.0 K.

| Residue | Group or atom | *δ*_H_ / ppm | *δ*_C_ / ppm |
| --- | --- | --- | --- |
| l-Leu1 | NH | 9.03 | - |
|  | CHα | 4.20 | 52.7 |
|  | CH_2_β | 1.96/1.91 | 39.2 |
|  | CHγ | 1.97 | 24.8 |
|  | CH_3_δ1 | 1.13 | 23.6 |
|  | CH_3_δ2 | 1.11 | 21.4 |
| d-Glu2 | NH | 8.94 | - |
|  | CHα | 4.20 | 56.9 |
|  | CH_2_β | 2.25/2.20 | 27.2 |
|  | CH_2_γ | 2.48/2.45 | 34.1 |
| d-*a*Thr3 | NH | 8.19 | - |
|  | CHα | 4.37 | 61.8 |
|  | CHβ | 4.43 | 66.1 |
|  | CH_3_γ | 1.44 | 19.1 |
| d-*a*Ile4 | NH | 7.65 | - |
|  | CHα | 4.30 | 60.2 |
|  | CHβ | 2.24 | 35.8 |
|  | CH_2_γ | 1.68/1.48 | 25.9 |
|  | CH_3_γ | 1.22 | 15.9 |
|  | CH_3_δ | 1.10 | 11.1 |
| d-Leu5 | NH | 7.73 | - |
|  | CHα | 4.44 | 53.2 |
|  | CH_2_β | 2.02/1.90 | 38.9 |
|  | CHγ | 1.91 | 24.7 |
|  | CH_3_δ1 | 1.13 | 23.1 |
|  | CH_3_δ2 | 1.06 | 20.6 |
| d-Ser6 | NH | 7.98 | - |
|  | CHα | 4.81 | 53.5 |
|  | CH_2_β | 4.72/4.63 | 64.5 |
| l-Leu7 | NH | 7.98 | - |
|  | CHα | 4.50 | 53.7 |
|  | CH_2_β | 2.02/1.71 | 40.6 |
|  | CHγ | 1.99 | 24.6 |
|  | CH_3_δ1 | 1.14 | 23.6 |
|  | CH_3_δ2 | 1.13 | 21.3 |
| l-Leu8 | NH | 8.93 | - |
|  | CHα | 4.40 | 52.6 |
|  | CH_2_β | 2.07/2.02 | 38.4 |
|  | CHγ | 1.84 | 25.1 |
|  | CH_3_δ1 | 1.14 | 23.1 |

**Table S10.** (continued)

|  | CH_3_δ2 | 1.12 | 21.2 |
| --- | --- | --- | --- |
| d-Ser9 | NH | 7.76 | - |
|  | CHα | 4.78 | 58.5 |
|  | CH_2_β | *n.a.* | *n.a.* |
| l-Val10 | NH | 7.80 | - |
|  | CHα | 4.52 | 59.4 |
|  | CHβ | 2.59 | 29.8 |
|  | CH_3_γ1 | 1.15 | 19.4 |
|  | CH_3_γ2 | 1.11 | 18.3 |
| 3-OH C14:0 | CH_2_α | 2.89/2.67 | 44.2 |
|  | CHβ | 4.15 | 68.7 |
|  | CHγ | 1.66 | 36.5 |
| *n.a.: not assigned* | | | |

## **2.5. Characterization of CLiP incorporation into DPC micelles**

The incorporation of CLiPs into DPC micelles was established using ^1^H (for the CLiP) and ^31^P (for DPC micelles) translational diffusion NMR measurements, ensuring that structural data results from DPC micelle-associated CLiPs. The diffusion coefficient of the DPC molecules was determined from ^31^P diffusion measurements targeting the phosphocholine headgroup, while that of the CLiP was monitored from the methyl resonances in the ^1^H NMR spectrum. The corresponding diffusion coefficients *D*_CLiP_ and *D*_DPC_ show always comparable values, that is, differ only by 5–10%. Accordingly, under the conditions of the NMR experiments the incorporation of the investigated CLiPs into the water:DPC interface can be considered close to 100%. All diffusion NMR measurements used a Bruker Avance III spectrometer operating at a respective ^1^H and ^31^P frequency of 500.13 MHz and 202.4 MHz equipped with a BBI-Z probe. For the DOSY measurements we used convection compensated sequences. Either the standard Bruker double stimulated echo with bipolar gradients or a double stimulated echo with monopolar gradients with an extended phase cycle was used. The diffusion encoding/decoding gradients were varied linearly between 2% and 95% of their maximum output over 32 increments. The duration of these gradients and the diffusion delay time were chosen so that at the highest gradient strength the intensity of the signals of interest was decreased to at least 10% of the intensity at the lowest gradient strength. The obtained intensity decays were fitted to the appropriate Stejskal–Tanner equation^[92]^ using an in-house Matlab script to obtain the corresponding translational diffusion coefficients which are collected in SI Table S11 for each CLiP+DPC system. The error on the fitted diffusion coefficients was determined using Monte Carlo analysis as implemented in the script.

**Table S11.** The translational diffusional coefficient of the DPC micelles (*D*_DPC_) and the CLiPs (*D*_CLiP_) in the applied NMR samples. The error on the fitted parameters is shown in parenthesis. The temperature of the measurements (*T*) corresponds to that of the 2D NMR experiments.

| Ps-CLiP (*l*:*m*) | *D*_DPC_ / μm^2.^s^–1^ | *D*_CLiP_ / μm^2.^s^–1^ | *T* / K |
| --- | --- | --- | --- |
| MDN-0066 (8:6) | 96.9 (6.3) | 85.2 (1.6) | 298.0 |
| viscosin (9:7) | 124.0 (4.9) | 118.0 (1.2) | 310.0 |
| orfamide (10:8) | 122.4 (6.4) | 107.8 (1.3) | 310.0 |
| arthrofactin (11:9) | 120.7 (5.3) | 113.0 (1.6) | 310.0 |
| tanniamide (12:10) | 119.0 (2.3) | 120.0 (2.1) | 310.0 |
| putisolvin (12:4) | 93.6 (4.4) | 79.1 (0.6) | 298.0 |
| entolysin (14:5) | 101.0 (4.7) | 84.2(2.37) | 298.0 |
| xantholysin (14:8) | 109.5 (3.1) | 104.8 (0.7) | 310.0 |
| pentorfamide (10:5) | 116.2(3.6) | 125.2(2.2) | 310.0 |

In all but 2 cases, a notable difference between both is apparent with *D*_CLiP_ < *D*_DPC_. While the impact of CLiPs on DPC micellar behavior merits future investigations, we propose these support a high to complete level of incorporation of CLiPs into the DPC micelles, thus ensuring all structural data used in this work results from CLiPs in DPC micelle associated form.

# 3. NMR structure calculation protocol

## **3.1. Initial CNS structure generation**

The nOe data for structure generation were collected from 2D NOESY spectra using 100 ms mixing time to maximize nOe build-up while avoiding spin-diffusion. Following nOe cross-peak assignment within CcpNMR Analysis (v2.4)^[93,94]^ their volumes were converted into interproton distances using the standard calibration routine whereby the nOe peak list's average volume corresponds to a distance of 3.2 Å. Lower and upper limit bounds were set to ±20% of the individual distances. The micelle-bound CLiP conformations were determined in two steps. An initial structural ensemble was generated by imposing the distance restraints during structure calculations with the CNS 1.21 software package^[58,59]^ with simulated annealing and torsion angle parameters set to the recommended default values for proteins. The original parallhdg.prot and topallhdg.prot files were modified to include the depsi bond, the acyl chain, and d-configured amino acids. The Glu/Asp side-chains were deprotonated for the NMR structure calculations. For each CLiP, calculations started from an extended conformation and were repeated 100 times to sample the conformational space defined by the structural data. An ensemble comprised of the 10 lowest energy structures were selected for RMSD analysis (Table 2) and deposited together with a refined representative structure (see below) and the distance restraint list in the protein data bank (PDB) database (Table 1).

## **3.2. Structure refinement via molecular dynamics with explicit DPC micelle**

The lowest energy CNS structure from the initial ensemble was subjected to further refinement using unrestrained molecular dynamics (MD) simulations in an explicit water:DPC environment using AMBER14. All simulations were run on a single NVidia GTX680 or GTX780 GPU using the GPU implementation of pmemd provided in the AMBER14 simulation package.^[95]^ The non-standard residues of the CLiP molecules such as the N-terminal acyl chain cap or the amino acids included in the ester bond were defined individually for the AMBER simulations. First, a geometry optimization of the capped building block was performed at B3LYP/6-31G* level of theory using the Gaussian 09 program package.^[96]^ Subsequently, the amino acid building blocks were parametrized using GAFF in Antechamber^[97,98]^ while the acyl chain of the peptide was parametrized using the LIPID14 force field.^[99]^

The DPC molecule was parametrized using the LIPID14 force field without QM geometry optimization. To create an explicit DPC micelle the individual DPC monomers were placed in a single-layer grid of 8x7 monomers to match the average aggregation number (56)^[89]^ of DPC and let self-associate via unrestrained molecular dynamics simulation with identical parameters as the subsequent CLiP structure refinement trajectories. The DPC monomers were separated from each other by ~5 Å along both dimensions. The system was solvated using ~16000 TIP3P water molecules in a 100x80x80 Å^3^ rectangular solvent box. In such aqueous environment the DPC molecules were observed to rearrange themselves into a spherical association within ~3 ns with the acyl chains forming a hydrophobic core and the polar head groups pointing towards the water molecules as expected for a micelle. The forming DPC micelle remained compact and was simulated for 100 ns in total to ascertain that it is equilibrated. The DPC micelle extracted from the last frame of this simulation was used to establish the explicit water:DPC environment to refine the CLiP structures obtained from NMR structure calculations.

The starting topology and coordinate file of the simulations were created using the GUI of the LEaP program (xLEaP) provided with the AMBER simulation package. To create the CLiP+DPC simulation box the lowest energy CNS structure of a particular CLiP was placed 10–15 Å from the surface of the pre-equilibrated DPC micelle. Negatively charged Glu or Asp side-chain(s) were neutralized by Na^+^ counter ion(s). The solutes were subsequently solvated by ~16000 explicit TIP3P water molecules creating a 100x80x80Å^3^ solvent box, resulting in CLiP and DPC concentrations of 2–3 mm and ~150 mm, respectively, similar to the experimental conditions. To pre-equilibrate the system for the production runs, the starting coordinates were optimized in two-steps. In the first stage, the solute molecules were fixed with a strong positional restraint (500 kJ^.^mol^–1^) while the positions of the solvent molecules and the counter ions were submitted to energy minimization. Subsequently, in the second stage, the entire system was subjected to a short (~ps) MD simulation without the application of restraints. Then, the system was allowed to heat up linearly from 0 K to 300 K while a weak potential energy restraint (10 kJ^.^mol^–1^) was applied on the solute molecules. While here the complete trajectory samples the *NVE* ensemble, the temperature is regulated at each heating step using the Langevin-bath. At 300 K a short *NVT* run was applied to adjust the system to the correct density. The production MD simulations were conducted at constant pressure and temperature (*NPT*) at 300 K for 100 ns total simulation time. Periodic boundary conditions were applied along all dimensions. Bond lengths involving hydrogen were held fixed with the SHAKE algorithm, allowing 2 fs timesteps.^[100]^ Temperature scaling was performed using Langevin dynamics with a collision frequency of 1.0 ps^–1^ while the long-range interactions were computed using the particle mesh Ewald summation.^[101]^ For the initial parts of the simulations the non-bonded cutoff was 9 Å to 15–20 Å so as to favor the interaction of the CLiP and the DPC micelle.

## **3.3. Molecular dynamics trajectory analysis**

MD trajectories were visualized using VMD 1.9.1^[102]^ or Pymol v1.3 and analyzed with *cpptraj*.^[103]^ The built-in DBSCAN algorithm of *cpptraj* was used for cluster analysis of the membrane-associated CLiP trajectory. The clustering criterion was defined according to the coordinate RMSD of the mainchain H^N^, C’, Cα, N and amide O atoms to best represent the backbone conformation and intramolecular hydrogen bonding pattern of the trajectory. MinPts=4 was chosen throughout while the *ε* value was defined as the ‘elbow value’ (around 0.3–0.5 Å for all trajectories) of the *k*-dist (4-dist) plot. For all natural CLiPs, one major cluster was found that included >90% of the frames. Other clusters were low in population and not analyzed further. From this major cluster, a single frame representing the conformation with the shortest distance from the cluster centroid was chosen as representing the cluster’s overall ensemble. For each CLiP this refined representative structure was used for discussion and comparative analysis in most of the Results and Discussion of the main manuscript. Importantly, in subsections 2.5. and 2.6 we show the frame-to-frame averages of the simulated CLiP phi, psi backbone torsion angles for the best comparison of the macrocycle conformation of different CLiPs. Quality control of the representative structures was performed against Ramachandran maps for the backbone torsion angles using PROCHECK.^[104,105]^ (SI Section 4.2) The refined MD structures were confronted with the original interproton distance restraints used for the initial CNS structure generation. (Table 2) For each CLiP, coordinates of the refined representative structure are included as conformation #1 in the corresponding entries in the PDB which is followed by the 10 lowest energy members of the generated CNS structure ensemble. (Table 1) Input files for the CNS structure calculations, the subsequent AMBER MD simulations in water:DPC environment as well as the produced 100 ns trajectories are available from the Zenodo data repository. (<https://doi.org/10.5281/zenodo.15024231>)

# 4. Conformational analyses and comparisons of 8 *Pseudomonas* CLiPs

## **4.1. The backbone dynamics of the natural CLiP structures in membrane-bound state**

The backbone RMSD plots of the CLiP conformations during the MD trajectories reflect convergence. (SI Figure S3). The depsi bond adopts a single orientation in most cases except for CLiPs with 4 loop residues (orfamide, entolysin, xantholysin) for which it displays an exchange between two different torsional states. (SI Figure S4) This significantly increases the torsional flexibility of the C-terminal amide plane of orfamide (*δ*(*φ*(Val10),*ψ*(Ser9)) =(35^o^;25^o^)). During the simulations in the membrane-associated state the α_L_-helical motif element proved to be the most rigid part in all CLiP structures typically exhibiting *δ*(*φ* or *ψ*) values of ~10^o^. Regarding the α_L_-helix, increased flexibility in backbone dynamics (20^o^<*δ*(*φ*/*ψ*)<27^o^) could be observed at the depsi bonding d-Ser/d-*allo*-Thr latch residue and/or at the helix terminating d-Ser residues in shorter stapled helices (5<*m*≤8, i.e. MDN-0066, viscosin, orfamide) that are found in less favored positions on the Ramachandran map (SI Figures S5–S7). The macrocycle conformations of the catch pole helices (*m*≤5) appear rather rigid (*δ*(*φ*/*ψ*)<20^o^) with the macrocycle orientation also remaining well-defined with respect to the helical axis throughout the trajectories. The frame-to-frame averaged phi, psi angle values along with their standard deviations are collected in SI Tables S12-S16. For these statistics only the membrane-associated states were considered.


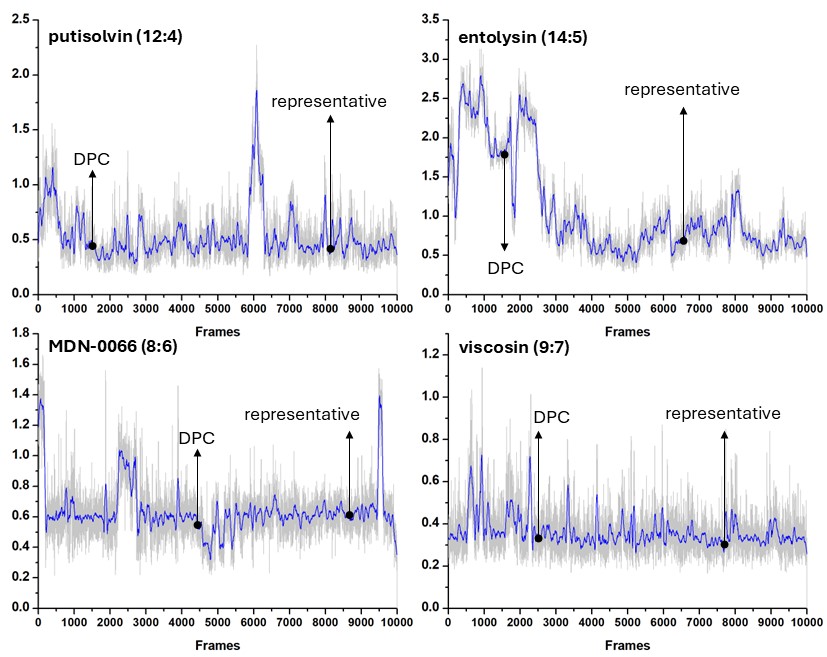


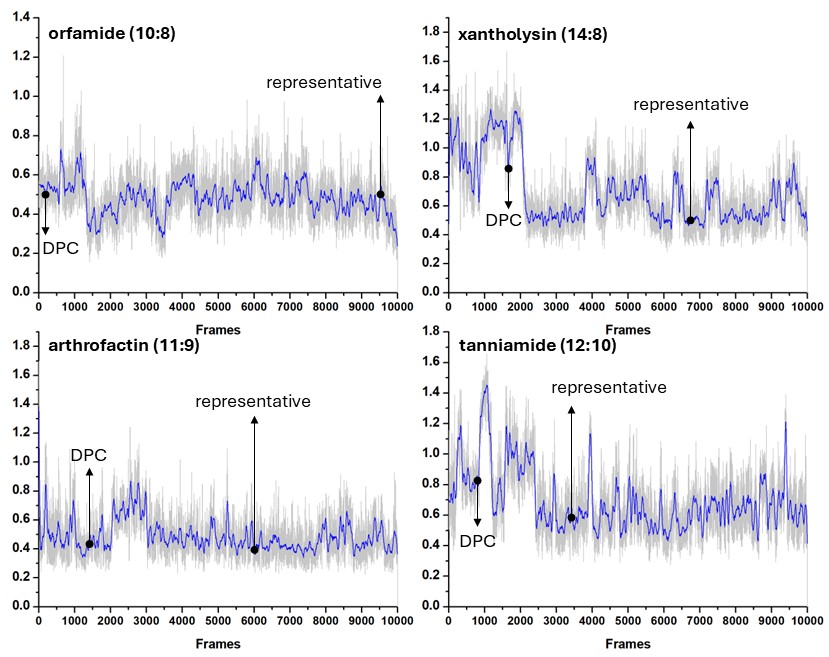


**Figure S3.** The evolution of CLiP backbone RMSD during the 100 ns molecular dynamics trajectories in water:DPC environment compared to the respective last frame structure. Grey: raw data, blue: smoothed data. In each plot two frames are highlighted: the time point when the CLiP molecule and the DPC micelle associate (֕”DPC”); and the frame of the representative CLiP structure of the MD simulation (֕”representative”).


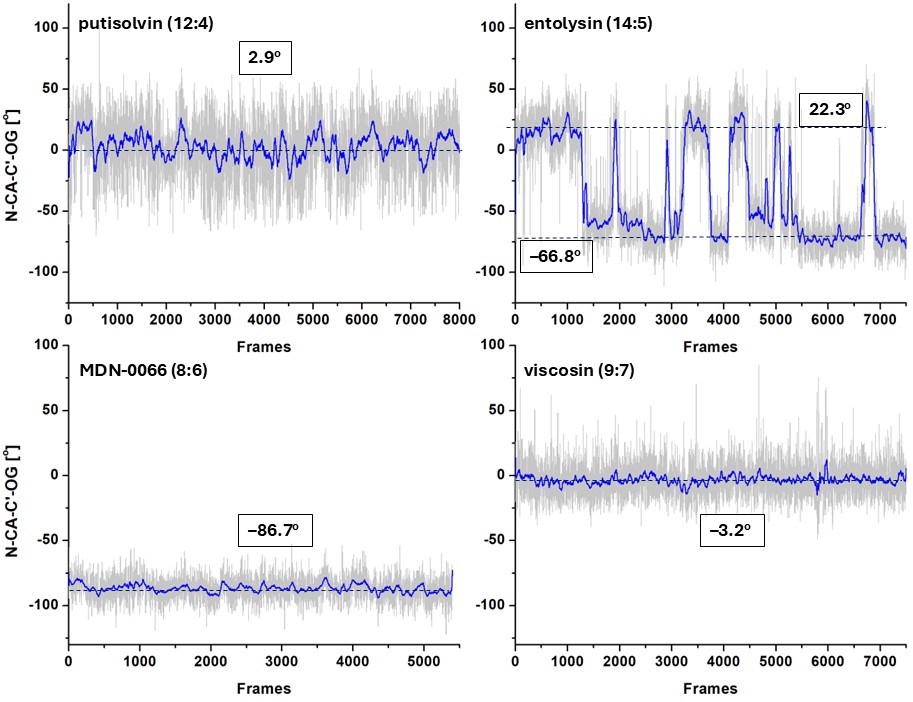


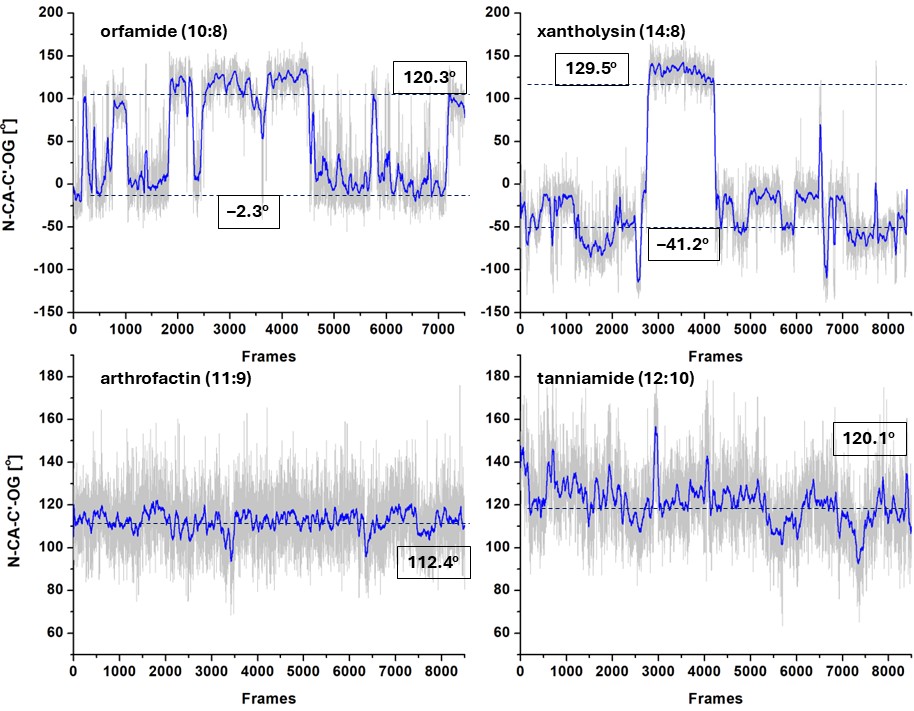


**Figure S4.** The evolution of the C-terminal side-chain-backbone torsion angle defined between the N-CA-C’-OG atoms considering the post-insertion states of the MD trajectories. Grey: raw data, blue: smoothed data. For these plots only the micelle-bound state of the CLiPs was considered. The frame-per-frame average values are indicated in each graph. For orfamide, xantholysin and entolysin the two average values describe the two interchanging states. For the standard deviation of these angle values see SI Tables S12-S16.

**Table S12.** Phi/psi torsion angle data of the MD refined MDN-0066 (8:6) and viscosin (9:7) structures.

| **MDN-0066 (8:6)** | | | | **viscosin (9:7)** | | | |
| --- | --- | --- | --- | --- | --- | --- | --- |
| ***φ***/^o^ | | ***ψ***/^o^ | | ***φ***/^o^ | | ***ψ***/^o^ | |
| **Rep.**  **Struct.** | **MD Average [StDev]** | **Rep.**  **Struct.** | **MD Average [StDev]** | **Rep.**  **Struct.** | **MD Average [StDev]** | **Rep.**  **Struct.** | **MD Average [StDev]** |
| **d-Leu1** | | | | **l-Leu1** | | | |
| 60.9 | 59.9  [12.3] | 43.8 | 38.6  [11.4] | 38.4 | 49.7  [12.9] | 59.7 | 51.8  [13.6] |
| **d-Glu2** | | | | **d-Glu2** | | | |
| 62.8 | 65.8  [8.7] | 33.8 | 31.3  [12.6] | 64.7 | 66.6  [9.3] | 23.1 | 24.3  [10.3] |
| **d-*allo*-Thr3** | | | | **d-*allo*-Thr3** | | | |
| 63.2 | 71.2  [20.0] | 52.5 | 47.6  [7.2] | 73.9 | 72.3  [11.6] | 47.9 | 49.1  [7.8] |
| **l-Leu4** | | | | **d-Val4** | | | |
| 43.9 | 49.0  [7.3] | 53.9 | 49.6  [10.9] | 64.6 | 63.7  [7.9] | 45.4 | 43.7  [7.2] |
| **d-Leu5** | | | | **l-Leu5** | | | |
| 76.4 | 73.1  [11.4] | –9.7 | 3.6  [18.3] | 56.5 | 56.7  [7.1] | 10.9 | 8.6  [16.1] |
| **d-Ser6** | | | | **d-Ser6** | | | |
| 131.8 | 127.8  [22.2] | –7.5 | –3.4  [16.7] | 132.7 | 134.4  [18.2] | –1.2 | 1.6  [11.2] |
| **l-Leu7** | | | | **l-Leu7** | | | |
| –86.5 | –82.8  [17.6] | –26.1 | –45.3  [9.7] | –66.1 | –66.9  [10.7] | –25.7 | –18.2  [13.7] |
| **l-Ile8** | | | | **d-Ser8** | | | |
| –150.3 | –137.4  [9.5] | –93 | –86.7  [8.6] | 92.4 | 94.5  [18.4] | 1.5 | –8.8  [15.4] |
|  | | | | **l-Ile9** | | | |
|  |  |  |  | –85.8 | –79.6  [17.6] | 6.9 | –3.2  [12.2] |

*For the C-terminal amino acid the ψ angle refers to the side-chain-backbone angle defined between the N(i)–Cα(i)–C’(i)–OG atoms.*

**Table S13.** Phi/psi torsion angle data of the MD refined orfamide and xantholysin structures.

| **orfamide (10:8)** | | | | **xantholysin (14:8)** | | | |
| --- | --- | --- | --- | --- | --- | --- | --- |
| ***φ***/^o^ | | ***ψ***/^o^ | | ***φ***/^o^ | | ***ψ***/^o^ | |
| **Rep.**  **Struct.** | **MD Average [StDev]** | **Rep.**  **Struct.** | **MD Average [StDev]** | **Rep.**  **Struct.** | **MD Average [StDev]** | **Rep.**  **Struct.** | **MD Average [StDev]** |
|  |  |  |  | **l-Leu1** | | | |
|  |  |  |  | 56.9 | 41.2  [28.2] | 53.5 | 56.5  [21.9] |
|  |  |  |  | **d-Glu2** | | | |
|  |  |  |  | 46.3 | 64.6  [9.5] | 48.7 | 29.3  [9.7] |
|  |  |  |  | **d-Gln3** | | | |
|  |  |  |  | 60.9 | 74.1  [9.9] | 45.3 | 37.0  [11.5] |
|  |  |  |  | **d-Val4** | | | |
|  |  |  |  | 61.5 | 66.6  [10.3] | 54.9 | 46.9  [8.6] |
| **l-Leu1** | | | | **d-Leu5** | | | |
| 58.9 | 47.3  [9.9] | 43.3 | 52.7  [11.7] | 57.1 | 59.7  [8.2] | 44.2 | 45.3  [8.0] |
| **d-Glu2** | | | | **l -Gln6** | | | |
| 63.1 | 63.9  [9.3] | 35.0 | 31.4  [9.8] | 49.8 | 51.2  [7.4] | 64.1 | 49.3  [15.4] |
| **d-*allo*-Thr3** | | | | **d-Ser7** | | | |
| 70.7 | 61.8  [10.4] | 38.5 | 47.5  [9.3] | 48.3 | 60.8  [16.9] | 35.2 | 47.4  [9.8] |
| **d-*allo*-Ile4** | | | | **d -Val8** | | | |
| 66.1 | 64.3  [9.2] | 62.8 | 49.7  [9.2] | 75.7 | 65.2  [9.3] | 60.7 | 48.2  [12.4] |
| **d-Leu5** | | | | **d-Leu9** | | | |
| 55.9 | 66.0  [9.3] | 16.5 | 18.3  [12.0] | 57.6 | 69.0  [8.7] | 22.8 | 21.4  [11.3] |
| **d-Ser6** | | | | **d-Gln10** | | | |
| 83.5 | 71.7  [10.4] | –17.2 | –6.4  [26.4] | 67.6 | 74.5  [11.3] | –4.5 | –9.3  [19.5] |
| **l-Leu7** | | | | **l-Leu11** | | | |
| –86.7 | –88.4 [24.4] | –32.3 | –33.8  [14.2] | –85.5 | –85.0  [22.4] | –58.1 | –40.1  [12.5] |
| **l-Leu8** | | | | **l-Leu12** | | | |
| –82.3 | –89.3  [19.8] | –18.3 | –19.6  [12.2] | –59.9 | –83.1  [13.3] | –19.2 | –13.0  [14.5] |
| **d-Ser9** | | | | **d-Gln13** | | | |
| 79.7 | 76.4  [12.0] | 9.0 | –2.3  [25.1] | 74.6 | 77.5  [10.8] | –28.5 | –20.1  [32.8] |
| **l-Val10** | | | | **l-Ile14** | | | |
| –128.8 | –113.9  [35.4] | 83.5 | 49.3  [63.5]* | –83.8 | –96.0  [30.8] | –9.0 | –9.9  [72.0]* |

*For the C-terminal amino acid the ψ angle refers to the side-chain-backbone angle defined between the N(i)–Cα(i)–C’(i)–OG atoms. *The high standard deviation of these values is the result of exchange between two distinct states, see SI Figure S4.*

**Table S14.** Phi/psi torsion angle data of the MD refined arthrofactin and tanniamide structures.

| **arthrofactin (11:9)** | | | | **tanniamide (12:10)** | | | |
| --- | --- | --- | --- | --- | --- | --- | --- |
| ***φ***/^o^ | | ***ψ***/^o^ | | ***φ***/^o^ | | ***ψ***/^o^ | |
| **Rep.**  **Struct.** | **MD Average [StDev]** | **Rep.**  **Struct.** | **MD Average [StDev]** | **Rep.**  **Struct.** | **MD Average [StDev]** | **Rep.**  **Struct.** | **MD Average [StDev]** |
| **d-Leu1** | | | | **d-Leu1** | | | |
| 53.7 | 60.9  [11.7] | 43.9 | 40.2  [10.5] | 51.8 | 58.9  [9.0] | 41.0 | 40.6  [11.1] |
| **d-Asp2** | | | | **d-Asp2** | | | |
| 60.4 | 65.7  [9.9] | 41.3 | 29.0  [13.6] | 61.7 | 68.3  [10.9] | 25.5 | 26.2  [15.6] |
| **d-*allo*-Thr3** | | | | **d-*allo*-Thr3** | | | |
| 65.5 | 72.7  [17.5] | 54.0 | 50.6  [7.6] | 69.2 | 69.8  [21.2] | 56.7 | 53.6  [9.2] |
| **d-Leu4** | | | | **d-*allo* -Ile4** | | | |
| 51.5 | 54.7  [8.0] | 44.0 | 44.1  [8.6] | 69.2 | 67.7  [11.2] | 27.7 | 30.7  [12.2] |
| **d-Leu5** | | | | **l-Leu5** | | | |
| 69.0 | 65.5  [10.3] | 4.1 | 9.6  [11.5] | 50.2 | 55.0  [7.7] | 22.0 | 13.1  [11.0] |
| **d-Ser6** | | | | **d-Gln6** | | | |
| 62.9 | 65.1  [9.3] | 19.8 | 20.0  [9.8] | 56.5 | 66.3  [10.7] | 23.5 | 19.5  [11.7] |
| **l-Leu7** | | | | **l-Leu7** | | | |
| –80.8 | –72.6  [10.6] | –10.7 | –11.9  [10.9] | –72.9 | –73.6  [12.6] | –4.9 | –17.1  [17.5] |
| **d-Ser8** | | | | **d-Ser8** | | | |
| 90.1 | 77.5  [13.4] | 4.2 | 12.7  [14.3] | 92.9 | 95.6  [25.3] | 16.8 | 22.2  [11.3] |
| **l -Ile9** | | | | **l-Leu9** | | | |
| –77.8 | –91.2  [19.3] | –47.4 | –35.4  [12.6] | –64.9 | –73.2  [10.5] | –12.8 | –9.5  [13.9] |
| **l-Ile10** | | | | **d-Gln10** | | | |
| –143.1 | –142.9  [14.8] | 139.3 | 140.4  [11.5] | 55.2 | 81.1  [15.9] | 24.1 | 4.5  [25.4] |
| **l-Asp11** | | | | **l-Ile11** | | | |
| –69.4 | –71.7  [11.7] | 121.1 | 112.4  [11.8] | –93.5 | –97.4 [27.9] | 115.6 | 129.7  [15.7] |
|  | | | | **l-Glu12** | | | |
|  |  |  |  | –72.3 | –69.5 [18.4] | 114.1 | 120.1  [17.2] |

*For the C-terminal amino acid the ψ angle refers to the side-chain-backbone angle defined between the N(i)–Cα(i)–C’(i)–OG atoms.*

**Table S15.** The simulated average *φ* and *ψ* backbone dihedral angles of the MDN-0066(8:6), viscosin (9:7), orfamide (10:8), arthrofactin (11:9) and tanniamide (12:10) conformations. The depsi bonding residues are highlighted in bold.

| MDN-0066 (8:6) | | viscosin (9:7) | | orfamide (10:8) | | arthrofactin (11:9) | | tanniamide (12:10) | |
| --- | --- | --- | --- | --- | --- | --- | --- | --- | --- |
| d-Leu1 | | l-Leu1 | | l-Leu1 | | d-Leu1 | | d-Leu1 | |
| 59.9 | 38.6 | 49.7 | 51.8 | 47.3 | 52.7 | 60.9 | 40.2 | 58.9 | 40.6 |
| d-Glu2 | | d-Glu2 | | d-Glu2 | | d-Asp2 | | d-Asp2 | |
| 65.8 | 31.3 | 66.6 | 24.3 | 63.9 | 31.4 | 65.7 | 29.0 | 68.3 | 26.2 |
| **d-*allo*-Thr3** | | **d-*allo*-Thr3** | | **d-*allo*-Thr3** | | **d-*allo*-Thr3** | | **d-*allo*-Thr3** | |
| 71.2 | 47.6 | 72.3 | 49.1 | 61.8 | 47.5 | 72.7 | 50.6 | 69.8 | 53.6 |
| l-Leu4 | | d-Val4 | | d-*allo*-Ile4 | | d-Leu4 | | d-*allo*-Ile4 | |
| 49.0 | 49.6 | 63.7 | 43.7 | 64.3 | 49.7 | 54.7 | 44.1 | 67.7 | 30.7 |
| d-Leu5 | | l-Leu5 | | d-Leu5 | | d-Leu5 | | l-Leu5 | |
| 73.1 | 3.6 | 56.7 | 8.6 | 66.0 | 18.3 | 65.5 | 9.6 | 55.0 | 13.1 |
| d-Ser6 | | d-Ser6 | | d-Ser6 | | d-Ser6 | | d -Gln6 | |
| 127.8 | –3.4 | 134.4 | 1.6 | 71.7 | –6.4 | 65.1 | 20.0 | 66.3 | 19.5 |
| l-Leu7 | | l-Leu7 | | l-Leu7 | | l -Leu7 | | l-Leu7 | |
| –82.8 | –45.3 | –66.9 | –18.2 | –88.4 | –33.8 | –72.6 | –11.9 | –73.6 | –17.1 |
| l-Ile8 | | d-Ser8 | | l-Leu8 | | d-Ser8 | | d-Ser8 | |
| –137.4 | –86.7 | 94.5 | –8.8 | –89.3 | –19.6 | 77.5 | 12.7 | 95.6 | 22.2 |
| ***φ*** [^o^] | ***ψ*** [^o^] | l-Ile9 | | d-Ser9 | | l-Ile9 | | l-Leu9 | |
|  | | –79.6 | –3.2 | 76.4 | –2.3 | –91.2 | –35.4 | –73.2 | –9.5 |
|  |  | ***φ*** [^o^] | ***ψ*** [^o^] | l-Val10 | | l-Ile10 | | d-Gln10 | |
|  |  |  | | –113.9 | 49.3 | –142.9 | 140.4 | 81.1 | 4.5 |
|  |  |  |  | ***φ*** [^o^] | ***ψ*** [^o^] | l-Asp11 | | l-Ile11 | |
|  |  |  |  |  | | –71.7 | 112.4 | –97.4 | 129.7 |
|  |  |  |  |  |  | ***φ*** [^o^] | ***ψ*** [^o^] | l-Gln12 | |
|  |  |  |  |  |  |  | | –69.5 | 120.1 |
|  |  |  |  |  |  |  |  | ***φ*** [^o^] | ***ψ*** [^o^] |

*For the C-terminal amino acid the ψ angle refers to the side-chain-backbone angle defined between the N(i)–Cα(i)–C’(i)–OG atoms.*

**Table S16.** Phi/psi torsion angle data of the MD refined putisolvin and entolysin structures.

| **putisolvin (12:4)** | | | | **entolysin (14:5)** | | | |
| --- | --- | --- | --- | --- | --- | --- | --- |
| ***φ***/^o^ | | ***ψ***/^o^ | | ***φ***/^o^ | | ***ψ***/^o^ | |
| **Rep.**  **Struct.** | **MD Average [StDev]** | **Rep.**  **Struct.** | **MD Average [StDev]** | **Rep.**  **Struct.** | **MD Average [StDev]** | **Rep.**  **Struct.** | **MD Average [StDev]** |
|  | | | | **l-Leu1** | | | |
|  |  |  |  | 70.2 | 51.5  [11.2] | 45.6 | 44.8  [12.8] |
| **l-Leu1** | | | | **d-Glu2** | | | |
| 53.5 | 51.8  [7.7] | 41.6 | 40.5  [10.1] | 73.5 | 63.7  [10.3] | 23.7 | 26.8  [10.5] |
| **d-Glu2** | | | | **d-Gln3** | | | |
| 79.8 | 68.2  [10.4] | 18.9 | 31.5  [11.6] | 65.1 | 74.9  [11.8] | 45.4 | 36.2  [10.8] |
| **d-Leu3** | | | | **d-Val4** | | | |
| 77.3 | 74.9  [13.0] | 40.4 | 40.3  [9.1] | 63.2 | 68.4  [11.6] | 33.3 | 37.4  [9.2] |
| **l-Leu4** | | | | **d-Leu5** | | | |
| 53.5 | 51.5  [8.2] | 43.9 | 51.2  [8.7] | 70.2 | 61.3  [8.8] | 29.6 | 34.8  [9.1] |
| **d-Gln5** | | | | **d-Gln6** | | | |
| 75.3 | 63.0  [8.4] | 27.2 | 32.8  [8.9] | 67.5 | 65.9  [9.5] | 35.1 | 34.8  [9.8] |
| **d-Ser6** | | | | **d-Val7** | | | |
| 55.8 | 67.5  [9.1] | 50.4 | 40.9  [8.8] | 72.4 | 67.1  [9.9] | 42.1 | 45.6  [8.3] |
| **d-Val7** | | | | **d-Leu8** | | | |
| 64.4 | 66.6  [8.6] | 47.6 | 39.7  [9.2] | 68.6 | 62.0  [8.6] | 35.9 | 34.4  [10.6] |
| **d-Leu8** | | | | **d-Gln9** | | | |
| 53.0 | 63.6  [8.5] | 33.5 | 23.1  [13.0] | 57.2 | 72.9  [10.9] | 46.2 | 24.1  [19.6] |
| **d-Ser9** | | | | **d-Ser10** | | | |
| 88.4 | 92.4  [18.0] | –9.5 | –1.5  [15.0] | 58.3 | 78.7  [24.9] | 67.9 | 62.3  [8.5] |
| **l-Leu10** | | | | **l-Val11** | | | |
| –64.6 | –77.6  [16.5] | –38.8 | –35.7  [14.3] | –63.0 | –58.6  [9.1] | 158.6 | 155.1  [8.5] |
| **l-Val11** | | | | **l-Leu12** | | | |
| –98.2 | –102.1  [17.8] | –43.1 | –40.7  [10.7] | 55.3 | 54.2  [7.1] | 34.9 | 31.2  [9.4] |
| **l-Ser12** | | | | **l-Ser13** | | | |
| –150.4 | –139.9  [12.8] | 13.3 | 2.9  [22.0] | 51.0 | 60.9  [7.9] | 31.1 | 22.4  [14.5] |
|  | | | | **l-Ile14** | | | |
|  |  |  |  | –124.4 | –123.2  [19.5] | –66.5 | –36.0  [43.4]* |

*For the C-terminal amino acid the ψ angle refers to the side-chain-backbone angle defined between the N(i)–Cα(i)–C’(i)–OG atoms. *The high standard deviation of these values is the result of exchange between two distinct states, see SI Figure S4.*

## **4.2. PROCHECK analysis of the CNS vs MD refined CLiP conformations**


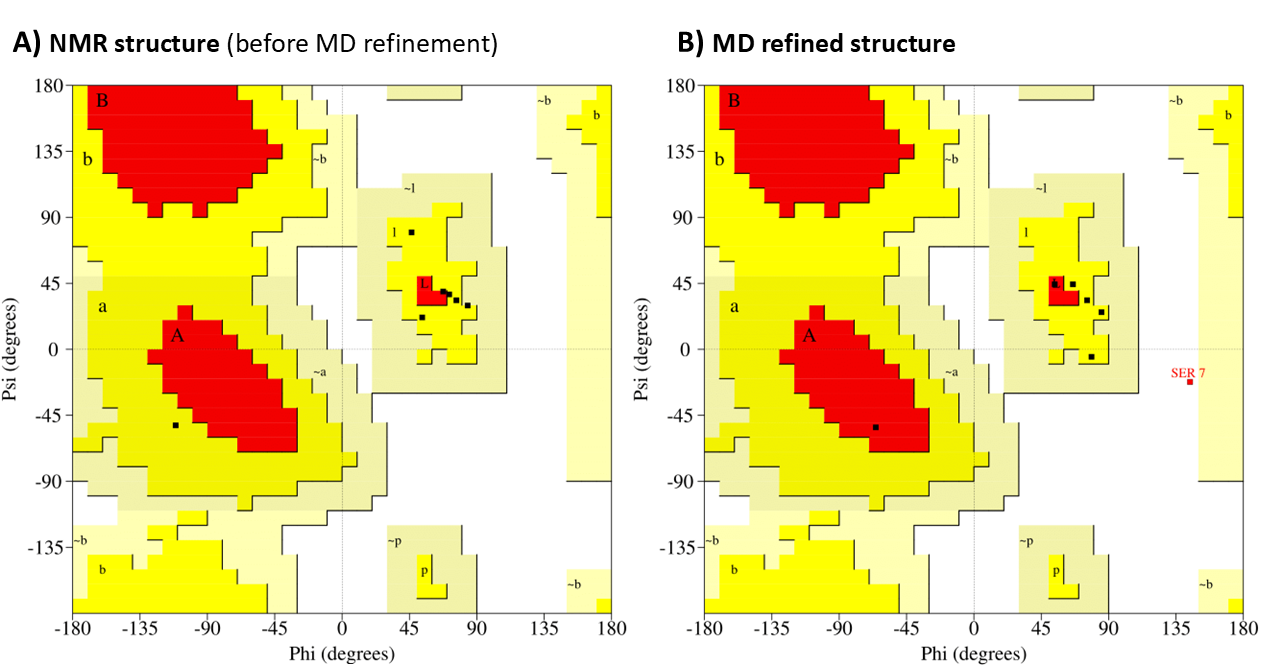


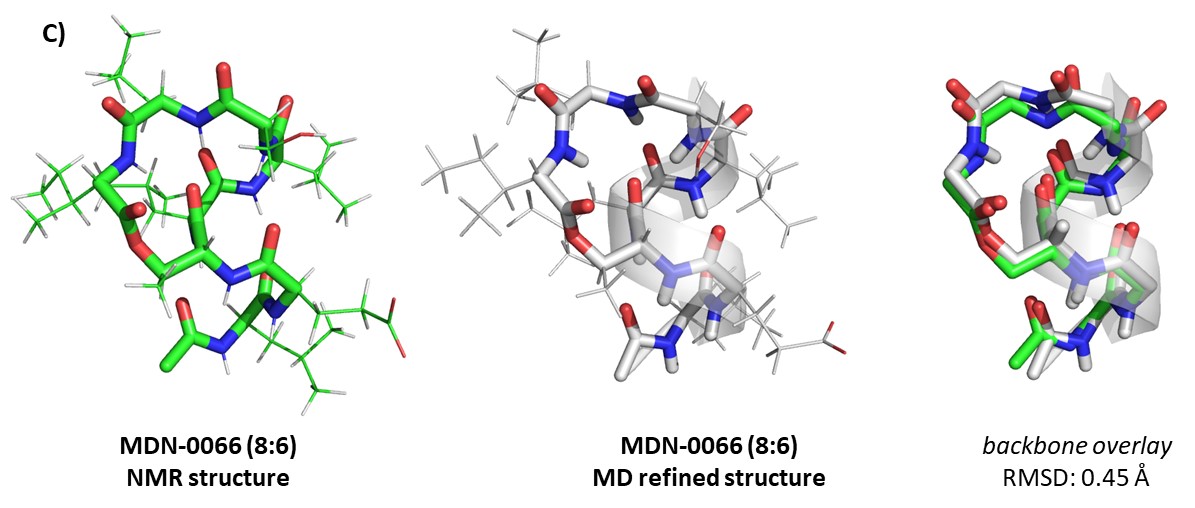


**Figure S5.** PROCHECK Ramachandran plots of **MDN-0066 (8:6)** **A)** NMR structure (before MD refinement) **B)** MD refined structure used for further analysis and comparison. Residues in disallowed or generously allowed regions are indicated in red. Note that for the PROCHECK analysis the acyl chain was considered as the first residue thus all amino acid residue numbers are shifted accordingly. **C)** Visual comparison of the structures with the mutual backbone RMSD. The acyl chain residue was omitted from the RMSD calculation and visualization.

**Table S17.** PROCHECK Ramachandran plot statistics of **MDN-0066 (8:6)**.

| **MDN-0066 (8:6)** | NMR structure | | MD refined structure | |
| --- | --- | --- | --- | --- |
| Residues in most favored regions [A,B,L] | 1 | 14.3% | 2 | 28.6% |
| Residues in additional allowed regions [a, b,l,p] | 6 | 85.7% | 4 | 57.1% |
| Residues in generously allowed regions [~a,~b,~l,~p] | 0 | 0.0% | 0 | 0.0% |
| Residues in disallowed regions | 0 | 0.0% | 1: d-Ser6 | 14.3% |
| Number of non-glycine and non-proline residues | 7 | 100% | 7 | 100% |
| Number of end residues (excl. Gly and Pro) | 2 |  | 2 |  |
| Total # of residues identified by PROCHECK | 9 |  | 9 |  |

*The C-terminal l-Ile is omitted from the Ramachandran maps and not considered for these statistics.*


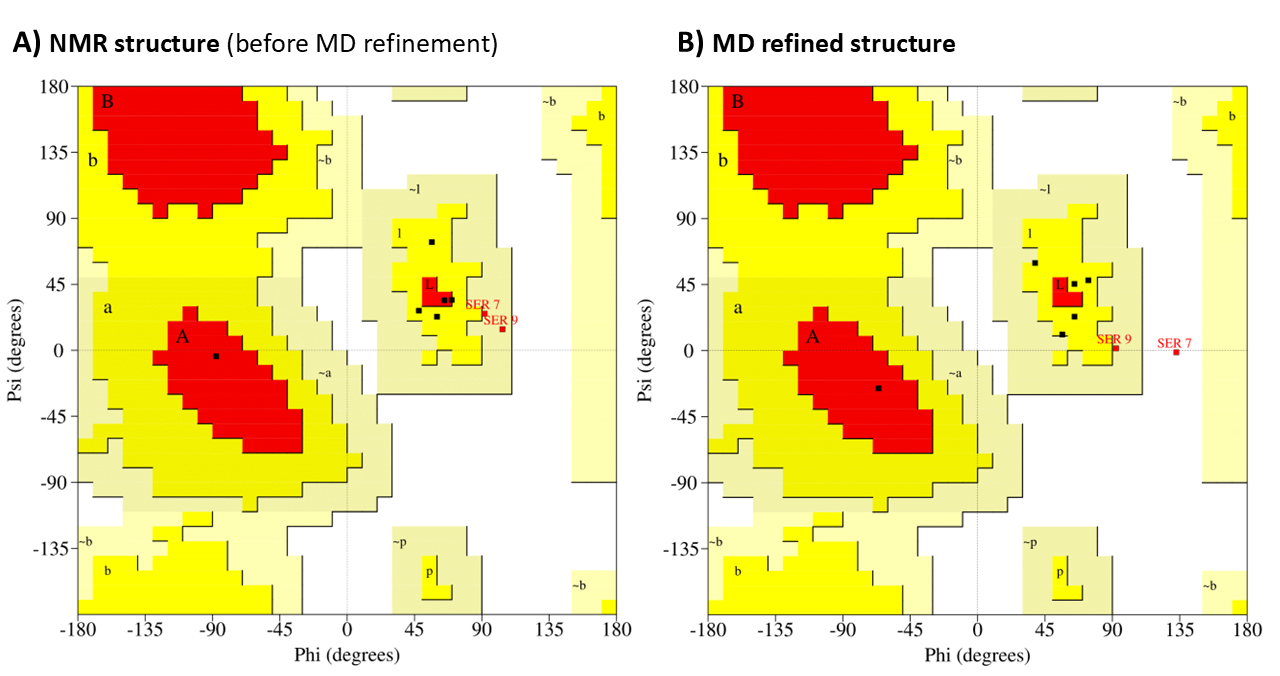


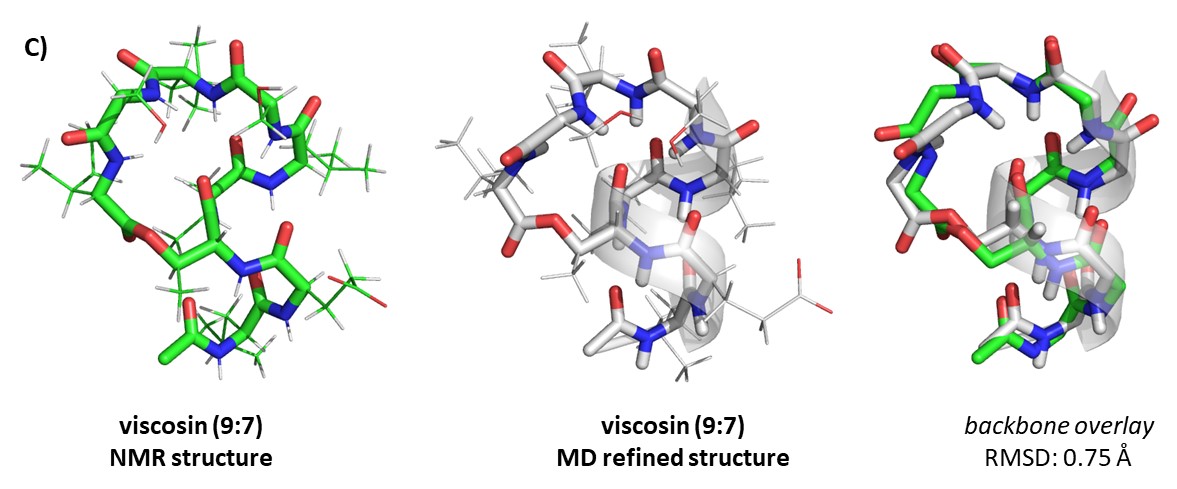


**Figure S6.** PROCHECK Ramachandran plots of **viscosin (9:7)** **A)** NMR structure (before MD refinement) **B)** MD refined structure used for further analysis and comparison. Residues in disallowed or generously allowed regions are indicated in red. Note that for the PROCHECK analysis the acyl chain was considered as the first residue thus all amino acid residue numbers are shifted accordingly. **C)** Visual comparison of the structures with the mutual backbone RMSD. The acyl chain residue was omitted from the RMSD calculation and visualization.

**Table S18.** PROCHECK Ramachandran plot statistics of **viscosin (9:7)**.

| **viscosin (9:7)** | NMR structure | | MD refined structure | |
| --- | --- | --- | --- | --- |
| Residues in most favored regions [A,B,L] | 3 | 37.5% | 1 | 12.5% |
| Residues in additional allowed regions [a, b,l,p] | 3 | 37.5% | 5 | 62.5% |
| Residues in generously allowed regions [~a,~b,~l,~p] | 2 | 25.0% | 1 (d-Ser8) | 12.5% |
| Residues in disallowed regions | 0 | 0.0% | 1 (d-Ser6) | 12.5% |
| Number of non-glycine and non-proline residues | 8 | 100% | 8 | 100% |
| Number of end residues (excl. Gly and Pro) | 2 |  | 2 |  |
| Total # of residues identified by PROCHECK | 10 |  | 10 |  |

*The C-terminal residue l-Ile is omitted from the Ramachandran maps and not considered for these statistics.*


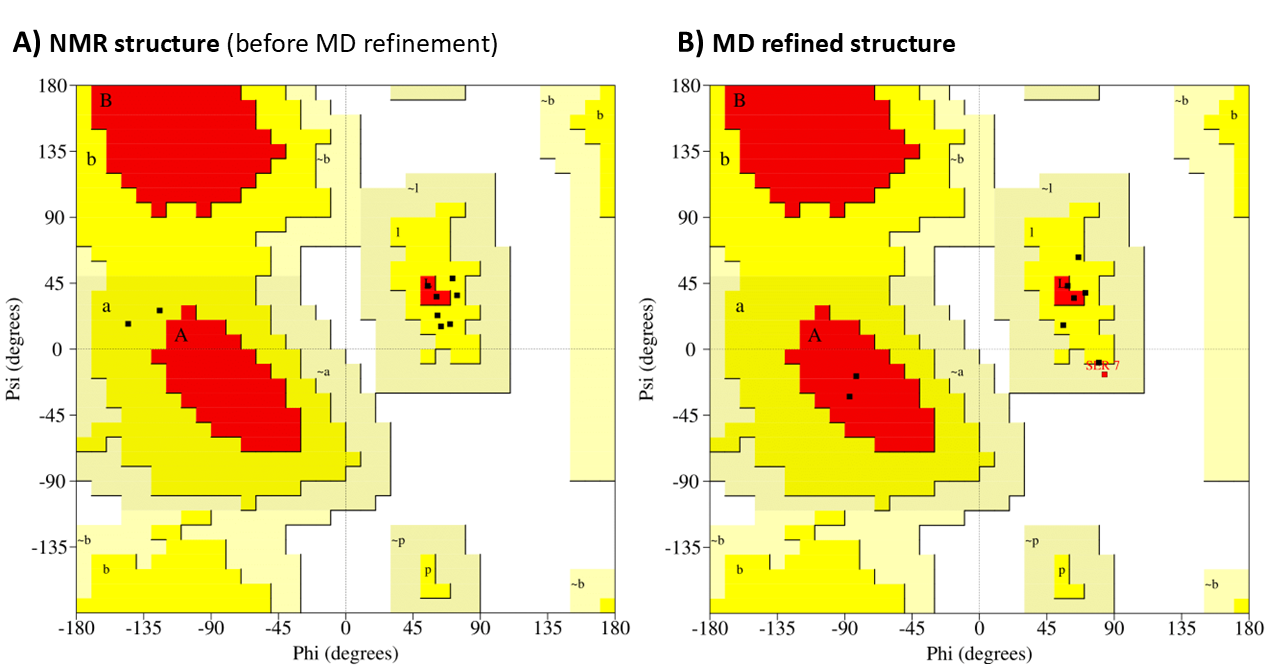


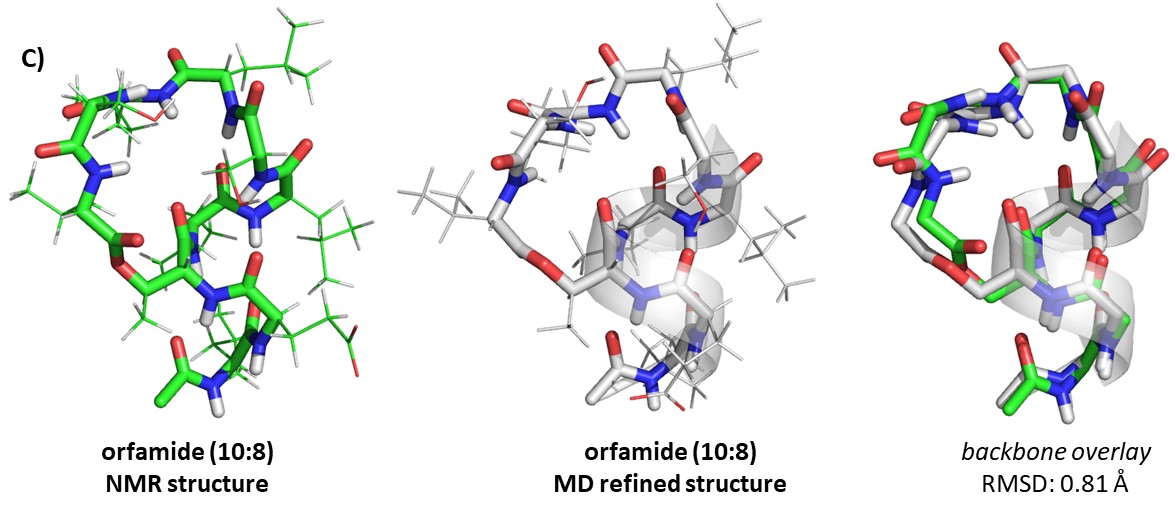


**Figure S7.** PROCHECK Ramachandran plots of **orfamide (10:8)** **A)** NMR structure (before MD refinement) **B)** MD refined structure used for further analysis and comparison. Residues in disallowed or generously allowed regions are indicated in red. Note that for the PROCHECK analysis the acyl chain was considered as the first residue thus all amino acid residue numbers are shifted accordingly. **C)** Visual comparison of the structures with the mutual backbone RMSD. The acyl chain residue was omitted from the RMSD calculation and visualization.

**Table S19.** PROCHECK Ramachandran plot statistics of **orfamide (10:8)**.

| **orfamide (10:8)** | NMR structure | | MD refined structure | |
| --- | --- | --- | --- | --- |
| Residues in most favored regions [A,B,L] | 2 | 22.2% | 4 | 44.4% |
| Residues in additional allowed regions [a, b,l,p] | 7 | 77.8% | 4 | 44.4% |
| Residues in generously allowed regions [~a,~b,~l,~p] | 0 | 0% | 1 (d-Ser6) | 11.1% |
| Residues in disallowed regions | 0 | 0% | 0 | 0% |
| Number of non-glycine and non-proline residues | 9 | 100% | 9 | 100% |
| Number of end residues (excl. Gly and Pro) | 2 |  | 2 |  |
| Total # of residues identified by PROCHECK | 11 |  | 11 |  |

*The C-terminal residue l-Val is omitted from the Ramachandran maps and not considered for these statistics.*

**
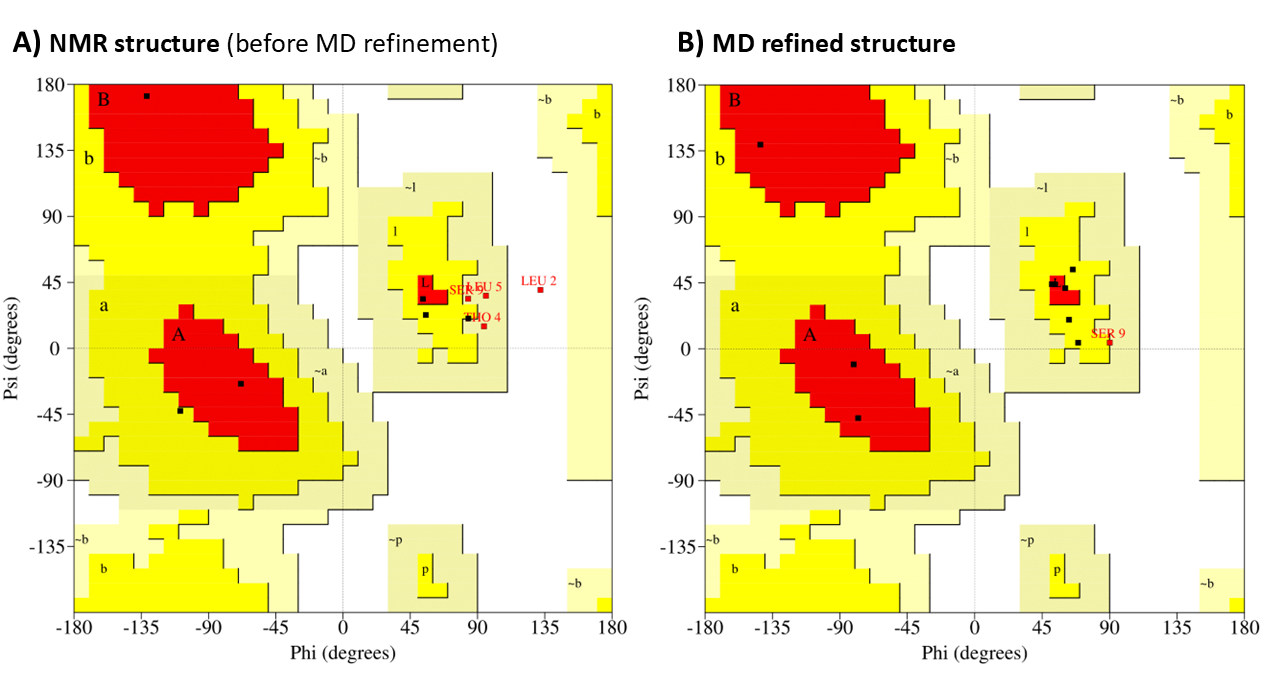
**


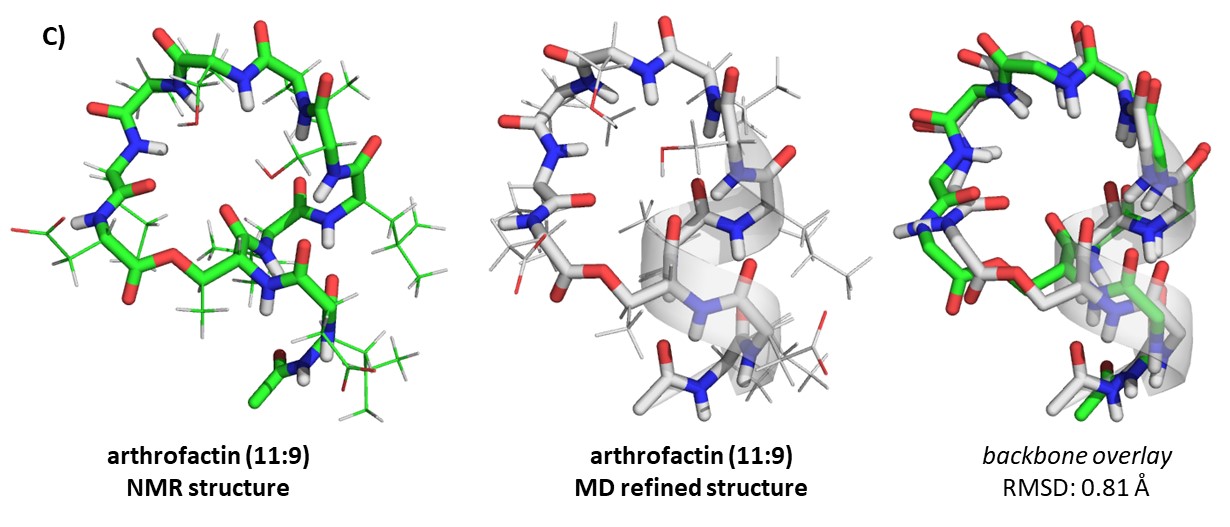


**Figure S8.** PROCHECK Ramachandran plots of **arthrofactin (11:9)** **A)** NMR structure (before MD refinement) **B)** MD refined structure used for further analysis and comparison. Residues in disallowed or generously allowed regions are indicated in red. Note that for the PROCHECK analysis the acyl chain was considered as the first residue thus all amino acid residue numbers are shifted accordingly. **C)** Visual comparison of the structures with the mutual backbone RMSD. The acyl chain residue was omitted from the RMSD calculation and visualization.

**Table S20.** PROCHECK Ramachandran plot statistics of **arthrofactin (11:9)**.

| **arthrofactin (11:9)** | NMR structure | | MD refined structure | |
| --- | --- | --- | --- | --- |
| Residues in most favored regions [A,B,L] | 3 | 30.0% | 5 | 50.0% |
| Residues in additional allowed regions [a, b,l,p] | 3 | 30.0% | 4 | 40.0% |
| Residues in generously allowed regions [~a,~b,~l,~p] | 3 | 10.0% | 1 (d-Ser8) | 10.0% |
| Residues in disallowed regions | 1 | 0% | 0 | 0% |
| Number of non-glycine and non-proline residues | 10 | 100% | 10 | 100% |
| Number of end residues (excl. Gly and Pro) | 2 |  | 2 |  |
| Total # of residues identified by PROCHECK | 12 |  | 12 |  |

*The C-terminal residue l-Asp is omitted from the Ramachandran maps and not considered for these statistics.*


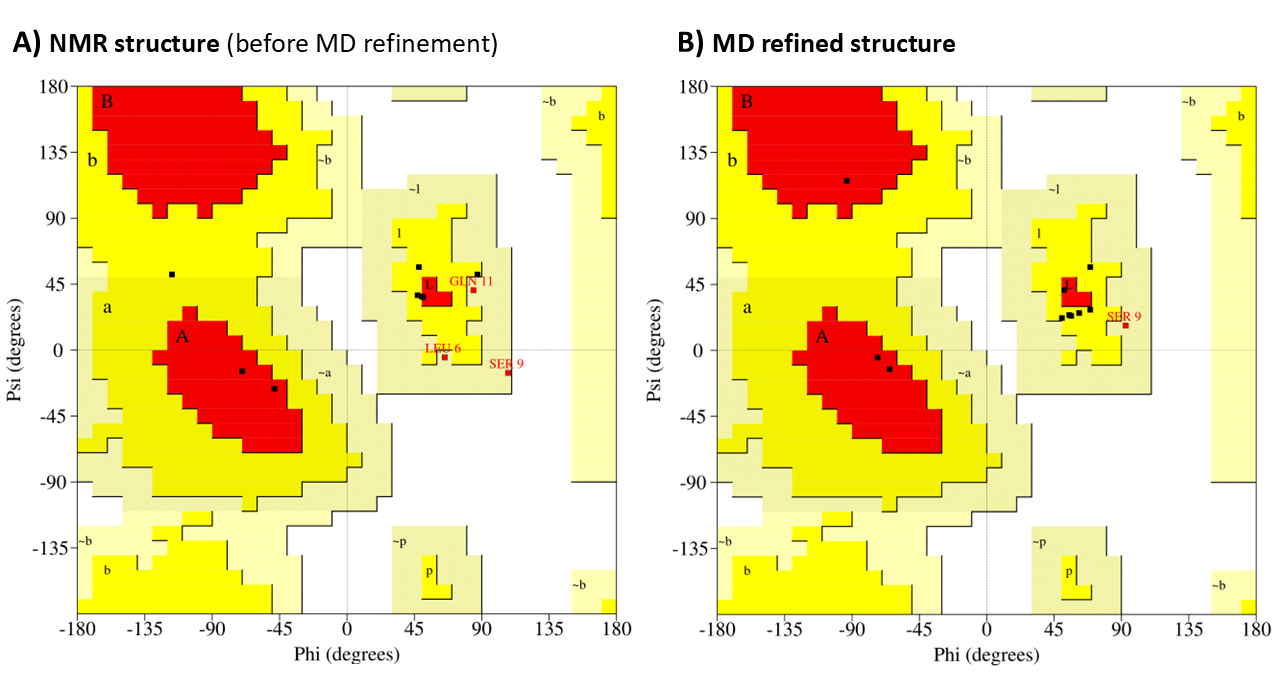


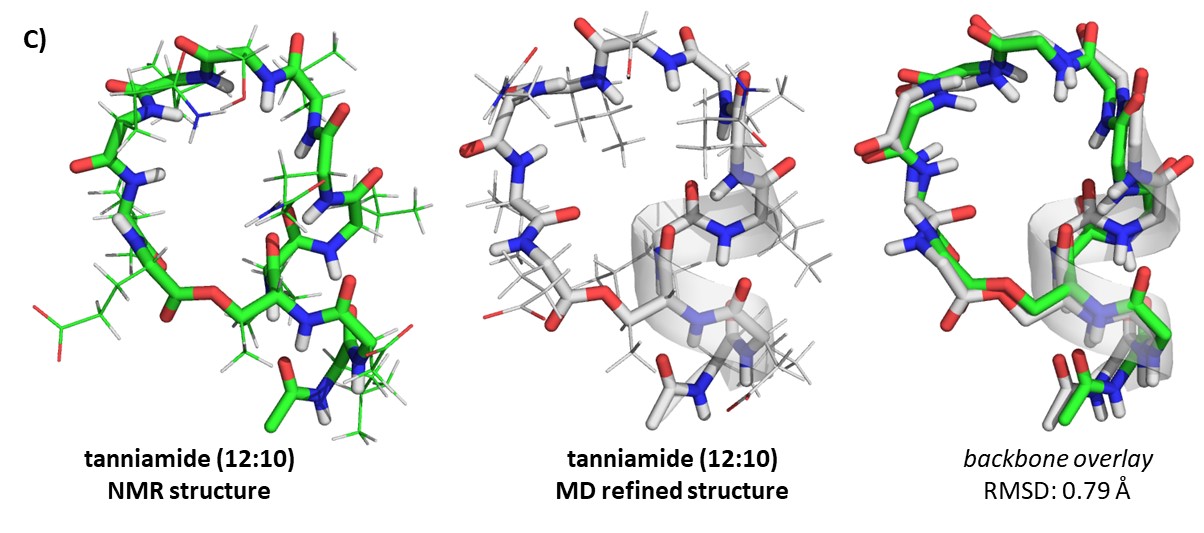


**Figure S9.** PROCHECK Ramachandran plots of **tanniamide (12:10)** **A)** NMR structure (before MD refinement) **B)** MD refined structure used for further analysis and comparison. Residues in disallowed or generously allowed regions are indicated in red. Note that for the PROCHECK analysis the acyl chain was considered as the first residue thus all amino acid residue numbers are shifted accordingly. **C)** Visual comparison of the structures with the mutual backbone RMSD. The acyl chain residue was omitted from the RMSD calculation and visualization.

**Table S21.** PROCHECK Ramachandran plot statistics of **tanniamide (12:10)**.

| **tanniamide (12:10)** | NMR structure | | MD refined structure | |
| --- | --- | --- | --- | --- |
| Residues in most favored regions [A,B,L] | 4 | 36.4% | 4 | 36.4% |
| Residues in additional allowed regions [a, b,l,p] | 4 | 36.4% | 6 | 54.5% |
| Residues in generously allowed regions [~a,~b,~l,~p] | 3 | 27.3% | 1 (d-Ser8) | 9.1% |
| Residues in disallowed regions | 0 | 0.0% | 0 | 0.0% |
| Number of non-glycine and non-proline residues | 11 | 100% | 11 | 100% |
| Number of end residues (excl. Gly and Pro) | 2 |  | 2 |  |
| Total # of residues identified by PROCHECK | 13 |  | 13 |  |

*The C-terminal residue l-Glu is omitted from the Ramachandran maps and not considered for these statistics.*


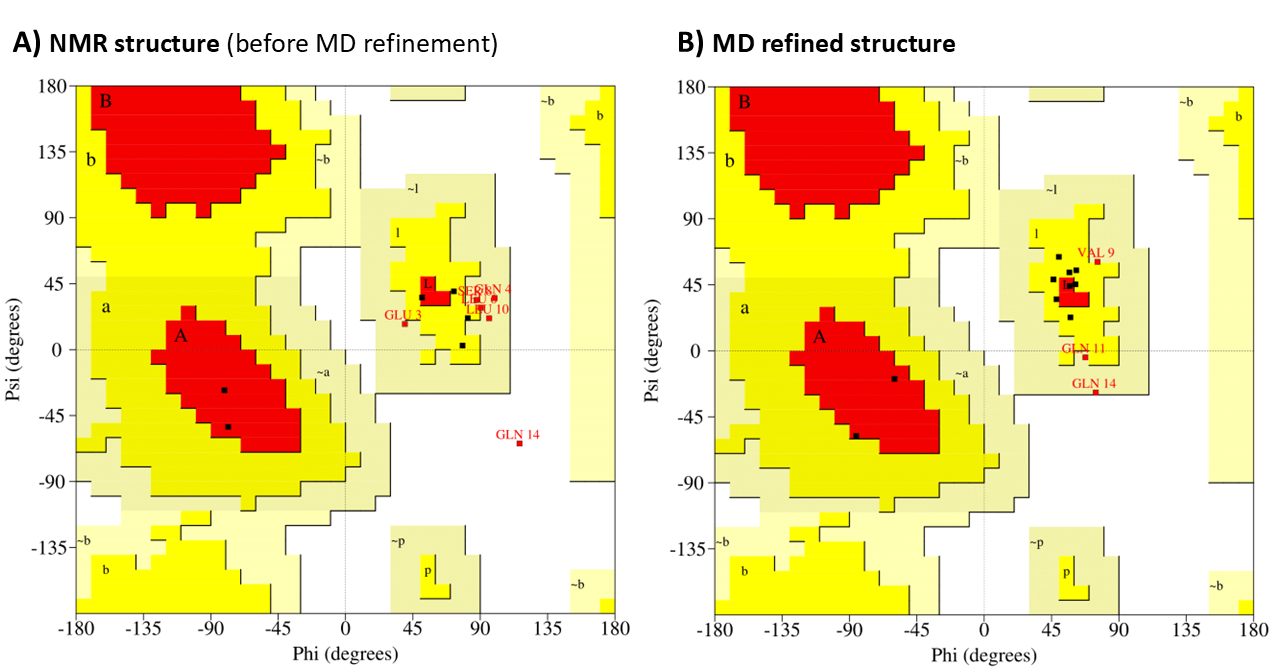


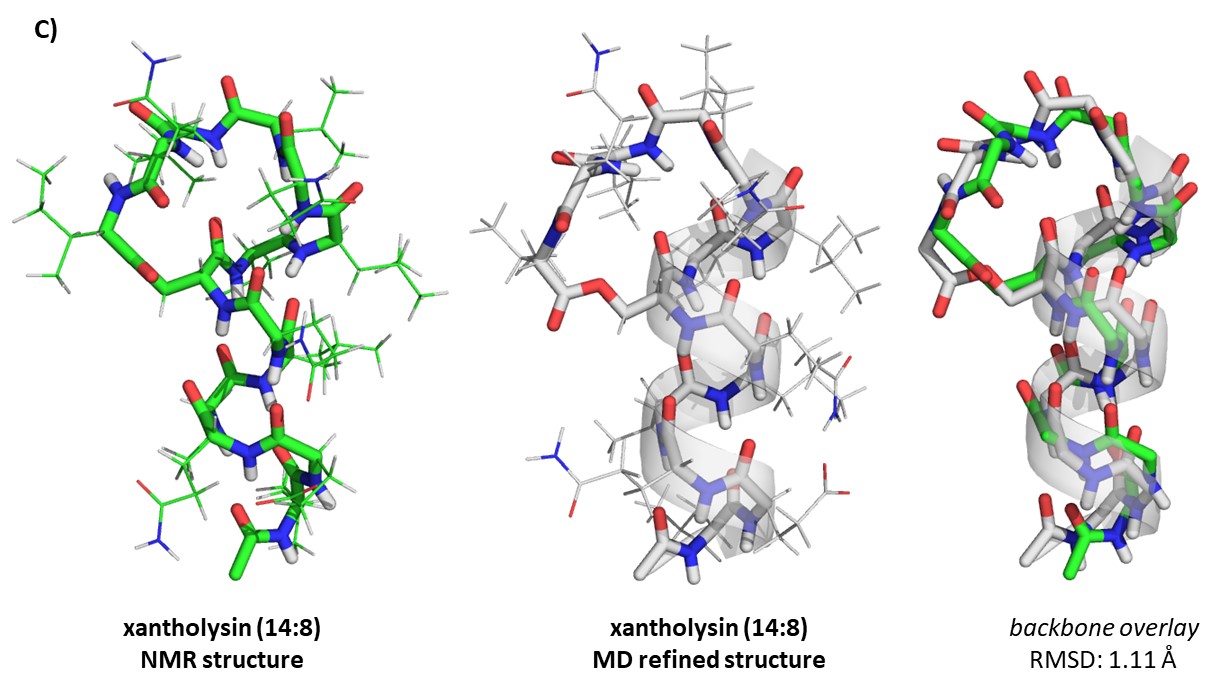


**Figure S10.** PROCHECK Ramachandran plots of **xantholysin (14:8)** **A)** NMR structure **B)** MD refined structure used for further analysis and comparison. Residues in disallowed or generously allowed regions are indicated in red. Note that for the PROCHECK analysis the acyl chain was considered as the first residue thus all amino acid residue numbers are shifted accordingly. **C)** Visual comparison of the structures with the mutual backbone RMSD. The acyl chain residue was omitted from the RMSD calculation and visualization.

**Table S22.** PROCHECK Ramachandran plot statistics of **xantholysin (14:8)**.

| **xantholysin (14:8)** | NMR structure | | MD refined structure | |
| --- | --- | --- | --- | --- |
| Residues in most favored regions [A,B,L] | 3 | 25.0% | 3 | 23.1% |
| Residues in additional allowed regions [a, b,l,p] | 3 | 25.0% | 7 | 53.8% |
| Residues in generously allowed regions [~a,~b,~l,~p] | 5 | 41.7% | 3 | 23.1% |
| Residues in disallowed regions | 1 | 8.3% | 0 | 0% |
| Number of non-glycine and non-proline residues | 12 | 100% | 13 | 100% |
| Number of end residues (excl. Gly and Pro) | 2 |  | 2 |  |
| Total # of residues identified by PROCHECK | 14 |  | 15 |  |

*The C-terminal residue l-Ile is omitted from the Ramachandran maps as well as l-Leu2 in case of the NMR structure and not considered for these statistics.*


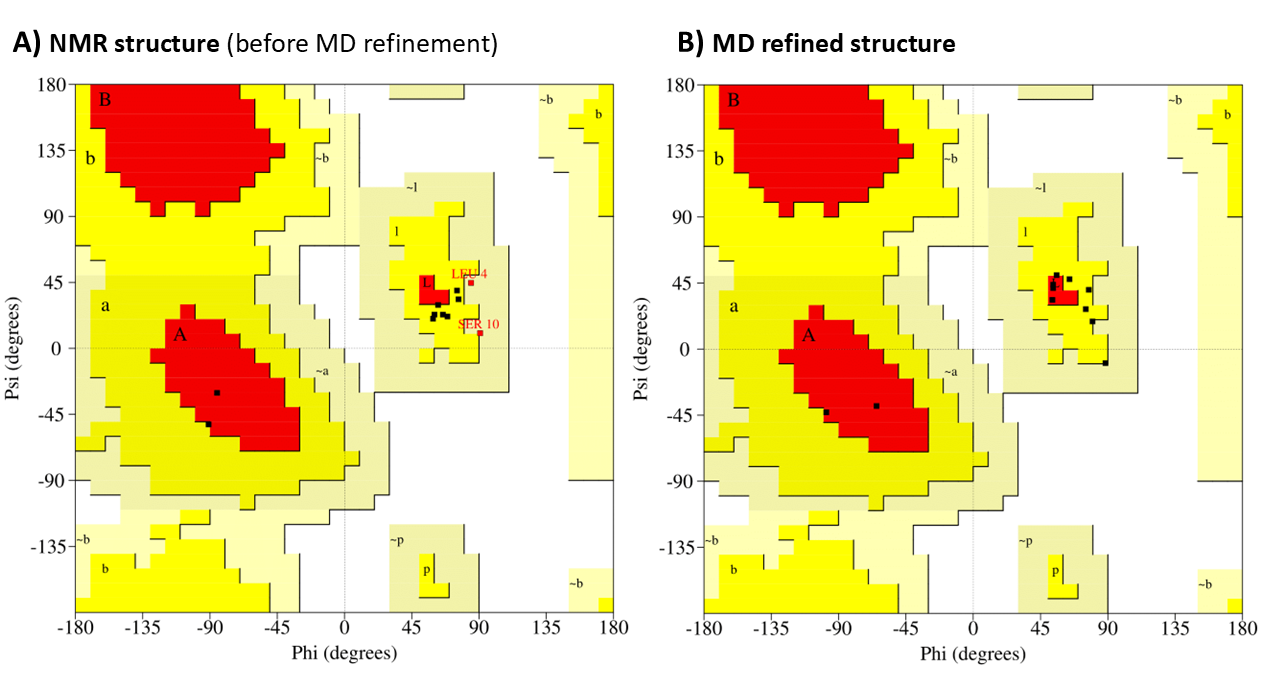


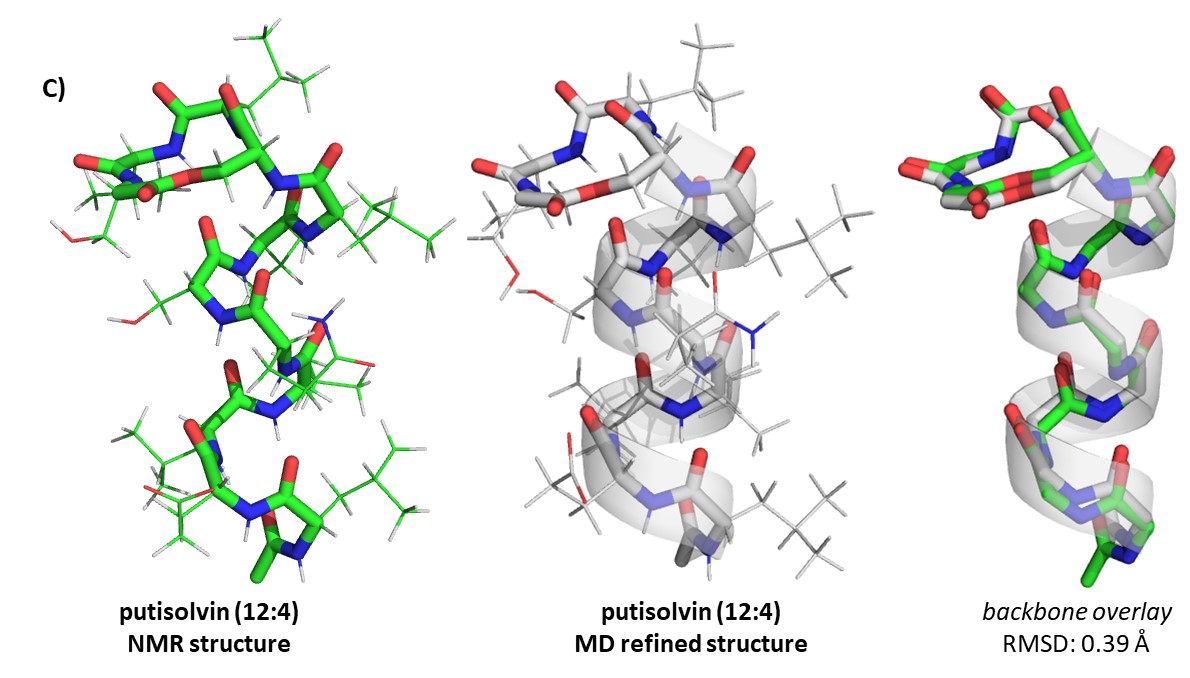


**Figure S11.** PROCHECK Ramachandran plots of **putisolvin (12:4)** **A)** NMR structure (before MD refinement) **B)** MD refined structure used for further analysis and comparison. Residues in disallowed or generously allowed regions are indicated in red. Note that for the PROCHECK analysis the acyl chain was considered as the first residue thus all amino acid residue numbers are shifted accordingly. **C)** Visual comparison of the structures with the mutual backbone RMSD. The acyl chain residue was omitted from the RMSD calculation and visualization.

**Table S23.** PROCHECK Ramachandran plot statistics of **putisolvin (12:4)**.

| **putisolvin (12:4)** | NMR structure | | MD refined structure | |
| --- | --- | --- | --- | --- |
| Residues in most favored regions [A,B,L] | 1 | 9.1% | 5 | 45.5% |
| Residues in additional allowed regions [a, b,l,p] | 8 | 72.7% | 6 | 54.5% |
| Residues in generously allowed regions [~a,~b,~l,~p] | 2 | 18.2% | 0 | 0% |
| Residues in disallowed regions | 0 | 0% | 0 | 0% |
| Number of non-glycine and non-proline residues | 11 | 100% | 11 | 100% |
| Number of end residues (excl. Gly and Pro) | 2 |  | 2 |  |
| Total # of residues identified by PROCHECK | 13 |  | 13 |  |

*The C-terminal residue l-Ser is omitted from the Ramachandran maps and is not considered for these statistics.*


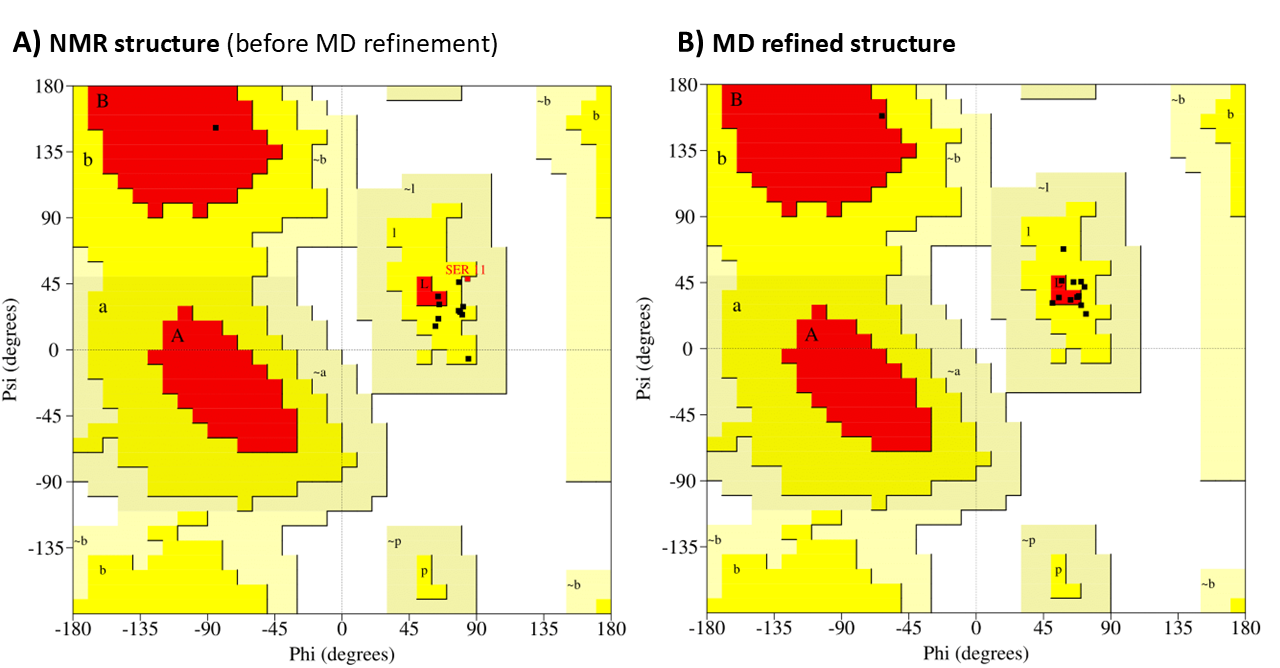


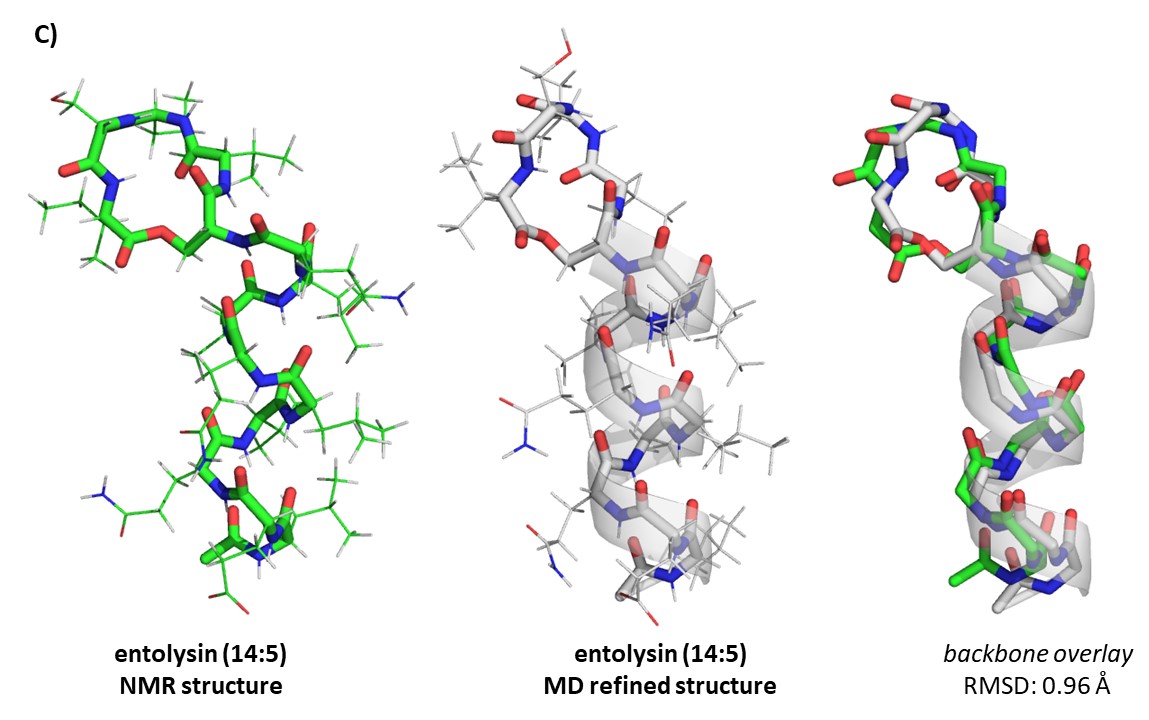


**Figure S12.** PROCHECK Ramachandran plots of **entolysin (14:5)** **A)** NMR structure (before MD refinement) **B)** MD refined structure used for further analysis and comparison. Residues in disallowed or generously allowed regions are indicated in red. Note that for the PROCHECK analysis the acyl chain was considered as the first residue thus all amino acid residue numbers are shifted accordingly. **C)** Visual comparison of the structures with the mutual backbone RMSD. The acyl chain residue was omitted from the RMSD calculation and visualization.

**Table S24.** PROCHECK Ramachandran plot statistics of **entolysin (14:5)**.

| **entolysin (14:5)** | NMR structure | | MD refined structure | |
| --- | --- | --- | --- | --- |
| Residues in most favored regions [A,B,L] | 3 | 25.0% | 7 | 53.8% |
| Residues in additional allowed regions [a, b,l,p] | 8 | 66.7% | 6 | 46.2% |
| Residues in generously allowed regions [~a,~b,~l,~p] | 1 | 8.3% | 0 | 0% |
| Residues in disallowed regions | 0 | 0% | 0 | 0% |
| Number of non-glycine and non-proline residues | 12 | 100% | 13 | 100% |
| Number of end residues (excl. Gly and Pro) | 2 |  | 2 |  |
| Total # of residues identified by PROCHECK | 14 |  | 15 |  |

*The C-terminal residue l-Ile are omitted from the Ramachandran maps as well as l-Leu2 in case of the NMR structure maps and not considered for these statistics.*

## **4.3. Determination of the angle positioning the macrocycle with respect to catch-pole helices**

To quantify the relative orientation between the macrocycle and the helical segment in the three-dimensional structures of CLiPs, we employed the measure inertia command in UCSF ChimeraX^[106]^  (version 1.9). This method fits an ellipsoid to a selected group of atoms and returns three orthogonal principal axes corresponding to the directions of maximal, intermediate, and minimal atomic positional variance. For the macrocycle, the axis with the smallest principal moment (shortest axis) was interpreted as the normal vector to the plane of the macrocycle. For the helical segment, the axis with the largest principal moment (longest axis) was taken as the helical axis. The angle between these two vectors was then calculated using the arccosine of their normalized dot product, providing a quantitative measure of the tilt between the macrocyclic plane and the helix. (SI Figure S13) It is interesting to remark that amongst catch-pole helices putisolvin has a remarkably different macrocycle orientation. Representing the only case with *m*=4 in the current set (all other CLiPs in Figure S13 have *m*=5) the number of residues appears decisive in the drastic change in orientation compared to entolysin (as well as tolaasin and pentorfamide). Indeed, comparing entolysin and putisolvin the first four macrocycle residues have identical amphipathic and configurational character in both CLiPs (sLVS vs. sVLS, s indicating d-Ser) while the added degrees of torsion angle freedom afforded by the extra l-Ile in entolysin and shuffling of the β-branched valine residue combine to impose an almost perpendicular reorientation of the macrocycle backbones with respect to the main helical axis between them.


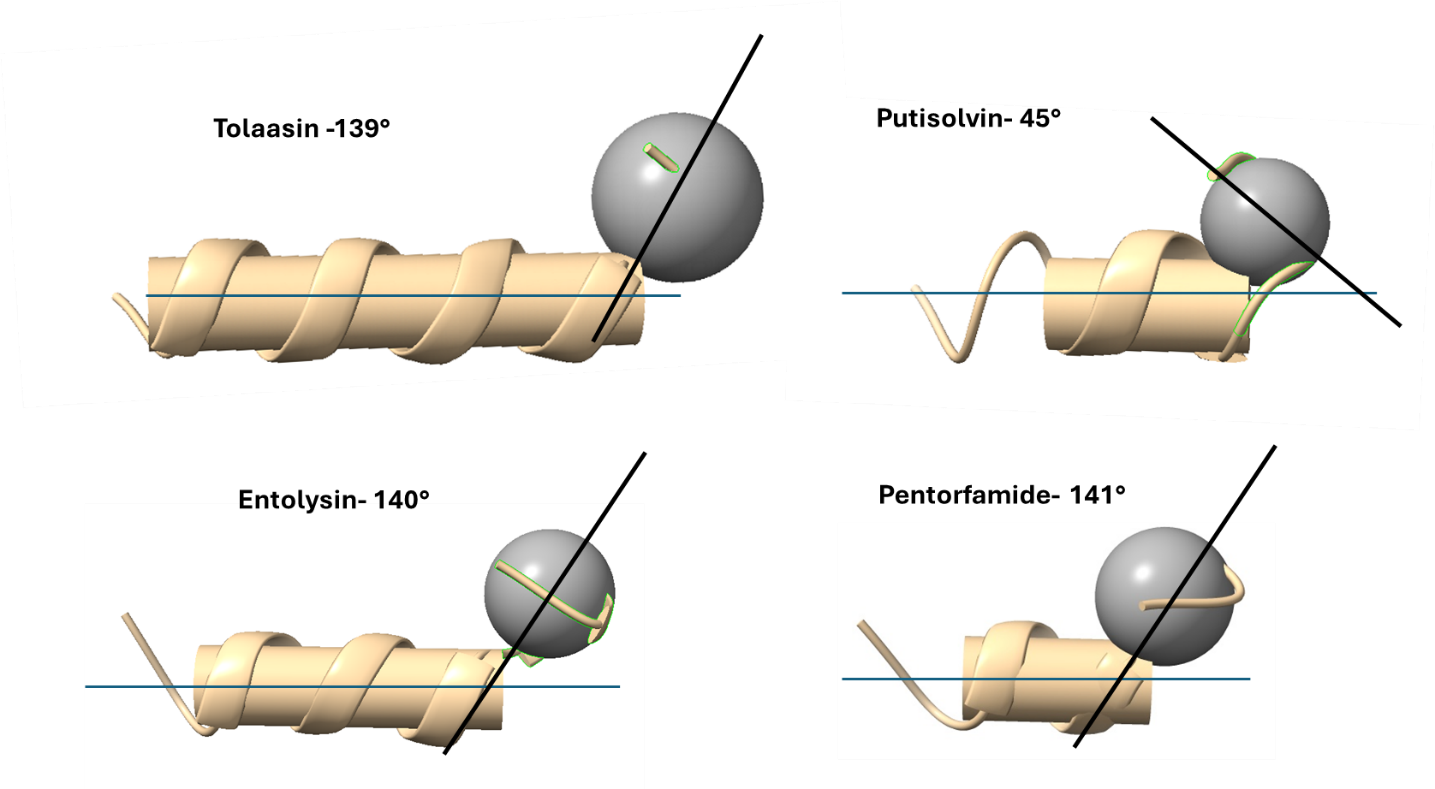


**Figure S13.** Illustrating the orientation of catch-pole macrocycles with respect to the helical axis. The tolaasin structure was obtained from the work of Jourdan *et al.*^[78]^

## **4.4. Surface property calculations and acyl chain reorientations**

The 3D structures of the CLiPs were used as the basis for generating molecular surface property maps, see Figure 4 in the main manuscript. Molecular lipophilicity potential (MLP) surfaces were calculated and visualized using UCSF ChimeraX^[106]^  (version 1.9) with the built-in mlp function. Each CLiP model, including its acyl chain, was processed to display the spatial distribution of lipophilic and polar surface regions, with lipophilic areas represented in gold and polar regions in cyan/white. default settings). Electrostatic surface potential maps were computed using PyMOL (Schrödinger, LLC, version 3.1.3ch), employing the built-in APBS Electrostatics plugin. Charges and radii were assigned using the PDB2PQR tool with the AMBER force field parameters. The resulting electrostatic potentials were mapped onto the molecular surface and colored according to Coulombic potential (red: negative; blue: positive). Only the lipophilic face was visualized for electrostatics, as the opposing surface showed minimal variation and provided limited additional insight.

Using alternative surface representation here we also show the reorientation of the acyl chain during the simulation with respect to the peptide backbone. (SI Figure S14) In the membrane-associated state the acyl chain is restricted to the lipophilic site of the CLiP structure throughout the simulation to interact with the DPC micelle.


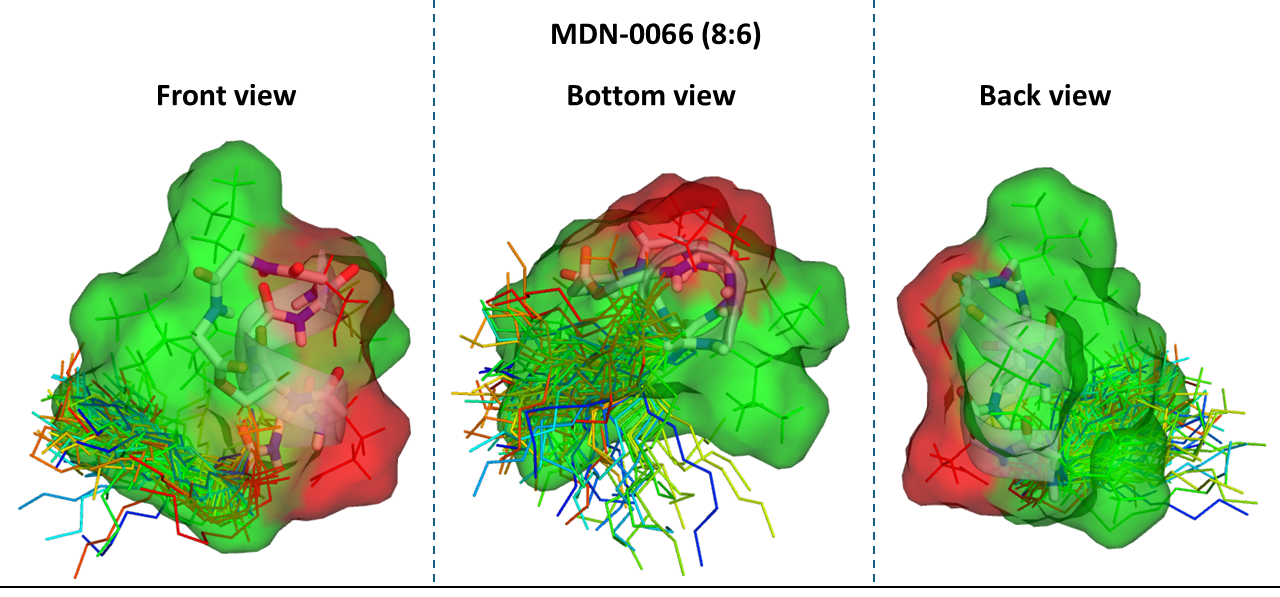


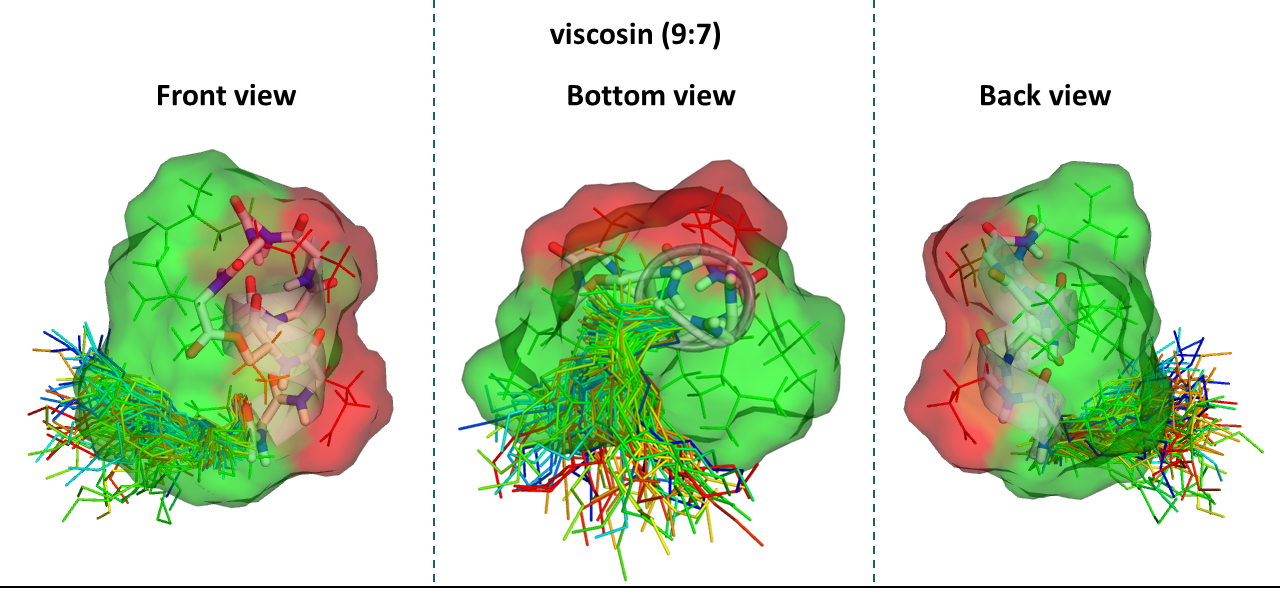


**Figure S14.** During its interaction with the DPC micelle the CLiP acyl chain is restricted to the hydrophobic site (green) of the peptide in the molecular dynamics trajectories. To illustrate this for each CLiP, 100 acyl chains are shown in multiple colors sampled uniformly from the last 50 ns of the corresponding trajectory after N-terminal alignment of the peptide backbones. For clarity, the first selected CLiP structure is displayed only and hydrogen atoms are omitted from both the DPC molecules and the peptide acyl chains.


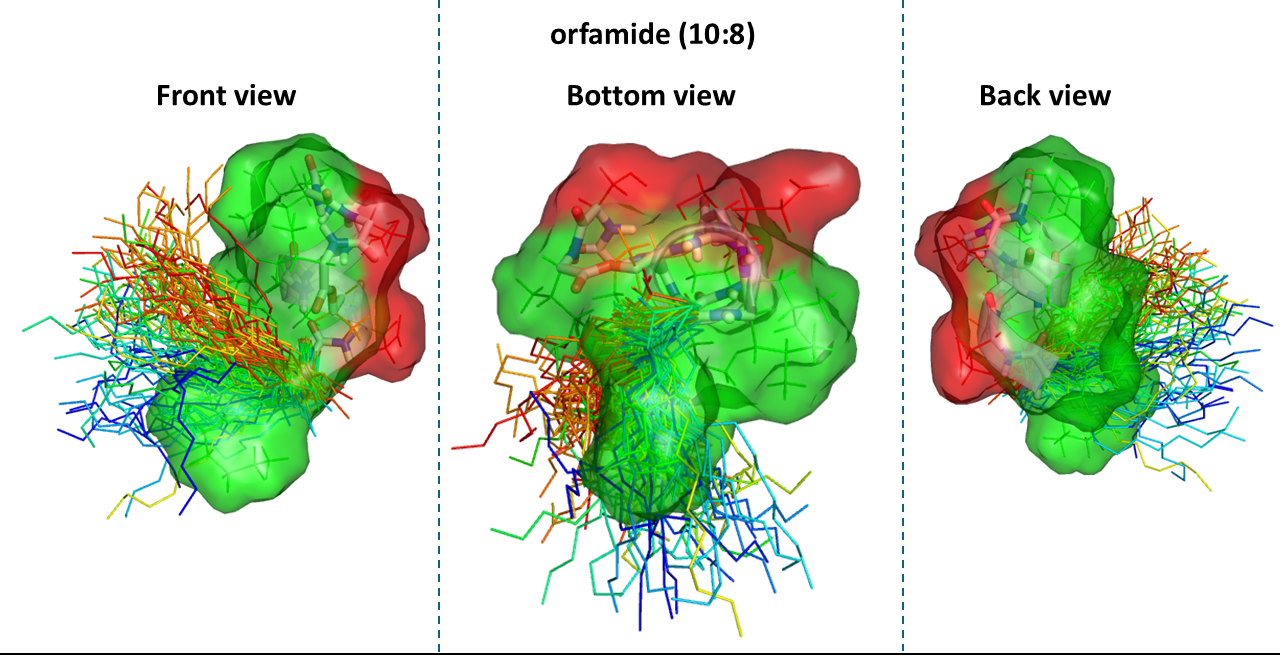


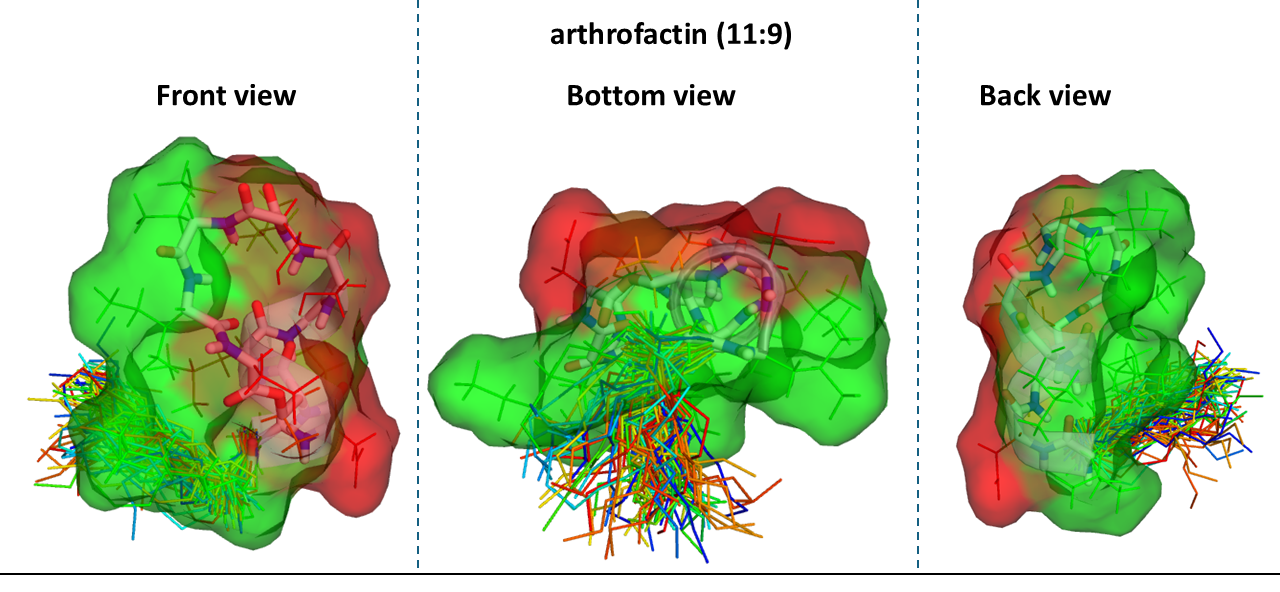


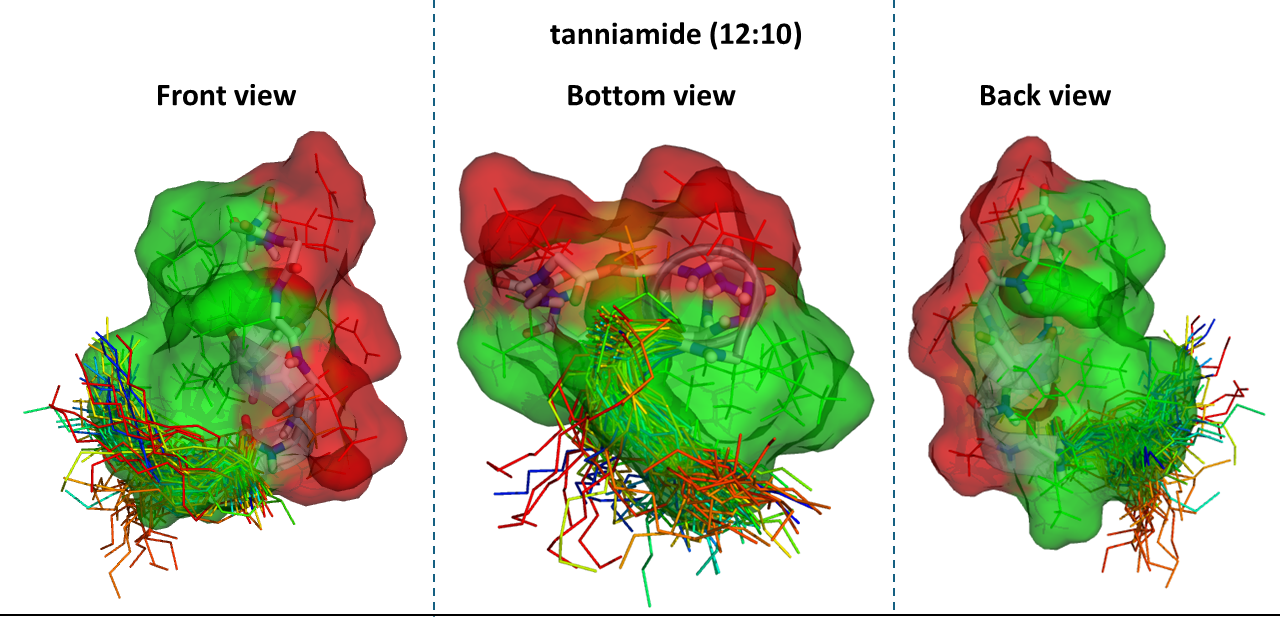


**Figure S14.** (continued)


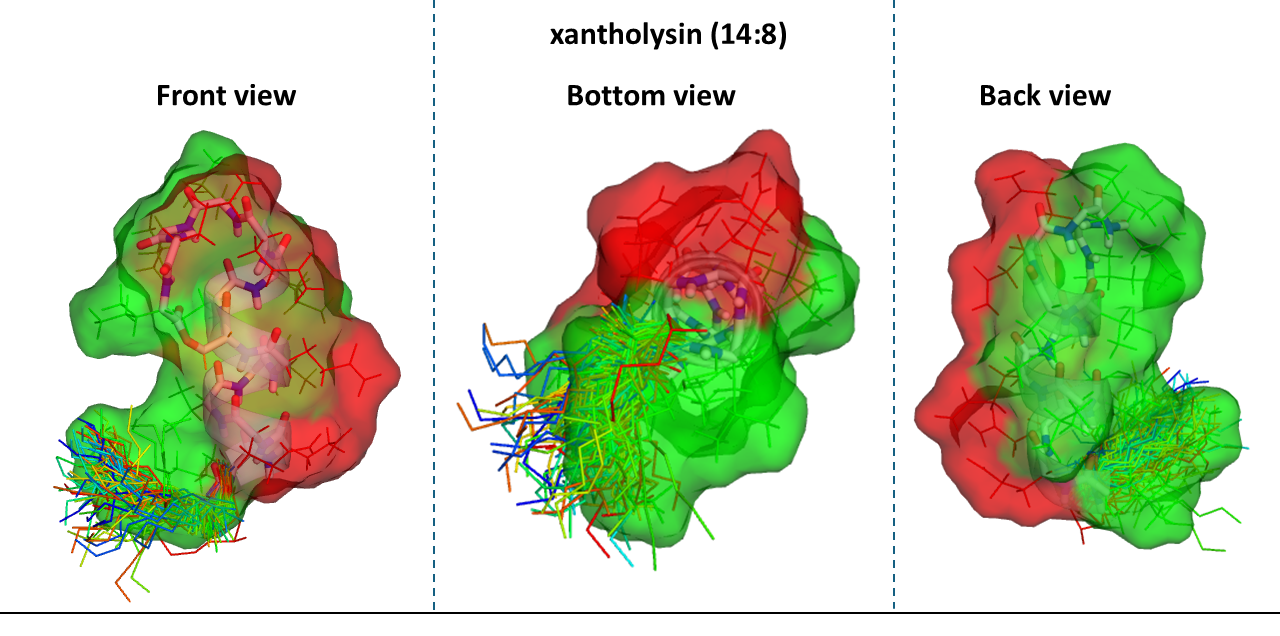


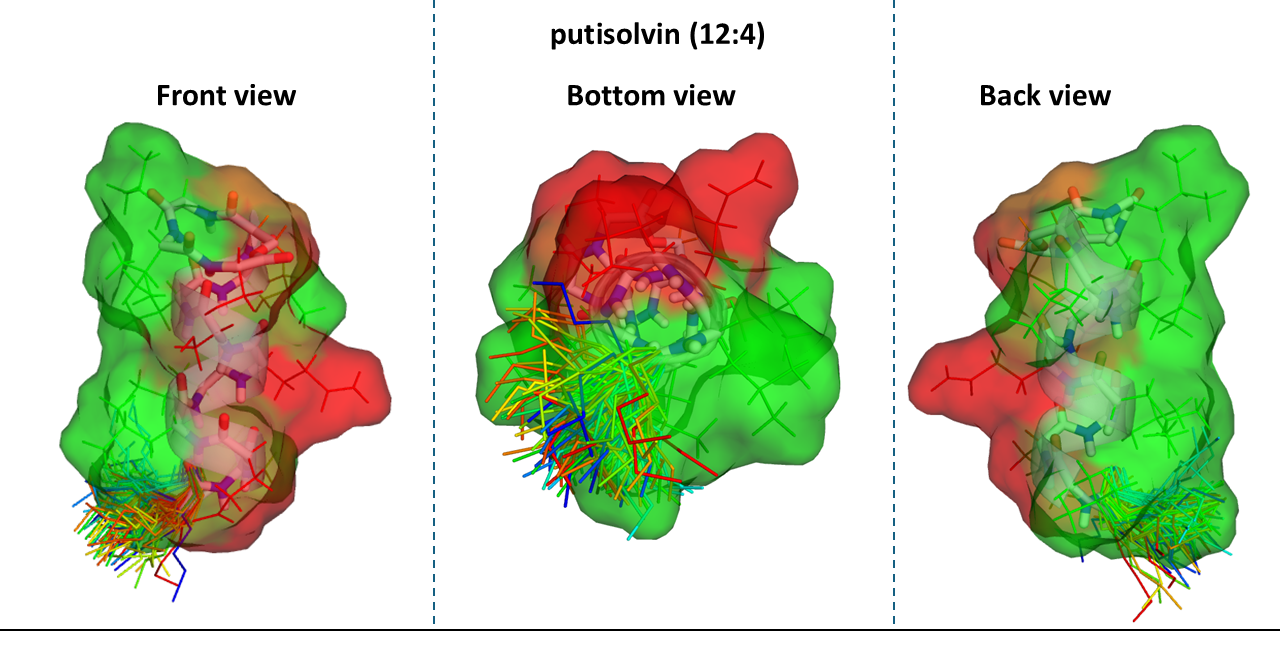


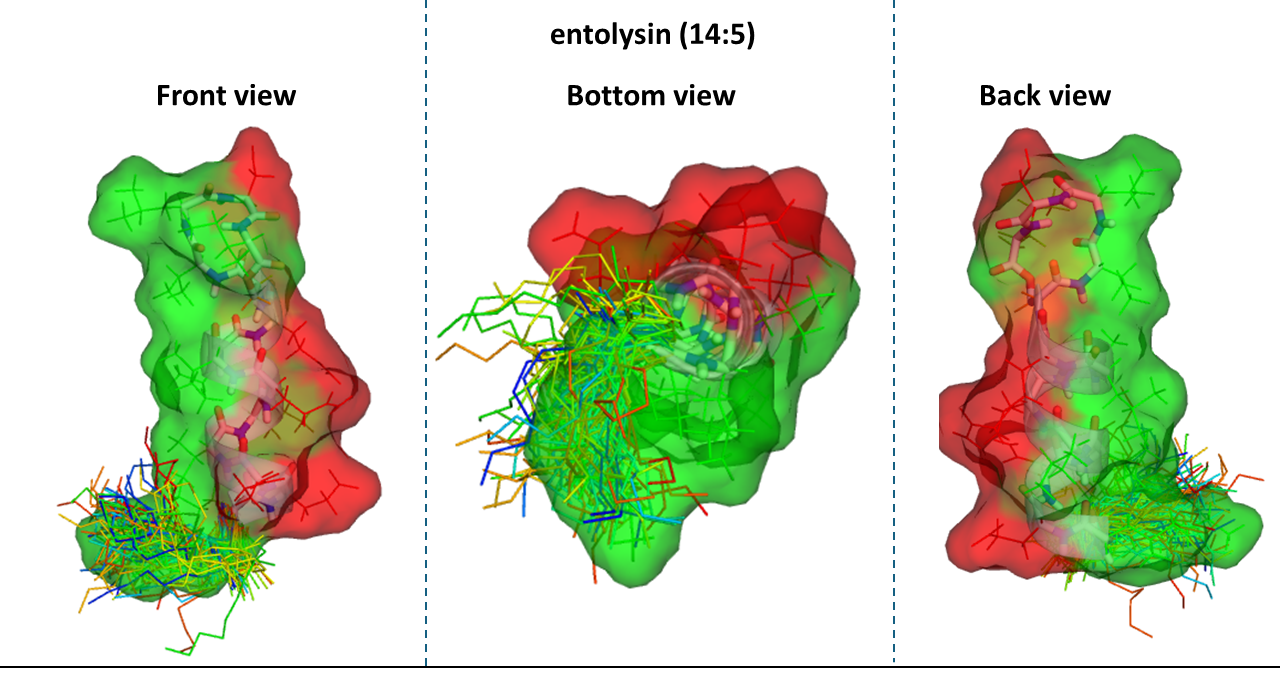


**Figure S14.** (continued)

## **4.5. Solvent exposure graphs**

The ‘watershell’ command of *cpptraj* was used to evaluate the solvent exposure of the CLiP structures in the water:DPC environment. Thus, the average number of water molecules was determined within a 5.0 Å around the Cα atom of each amino acid residue as well as multiple acyl chain atoms. The trends in solvent exposure/buriedness along the peptide as shown in SI Figure S15 are in line with the amphipathic surface character of the CLiPs.


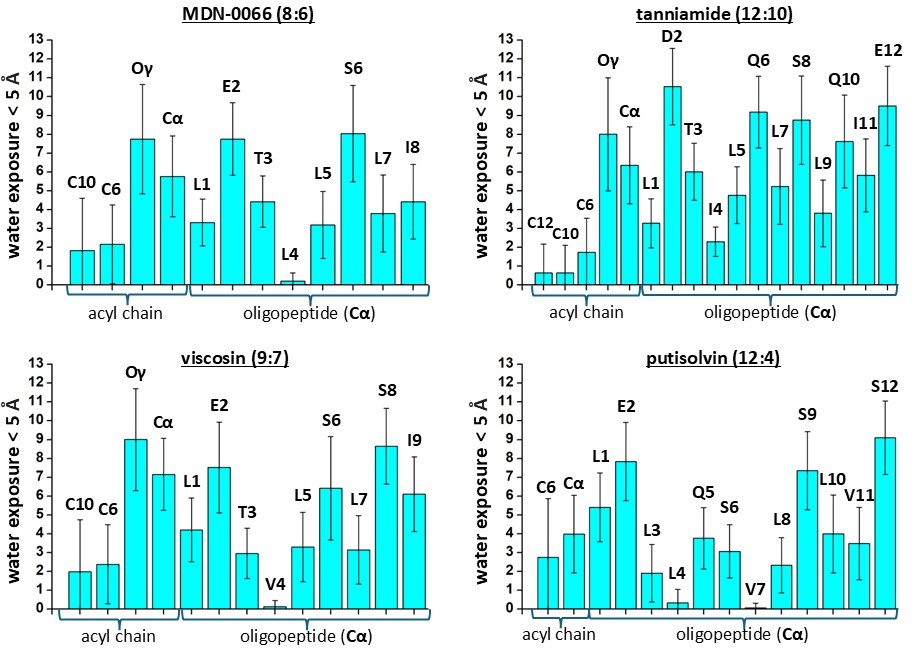


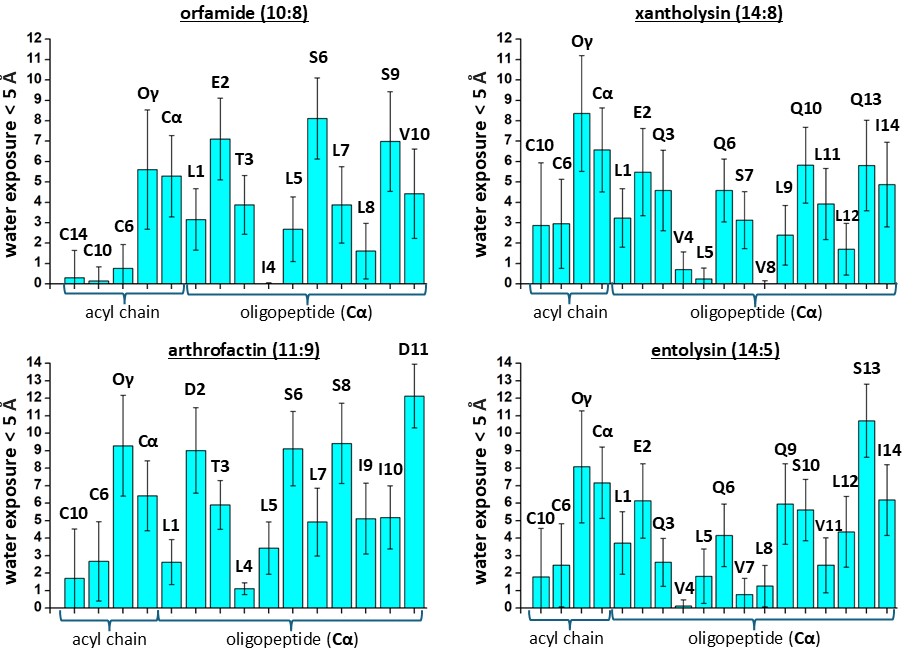


**Figure S15.** The average number of water molecules within the 5.0 Å radius sphere around particular atoms of each CLiP during the last 50 ns of the MD trajectory in water:DPC environment. Error bars are indicated as +/- the standard deviation around the average value. For amino acid residues only the respective Cα atom was chosen as a point of reference. For the acyl chain the Cα and the last C atom was considered in general, and if applicable, the Oγ, C6, and C10 atoms as well.

## **4.6. Backbone alignments of Viscosins (9:7) and Amphisins (11:9)**


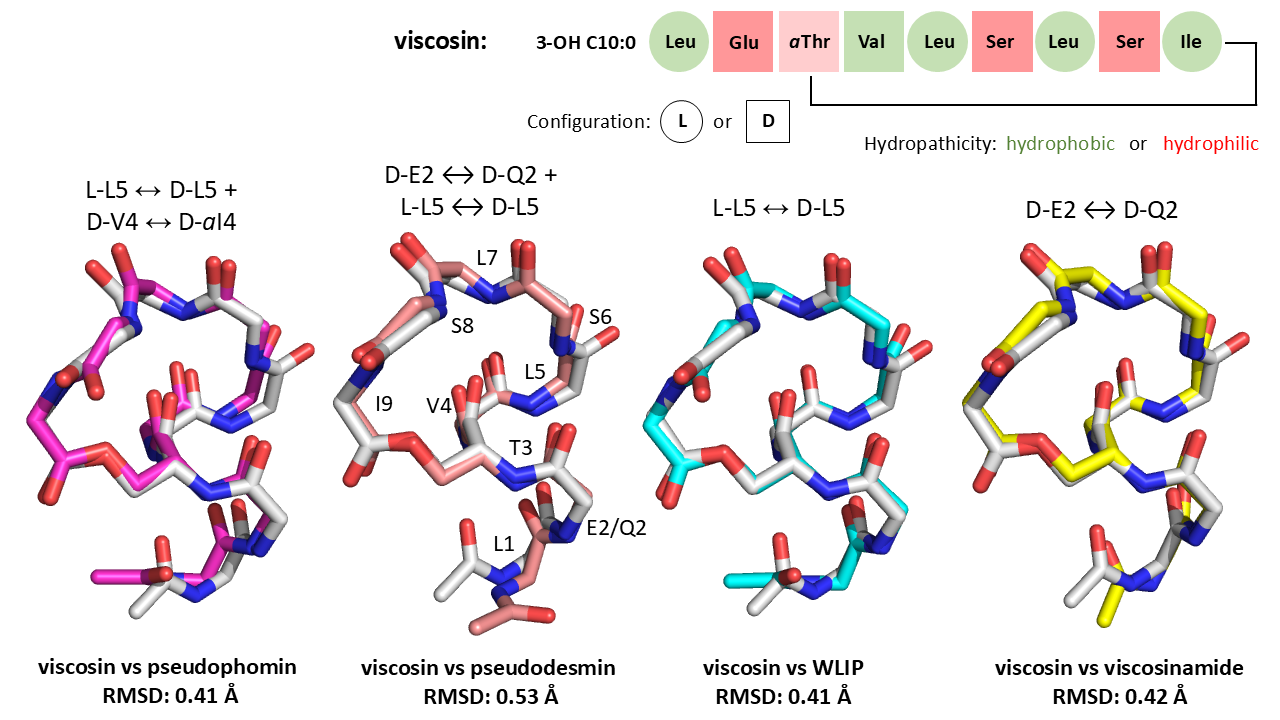


**Figure S16.** Backbone alignments of the solution conformation of viscosin (carbon atoms: grey) determined in this work to conformations of other Viscosin family (9:7) members determined in earlier works, that is, the crystal structures of pseudophomin^[26]^ (purple), pseudodesmin^[27]^ (pink), WLIP^[29]^ (turquoise) and the solution conformation of viscosinamide^[30]^ (yellow). The respective differences in the sequences of viscosin and the compared Viscosin group members and their mutual residual backbone RMSD after alignment are also indicated. The acyl chain moiety and the amino acid side-chains are always omitted for clarity except for the atoms included in the depsi bond.

**
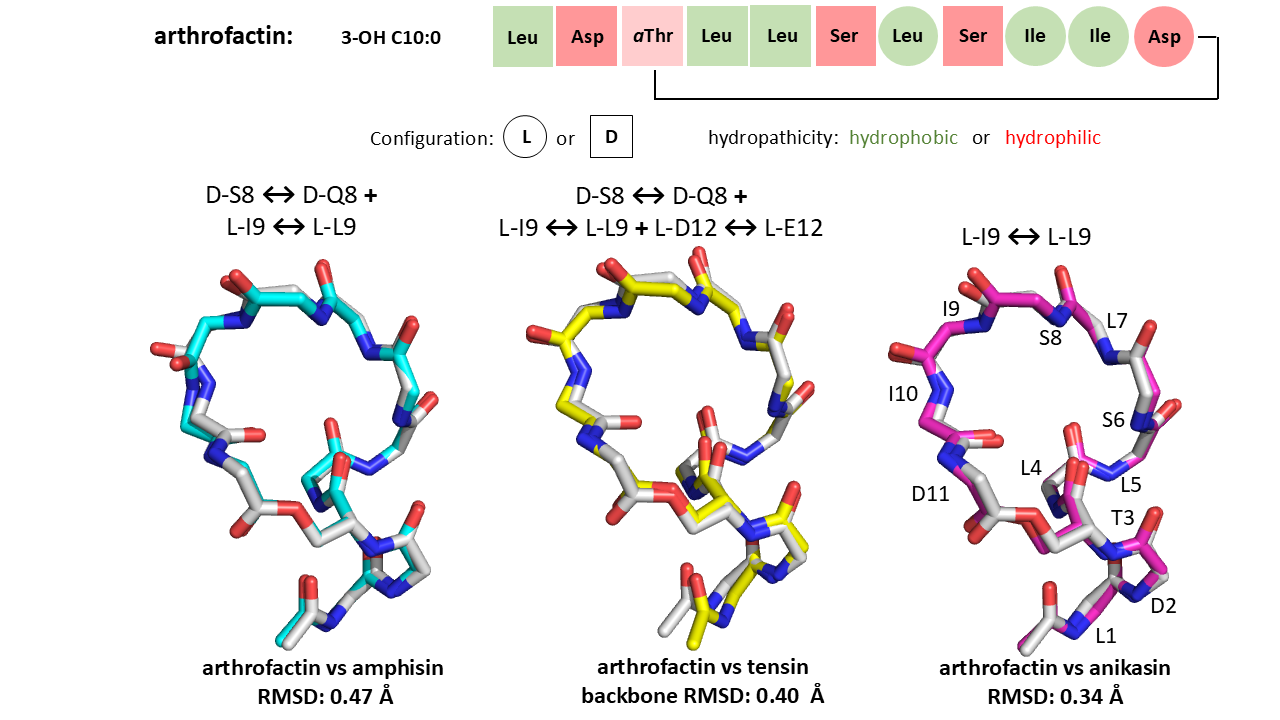
**

**Figure S17.** Backbone alignments of the solution conformation of arthrofactin (carbon atoms: grey) determined in this work to that of other Amphisin family (11:9) members determined in earlier works, that is, the crystal structures of amphisin^[40]^ (turquoise), tensin^[41]^ (yellow) and anikasin^[42]^ (purple). The respective differences in the sequences of viscosin and the compared Amphisin group members and their mutual residual backbone RMSD after alignment are also indicated. The acyl chain moiety and the amino acid side-chains are always omitted for clarity except for the atoms included in the depsi bond.

**Table S25.** Per residue comparison of the frame-to-frame averages of the simulated phi, psi angle values in the respective macrocycles of orfamide (10:8) vs xantholysin (14:8). For the analysis we considered the trajectory parts where the CLiP was already inserted into the DPC micelle (~90 ns of simulation time in both cases). For the standard deviations of these values see Table S14.

| Macrocycle position # | orfamide (10:8) | | xantholysin (14:8) | | Differences | |
| --- | --- | --- | --- | --- | --- | --- |
|  | *φ* [^o^] | *ψ* [^o^] | *φ* [^o^] | *ψ* [^o^] | ∆*φ* [^o^] | ∆*ψ* [^o^] |
| 1 (depsi) | 61.8 | 47.5 | 60.8 | 47.4 | 1.0 | 0.1 |
| 2 | 64.3 | 49.7 | 65.2 | 48.2 | 0.9 | 1.5 |
| 3 | 66.0 | 18.3 | 69.0 | 21.4 | 3.0 | 3.1 |
| 4 | 71.7 | –6.4 | 74.5 | –9.3 | 2.8 | 2.9 |
| 5 | –88.4 | –33.8 | –85.0 | –40.1 | 3.4 | 6.3 |
| 6 | –89.3 | –19.6 | –83.1 | –13.0 | 6.2 | 6.6 |
| 7 | 76.4 | –2.3 | 77.5 | –20.1 | 1.1 | 17.8 |
| 8 (depsi) | –113.9 | * | –96.0 | * | 17.9 | * |

**The torsion angle defined by the C-terminal N(i)–Cα(i)–C’(i)–OG atoms (depsi bond) showed increased flexibility for orfamide and xantholysin (SI Figure S4), thus, we omitted the comparison of the respective values.*

The level of identity in macrocycle conformation between orfamide and xantholysin is well reflected by comparing backbone phi and psi values averaged over the respective MD trajectories in the table above. The deviation from the classical α_L_-helical organization at the d-Gln10 of xantholysin (frame-to-frame MD averages: *φ*=74.5^o^; *ψ*=–9.3^o^) is almost identical to the one for d-Ser6 in orfamide (71.7^o^; –6.4^o^). Further along the loop, the phi, psi angle values continue to display excellent match throughout as they differ by a few degrees only. Somewhat larger differences (~18^o^) are only observed for the final two residues in the sequence and are accompanied by increased backbone torsion angle variability in both CLiPs. (see also SI Figure S4)

# 5. Pentorfamide conformation analysis


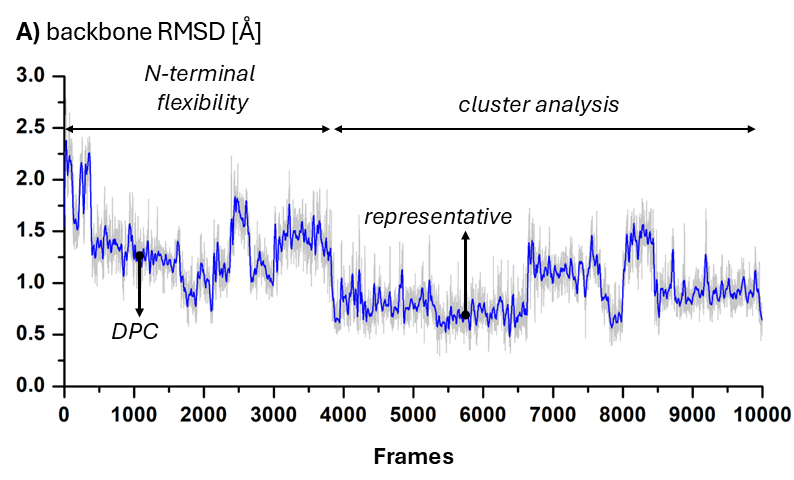


**
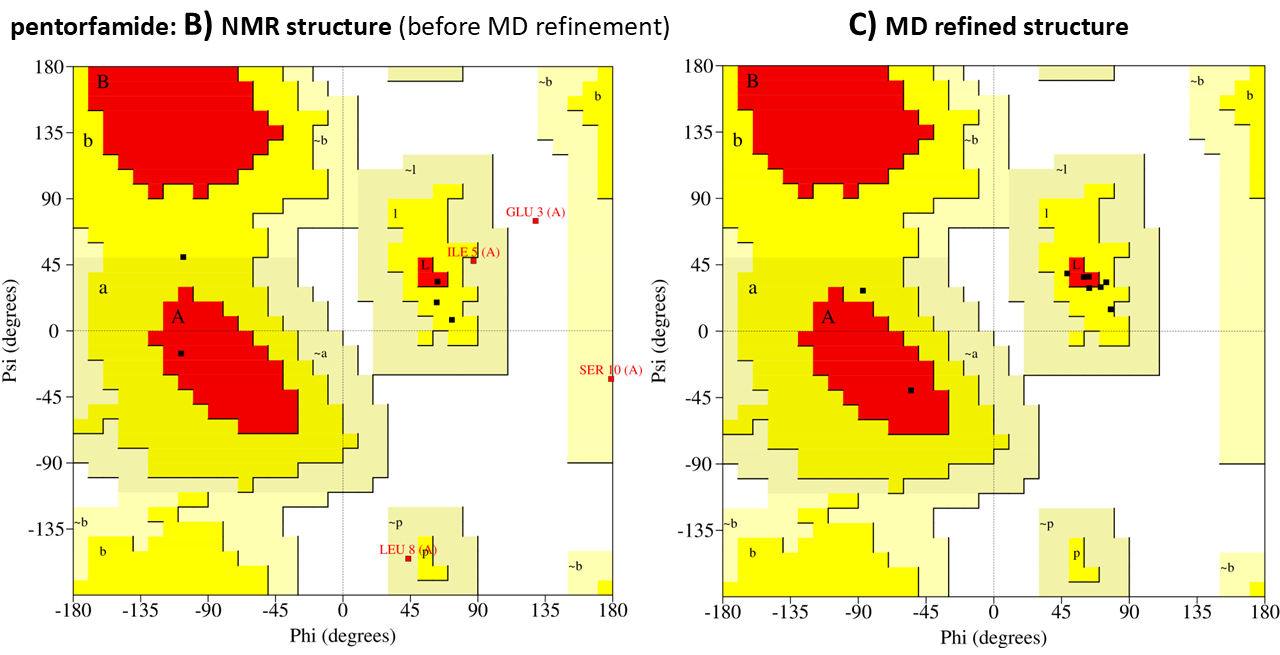
**

**Figure S18. A)** The evolution of **pentorfamide (10:5)** backbone RMSD during the 100 ns molecular dynamics trajectories in water:DPC environment compared to the last frame structure. Grey: raw data, blue: smoothed data. The time points when the CLiP molecule and the DPC micelle associate (֕”DPC”); and the frame of the representative CLiP structure of the MD simulation (֕”representative”) are highlighted. Note that opposed to the natural CLiPs we restricted the cluster analysis for pentorfamide to the last 60 ns of the simulation due to initial N-terminal rearrangements. PROCHECK Ramachandran plots **B)** before MD refinement **C)** MD refined structure used for further analysis and comparison. Residues in disallowed or generously allowed regions are indicated in red. Note that for the PROCHECK analysis the acyl chain was considered as the first residue thus all amino acid residue numbers are shifted accordingly. **D)** Visual comparison of the structures with the mutual backbone RMSD. Upon refinement the N-terminal residues as well as the macrocycle conformation/orientation are clearly readapted. The acyl chain residue was omitted from the RMSD calculation and visualization.

**
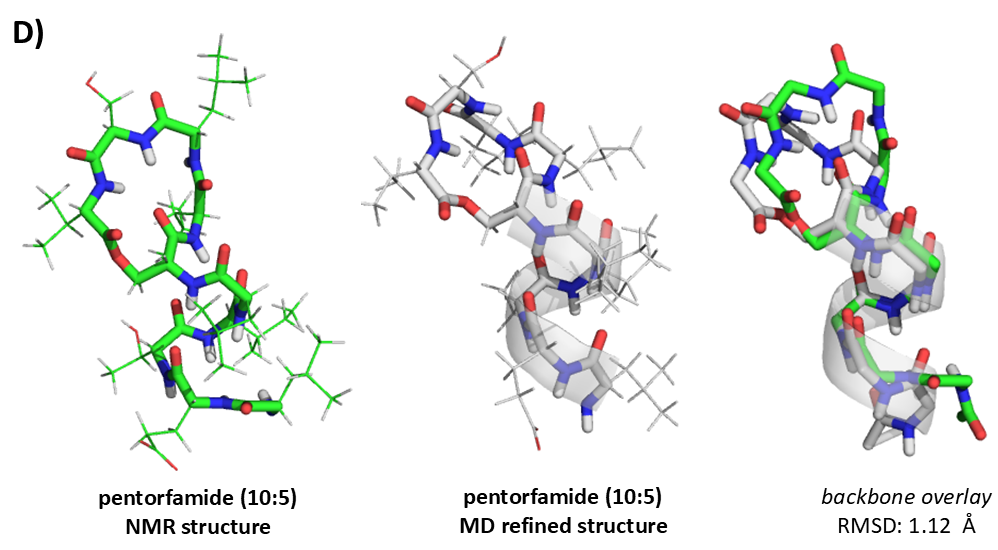
**

**Figure S18.** (continued)

**Table S26.** PROCHECK Ramachandran plot statistics of **pentorfamide (10:5)**.

| **pentorfamide (10:5)** | NMR structure | | MD refined structure | |
| --- | --- | --- | --- | --- |
| Residues in most favored regions [A,B,L] | 2 | 22.2% | 3 | 33.3% |
| Residues in additional allowed regions [a, b,l,p] | 3 | 33.3% | 6 | 66.6% |
| Residues in generously allowed regions [~a,~b,~l,~p] | 3 | 33.3% | 0 | 0% |
| Residues in disallowed regions | 1 | 11.1% | 0 | 0% |
| Number of non-glycine and non-proline residues | 9 | 100% | 9 | 100% |
| Number of end residues (excl. Gly and Pro) | 2 |  | 2 |  |
| Total # of residues identified by PROCHECK | 11 |  | 11 |  |

*The C-terminal residue l-Val is omitted from the Ramachandran maps and not considered for these statistics.*

**Table S27.** Phi/psi torsion angle data of the MD refined pentorfamide (10:5) structure. For the frame-to-frame average values only the last 60 ns of the trajectory was considered due to the initial N-terminal flexibility of the structure.

| **pentorfamide (10:5)** | | | |
| --- | --- | --- | --- |
| ***φ***/^o^ | | ***ψ***/^o^ | |
| **Rep.**  **Struct.** | **MD Average**  **[StDev]** | **Rep.**  **Struct.** | **MD Average**  **[StDev]** |
| **l-Leu1** | | | |
| 63.4 | 49.2  [12.7] | 29.2 | 35.8  [15.3] |
| **d-Glu2** | | | |
| 48.7 | 61.0  [10.0] | 39.1 | 25.1  [9.7] |
| **d-*allo*-Thr3** | | | |
| 63.1 | 65.4  [11.0] | 36.9 | 42.0  [10.9] |
| **d-*allo*-Ile4** | | | |
| 74.7 | 71.0  [10.6] | 33.1 | 35.7  [13.7] |
| **d-Leu5** | | | |
| 71.0 | 71.8  [12.3] | 29.9 | 19.4  [23.8] |
| **d -Ser6** | | | |
| 59.8 | 75.6  [36.9] | 36.7 | 43.0  [15.0] |
| **l-Leu7** | | | |
| –55.0 | –68.1  [15.7] | –40.2 | –37.1  [13.3] |
| **l-Leu8** | | | |
| –86.9 | –91.8  [18.7] | 27.5 | 56.6  [41.8] |
| **d-Ser9** | | | |
| 77.6 | 79.5  [17.0] | 14.8 | –29.2  [36.8] |
| **l-Val10** | | | |
| –148.5 | –127.7  [37.4] | –31.5 | 7.3  [71.1] |

**Table S28.** A comparative table on the phi/psi torsion angle data of the simulated pentorfamide (10:5), orfamide (10:8) and entolysin (14:5) structures considering the membrane-associated trajectory parts with explicit DPC micelle.

| Amino Acid | **pentorfamide (10:5)** | | **orfamide (10:8)** | | Amino Acid | **entolysin (14:5)** | |
| --- | --- | --- | --- | --- | --- | --- | --- |
|  | MD Average [StDev] | | MD Average [StDev] | |  | MD Average [StDev] | |
|  | *φ*/^o^ | *ψ*/^o^ | *φ*/^o^ | *ψ*/^o^ |  | *φ*/^o^ | *ψ*/^o^ |
|  |  |  |  |  | l-L1 | 51.5  [11.2] | 44.8  [12.8] |
|  |  |  |  |  | d-E2 | 63.7  [10.3] | 26.8  [10.5] |
|  |  |  |  |  | d-Q3 | 74.9  [11.8] | 36.2  [10.8] |
|  |  |  |  |  | d-V4 | 68.4  [11.6] | 37.4  [9.2] |
| l-L1 | 49.2  [12.7] | 35.8  [15.3] | 47.3  [9.9] | 52.7  [11.7] | d-L5 | 61.3  [8.8] | 34.8  [9.1] |
| d-E2 | 61.0  [10.0] | 25.1  [9.7] | 63.9  [9.3] | 31.4  [9.8] | d-G6 | 65.9  [9.5] | 34.8  [9.8] |
| d-*a*T3 | 65.4  [11.0] | 42.0  [10.9] | 61.8  [10.4] | 47.5  [9.3] | d-V7 | 67.1  [9.9] | 45.6  [8.3] |
| d-*a*I4 | 71.0  [10.6] | 35.7  [13.7] | 64.3  [9.2] | 49.7  [9.2] | d-L8 | 62.0  [8.6] | 34.4  [10.6] |
| d-L5 | 71.8  [12.3] | 19.4  [23.8] | 66.0  [9.3] | 18.3  [12.0] | d-Q9 | 72.9  [10.9] | 24.1  [19.6] |
| d-S6 | 75.6  [36.9] | 43.0  [15.0] | 71.7  [10.4] | –6.4  [26.4] | d-S10 | 78.7  [24.9] | 62.3  [8.5] |
| l-L7 | –68.1  [15.7] | –37.1  [13.3] | –88.4  [24.4] | –33.8  [14.2] | l-V11 | –58.6  [9.1] | 155.1  [8.5] |
| l-L8 | –91.8  [18.7] | 56.6  [41.8] | –89.3  [19.8] | –19.6  [12.2] | l-L12 | 54.2  [7.1] | 31.2  [9.4] |
| d-S9 | 79.5  [17.0] | –29.2  [36.8] | 76.4  [12.0] | –2.3  [25.1] | l-S13 | 60.9  [7.9] | 22.4  [14.5] |
| l-V10 | –127.7  [37.4] | 7.3  [71.1] | –113.9  [35.4] | 49.3  [63.5] | l-I14 | –123.2  [19.5] | –36.0  [43.4] |

*For the C-terminal amino acid the ψ angle refers to the side-chain-backbone angle defined between the N(i)–Cα(i)–C’(i)–OG atoms.*

**
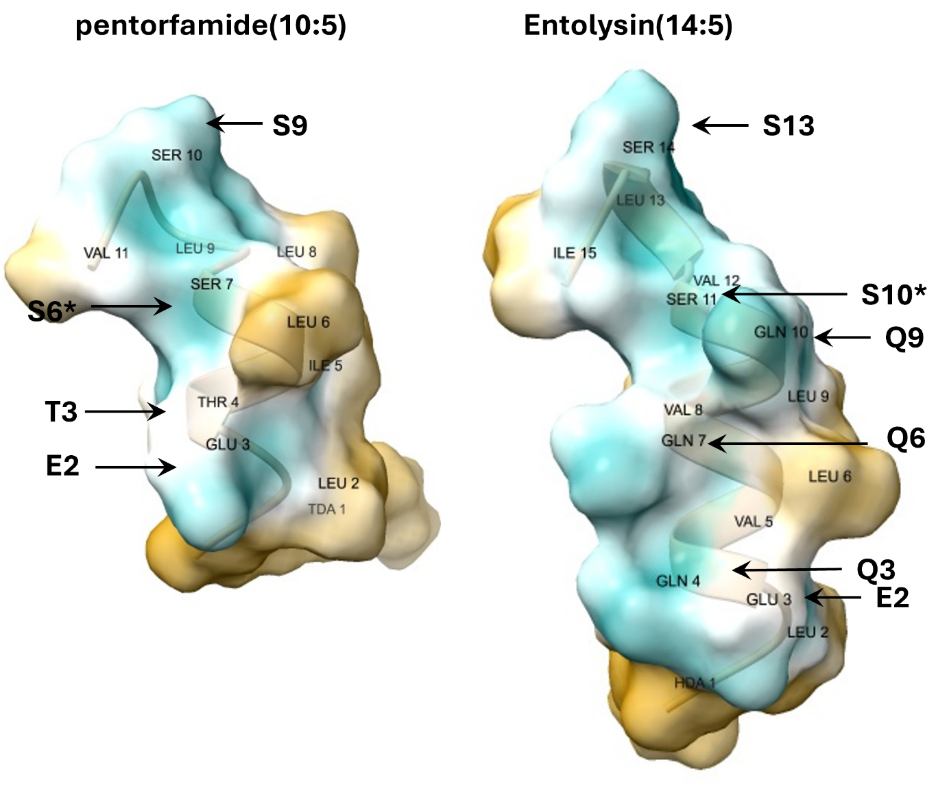
**

**Figure S19.** Surface representations of pentorfamide (10:5) vs entolysin (14:5) with aligned macrocycles. For the color code see Figure 4 in the main manuscript. The depsi bonding residue is marked with a star for both structures. The penultimate serine residue’s (d-S9 vs l-S13) side-chain is oriented similarly in the two structures despite the difference in backbone stereochemistry.


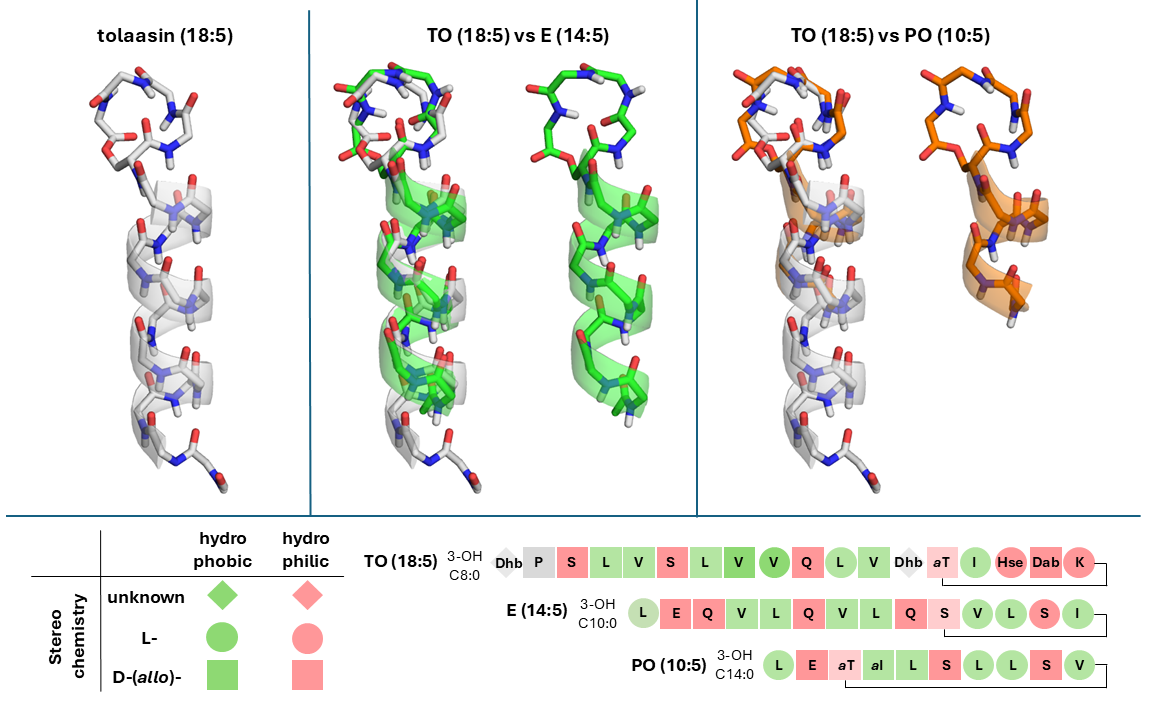


Figure S20. Alignments of the tolaasin (TO, carbon atoms: grey) vs entolysin (E, green) and pentorfamide (PO, orange) conformations. The corresponding (*l*:*m*) values are always indicated. For the sequence representations see SI Figure S1 in detail. The tolaasin conformation was determined by Jourdan *et al.*^[78]^

**Table S29.** The macrocycle phi,psi angles of the pentorfamide (10:5) conformation presented in this work vs the lowest energy tolaasin conformation presented in Jourdan *et al.*^[78]^

| **pentorfamide (10:5)** | | **tolaasin (18:5)** | | Differences (absolute values) | |
| --- | --- | --- | --- | --- | --- |
| *φ*/^o^ | *ψ*/^o^ | *φ*/^o^ | *ψ*/^o^ | Δ*φ*/^o^ | Δ*ψ*/^o^ |
| **d-Ser6** | | **d-*allo*-Thr14** | |  |  |
| 59.8 | 36.7 | 86.6 | 43.4 | 26.8 | 6.7 |
| **l-Leu7** | | **l-Ile15** | |  |  |
| –55.0 | –40.2 | –55.6 | –38.9 | 0.6 | 1.3 |
| **l-Leu8** | | **l-Hse16** | |  |  |
| –86.9 | 27.5 | –88.1 | 24.8 | 1.2 | 2.7 |
| **d-Ser9** | | **d-Dab17** | |  |  |
| 77.6 | 14.8 | 80.0 | –54.8 | 2.4 | 69.6 |
| **l-Val10** | | **l-Lys18** | |  |  |
| –148.5 | –31.5 | –62.0 | 104.6 | 86.5 | 136.1 |

#

# References

[88] E. Sikorska, D. Wyrzykowski, K. Szutkowski, K. Greber, E. A. Lubecka, I. Zhukov, "Thermodynamics, size, and dynamics of zwitterionic dodecylphosphocholine and anionic sodium dodecyl sulfate mixed micelles” *J. Therm. Anal. Calorim.* **2016**, *123*, 511–523

[89] J. Lauterwein, C. Bösch, L. R. Brown, K. Wüthrich, “Physicochemical studies of the protein-lipid interactions in melittin-containing micelles” *Biochim. Biophys. Acta - Biomembr.* **1979**, *556*, 244–264

[90] S. Akoka, L. Barantin, M. Trierweiler, "Concentration measurement by proton NMR using the ERETIC method” *Anal. Chem.* **1999**, *71*, 2554–2557

[91] T. L. Hwang, A. J. Shaka, "Water suppression that works. Excitation sculpting using arbitrary wave-forms and pulsed-field gradients” *J. Magn. Reson.* **1995**, *112*, 275–279

[92] D. Sinnaeve, "The Stejskal–Tanner equation generalized for any gradient shape—an overview of most pulse sequences measuring free diffusion” *Concepts Magn. Reson. Part A* **2012**, *40*, 39–65

[93] W. F. Vranken, W. Boucher, T. J. Stevens, R. H. Fogh, A. Pajon, M. Llinas, E. L. Ulrich, J. L. Markley, J. Ionides, E. D. Laue, "The CCPN data model for NMR spectroscopy: development of a software pipeline” *Proteins* **2005**, *59*, 687–696

[94] S. P. Skinner, B. T. Goult, R. H. Fogh, W. Boucher, T. J. Stevens, E. D. Laue, G. W. Vuister, "Structure calculation, refinement and validation using CcpNmr Analysis” *Acta Crystallogr. D* **2015**, *71*, 154–161

[95] D. A. Case, V. Babin, J. T. Berryman, R. M. Betz, Q. Cai, D. S. Cerutti, T. E. Cheatham, III, T .A. Darden, R. E. Duke, H. Gohlke, A. W. Goetz, S. Gusarov, N. Homeyer, P. Janowski, J. Kaus, I. Kolossváry, A. Kovalenko, T. S. Lee, S. LeGrand, T. Luchko, R. Luo, B. Madej, K. M. Merz, F. Paesani, D. R. Roe, A. Roitberg, C. Sagui, R. Salomon-Ferrer, G. Seabra, C.L. Simmerling, W. Smith, J. Swails, R.C. Walker, J. Wang, R. M. Wolf, X. Wu, P. A. Kollman, AMBER 14 University of California, San Francisco, **2014**

[96] M. J. Frisch, G. W. Trucks, H. B. Schlegel, G. E. Scuseria, M. A. Robb, J. R. Cheeseman, G. Scalmani, V. Barone, B. Mennucci, G. A. Petersson, H. Nakatsuji, M. Caricato, X. Li, H. P. Hratchian, A. F. Izmaylov, J. Bloino, G. Zheng, J. L. Sonnenberg, M. Hada, M. Ehara, K. Toyota, R. Fukuda, J. Hasegawa, M. Ishida, T. Nakajima, Y. Honda, O. Kitao, H. Nakai, T. Vreven, Jr J. A. Montgomery, J. E. Peralta, F. Ogliaro, , M. Bearpark, J. J. Heyd, E. Brothers, K. N. Kudin, V. N. Staroverov, , R. Kobayashi, J. Normand, K. Raghavachari, A. Rendell, J. C. Burant, S. S. Iyengar, J. Tomasi, M. Cossi, N. Rega, J. M. Millam, M. Klene, J. E. Knox, J. B. Cross, V. Bakken, C. Adamo, J. Jaramillo, R. Gomperts, R. E. Stratmann, O. Yazyev, A. J. Austin, R. Cammi, C. Pomelli, J. W. Ochterski, R. L. Martin, K. Morokuma, V. G. Zakrzewski, G. A. Voth, P. Salvador, J. J. Dannenberg, S. Dapprich, A. D. Daniels, Ö. Farkas, J. B. Foresman, J. V. Ortiz, J. Cioslowski, D. J. Fox, Gaussian 09, Revision D.01, Gaussian, Inc. Wallingford CT, **2009**

[97] J. Wang, W. Wang, P. A. Kollman, D. A. Case, "Automatic atom type and bond type perception in molecular mechanical calculations” *J. Mol. Graph.*, **2006**, *25*, 247–260

[98] J. Wang, R. M. Wolf, J. W. Caldwell, P. A. Kollman, D. A. Case, “Development and testing of a general amber force field” *J. Comput. Chem.* **2004**, *25*, 1157–1174

[99] C. J. Dickson, B. D. Madej, A. A. Skjevik, R. M. Betz, K. Teigen, I. R. Gould, R. C. Walker, "Lipid14: the amber lipid force field” *J. Chem. Theory Comput.* **2014**, *10*, 865–879

[100] J. P. Ryckaert, G. Ciccotti, H. J. C. Berendsen, "Numerical integration of the cartesian equations of motion of a system with constraints: molecular dynamics of n-alkanes” *J. Comput. Phys*. **1977**, *23*, 327–341

[101] U. Essmann, L. Perera, M. L. Berkowitz, T. Darden, H. Lee, L. G. Pedersen, "A smooth particle mesh Ewald method” *J. Chem. Phys*. **1995**, *103*, 8577–8593

[102] W. Humphrey, A. Dalke, K. Schulten, "VMD: visual molecular dynamics” *J. Mol. Graph*. **1996**, *14*, 33–38

[103] D. R. Roe, T. E. Cheatham, "PTRAJ and CPPTRAJ: software for processing and analysis of molecular dynamics trajectory data” *J. Chem. Theory Comput.* **2013***,* *9*, 3084–3095

[104] R. A. Laskowski, M. W. MacArthur, D. S. Moss, J. M. Thornton, “PROCHECK: a program to check the stereochemical quality of protein structures” *J. App. Cryst*. **1993**, *26*, 283–291

[105] R. A. Laskowski, J.A.C. Rullmann, M.W. MacArthur, R. Kaptein, J. M. Thornton, "AQUA and PROCHECK-NMR: programs for checking the quality of protein structures solved by NMR” *J. Biomol. NMR* **1996**, *8*, 477–486

[106] E. C. Meng, T. D. Goddard, E. F. Pettersen, G. S. Couch, Z. J. Pearson, J. H. Morris, T. E. Ferrin, "UCSF ChimeraX: Tools for structure building and analysis” *Protein Sci.* **2023**, 32, e479
